# Vibration-assisted fabrication of thin shells with spatially distributed imperfections

**DOI:** 10.1038/s41467-026-73343-2

**Published:** 2026-05-20

**Authors:** Ilyes Krida, Jacob Tang, Leo Mangalath, Daniel Floryan, Tian Chen

**Affiliations:** 1 Department of Mechanical & Aerospace Engineering, University of Houston, Houston, TX USA; 2 Department of Aerospace & Mechanical Engineering, University of Southern California, Los Angeles, CA USA; 3 Department of Aeronautics, Imperial College London, London, UK

**Keywords:** Mechanical properties, Design, synthesis and processing, Mechanical engineering

## Abstract

Thin-shell structures, found in biological systems such as beetle carapaces and widely used in aerospace and civil engineering, achieve remarkable strength-to-mass ratios given their slenderness and curved geometries. However, their load-bearing capacity is highly sensitive to geometric imperfections, which are often unavoidable during fabrication and can trigger subcritical buckling. Silicone-based hemispherical domes have served as an experimental surrogate to study this phenomenon, yet prior work has largely focused on localized imperfections, failing to capture the spatially distributed nature of real-world imperfection patterns. Here, we introduce a vibration-assisted method for fabricating thin shells with spatially distributed, mode-shaped imperfections. Silicone is cast onto a thick elastic mold excited by a speaker, and vibration-induced flow during curing creates thickness variations. High-speed imaging and destructive measurements reveal material accumulation at the antinodes of the mold’s vibrational modes. The engineered imperfections can be tuned by excitation frequency and mold shape, while their amplitude increases with speaker volume. Buckling experiments demonstrate significant reductions in critical pressure, offering a scalable platform to study and tune imperfection-sensitivity. Beyond shell mechanics, this method enables patterning of soft materials for applications ranging from morphable surfaces to bioinspired design.

## Abstract (web summary)

The study presents a vibration-assisted method to fabricate thin shells with tunable, spatially distributed thickness imperfections, showing that vibration mode shapes can be imprinted and controlled, significantly affecting the shells’ buckling behaviour.

## Introduction

Thin-shell structures are ubiquitous in both biological and engineered systems, with examples spanning a wide range of length scales, including virus capsids, pollen grains, beetle exoskeletons, pressure vessels, spacecrafts, and civil infrastructure^1–4^. The mechanical advantage of thin shells stems from their curved geometry and large radius-to-thickness ratios. However, this slenderness also makes some shells highly susceptible to *subcritical buckling*, a sudden and catastrophic instability often triggered by small geometric imperfections.

This imperfection sensitivity has posed a fundamental challenge in shell mechanics for over a century. Early analyses by Zoelly^5^ provided the first closed-form expression for the critical pressure of an ideal, perfectly spherical shell, while subsequent developments by Donnell and Batdorf established governing equations and stability criteria for cylindrical and spherical shells under external pressure^6,7^. Von Kármán later demonstrated that equilibrium states involving large deflections could be sustained at loads far below the classical prediction^8–10^. Tsien further attributed the discrepancy between theory and experiment to the extreme instability of the postbuckling path in the presence of arbitrary disturbances^11^. A unifying theoretical framework was established by Koiter, whose asymptotic postbuckling theory demonstrated that infinitesimal geometric imperfections can induce dramatic reductions in critical load^12^. Following this, extensive analytical and numerical studies sought to reconcile theory and experiment by examining the influence of thickness variations, nonuniform loading, boundary conditions, pre-buckling deformations, and geometric imperfections^13,14^. Focusing on cylindrical shells, Babcock performed systematic comparisons of different imperfections and concluded that geometric imperfections dominate buckling sensitivity^15^.

Despite these advances, experimental progress has historically lagged behind theoretical and computational developments. As a result, practical shell design continues to rely on classical predictions augmented by empirical knockdown factors^8^. While careful experimental validation has been achieved for cylindrical shells and, in fewer cases, spherical shells^12,13^, traditional fabrication methods often introduce uncontrolled and stochastic defect fields, making deterministic links between imperfection geometry and load-bearing capacity difficult to establish. This limitation has motivated statistical approaches to shell buckling^16^, as well as recent efforts to fabricate shells with controlled, prescribed imperfections^17–20^.

The recent resurgence of interest in shell buckling is partly driven by the advent of novel fabrication and experimental techniques that enable the controlled introduction of imperfections into otherwise near-perfect shells^21^. In these works, the term “imperfections” is used in the shell mechanics sense to denote deviations from an idealized perfect geometry, including deliberately prescribed features introduced to probe imperfection-sensitive buckling behavior. Rather than experimenting with full-scale structures, desktop-sized silicone casts have been adopted as an experimental model system^17,22^. These studies have established an experimental framework for imperfection-sensitive buckling of spherical shells, including quantitative prediction of knockdown factors for precisely engineered dimple-like defects^22^, characterization of large-amplitude defect plateaus^23^, buckling under probing forces^18^, comparison of dimpled versus bumpy defects^19^, probabilistic buckling of shells with distributions of defects^24^, and defect–defect interactions in multi-defect shells^25,26^. While recent works have begun investigating multiple interacting imperfections^25,27^, it remains difficult for such fabrication methods to reproduce the complex, global imperfection fields that arise naturally from manufacturing variability. Moreover, constructing dedicated fabrication setups for all possible imperfection geometries remains impractical.

Here, we introduce a vibration-assisted fabrication method to create thin hemispherical shells with prescribed global imperfection fields shaped by the vibrational modes of their casting mold. Vibrational excitations have long been used to impose patterns in physical systems. Chladni plate experiments, where fine particles accumulate along the nodal lines of a vibrating surface, illustrate how modal patterns emerge from standing wave excitation^28^. In fluid systems, Faraday waves are standing waves on a fluid surface excited by vertical vibration that create rich patterns through nonlinear resonances and symmetry breaking^29^. In manufacturing, vibration has been harnessed to assist in powder spreading, colloidal self-assembly, and material redistribution in soft or particulate media^30–34^. Central to these effects is the coupling between oscillatory driving and the medium’s response, often mediated by steady secondary streaming flows that persist over longer timescales than the driving frequency^35^.

In our method, by actively exciting a thick elastic hemispherical mold with a speaker, and adjusting the excitation frequency and output volume, we induce resonant modal deformation of the mold’s surface during the curing of a thin liquid silicone layer. This vibration generates spatially structured flows within the liquid silicone, causing material to accumulate at antinodes and to thin elsewhere, thereby imprinting the mold’s vibrational displacement field as a permanent variation in shell thickness.

We characterize the resulting imperfection fields using stereographic optical imaging, reconstructing the full scalar field of shell thickness as a function of spherical coordinates. We find that these fields qualitatively resemble the vibrational eigenmodes as predicted by FE simulations, with modal symmetry and spatial distribution closely preserved. While the geometries of the imperfections are mode-dependent, the amplitude of thickness variation can be tuned continuously by varying the acoustic excitation volume. We then perform mechanical buckling experiments under vacuum loading to measure the effect of these globally distributed imperfections on structural stability. Lastly, we demonstrate that by altering the shape of the mold itself, a broader spectrum of imperfection shapes can be achieved.

## Results

### Vibration-assisted casting

**Fig. 1:**
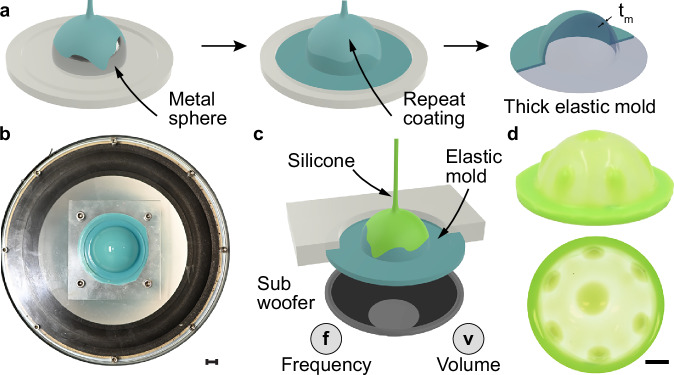
Overview of the casting and vibration-assisted fabrication protocol. **a** Fabrication of an elastic mold by repeated coating of a metal sphere. **b** Photograph of the mold attached to a PMMA plate bolted to the speaker. **c** Casting of a silicone hemispherical shell atop the elastic mold as it is being excited acoustically at a known frequency, *f*, and volume, *v*. **d** An example of a resulting imperfect shell with volume maximized to make visible the imperfections. The scale bars represent 10 mm.

To fabricate thin hemispherical shells with spatially distributed imperfections, we begin by casting a hemispherical elastic mold through repeated coating of a precisely machined metal sphere (*D* = 50.8 mm) with silicone (Mold Star 16 Fast Platinum Silicone Rubber) (Fig. 1a)^17^. By allowing the silicone to cure in-between successive coatings, the mold thickness *t*_m_ increases per coat by approximately 0.35 mm. Once we achieve a mold thickness that exceeds the thickness of the eventual hemispherical shells by an order of magnitude, we proceed with casting. This thickness ratio ensures that the vibrational response of the mold is dominated by its own elastic properties and is minimally influenced by fluid-structure coupling with the yet-uncured silicone layer.

The hemispherical elastic mold is rigidly mounted onto a circular PMMA plate (≈ 230 mm in diameter) with a circular cutout in the center matching the mold’s diameter. This plate is bolted to the rim of an acoustic speaker (Polk Audio PSW10 10” Powered Subwoofer) to form an enclosed volume (Fig. 1b). Prior to fabrication, we first determine the natural vibration modes of the elastic mold through Finite Element (FE) analysis. The frequencies used in the fabrication experiments are then chosen to coincide with these computed modal frequencies, allowing each shell to inherit the geometric signature of a specific vibrational mode. The frequency and volume of the speaker is digitally controlled with a PC. See SI Sec. 1 for the detailed fabrication protocol of the elastic mold.

To allow easy separation after casting, a mold-release (Ease Release 200, Smooth-On) is first sprayed on the elastic mold. Following this, a two-part vinyl polysiloxane (Elite Double 32, Zhermack) is mixed and poured onto the elastic mold to sufficiently cover the entire mold. The speaker is then powered on at a specific frequency and volume (Fig. 1c). The acoustics induce vibration in the mold, which redistributes the still-liquid silicone into a new steady-state prior to solidification (see SI Movie 1). As the silicone cures, this redistribution becomes permanently imprinted in the shell geometry. An example of such spatially distributed imperfections is shown in Fig. 1d. By tuning the frequency and the volume, we are able to fabricate shells of different imperfection geometries.

Note that the timescale of curing (~ 20 min) is much slower than the formation of the imperfection pattern (~ 1 to 5 s), which is itself much slower than the period of the excitation (≤0.01 s). Further, the exponential nature of curing suggests that, during the initial period following pouring, the silicone behaves predominantly as a viscous Newtonian fluid and does not exhibit shear-thickening or shear-thinning behavior^17,36^.

The significant separation of timescales between vibration-driven redistribution (~ 1 to 5 s) and curing (~ 20 min) implies that the thickness patterns form well before solidification. The final shell geometry is therefore established during the liquid phase, while curing primarily serves to “freeze” an already-formed thickness distribution. Accordingly, the observed pattern formation is governed by vibration-induced flow.

### Imperfection pattern formation

**Fig. 2:**
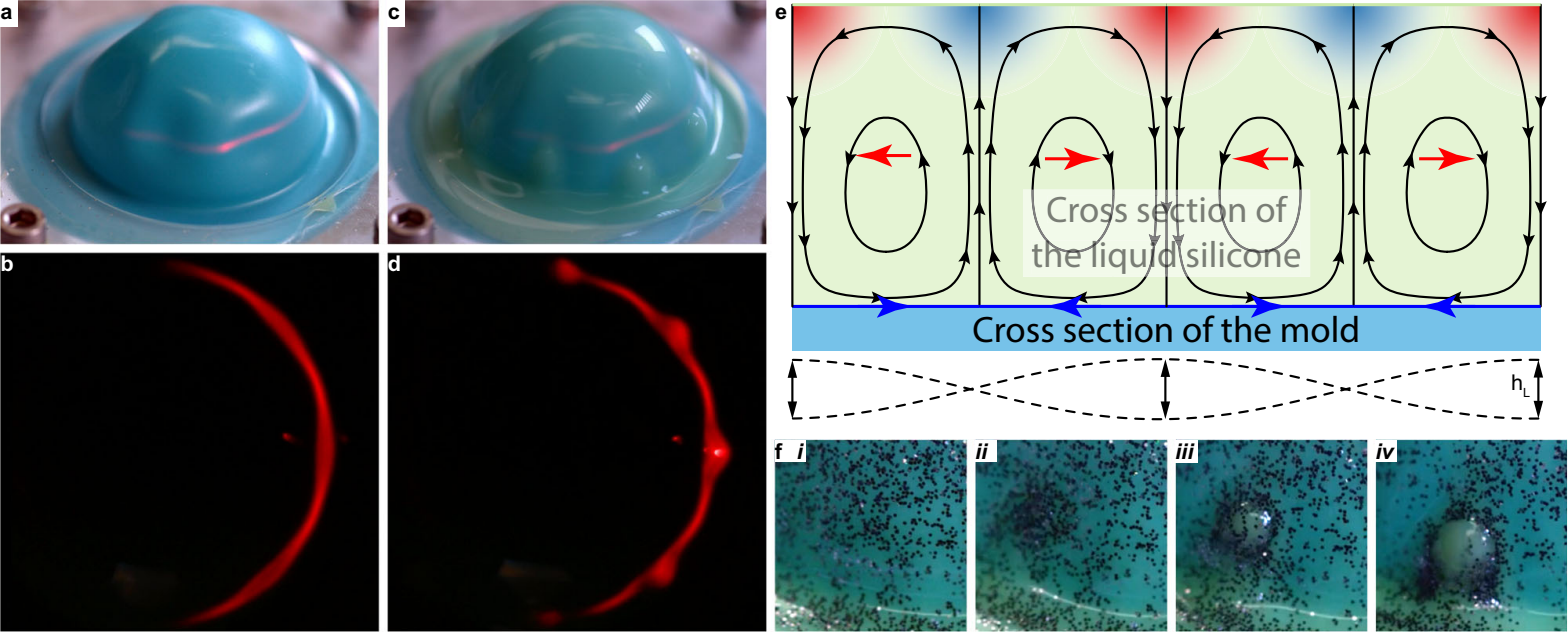
Physics of silicone casting under vibration. **a** Shell excited at a high speaker volume to make visible the periodic vibration as captured by a slow-motion camera (see SI Movie 1). **b** Projecting a horizontal laser sheet at a fixed elevation and photographing the vibrating mold from top down, we capture the locations of the nodes and antinodes of the mold. **c** Once silicone is poured onto the vibrating mold, multiple bumps are formed (see SI Movie 2). **d** With the same laser sheet, it is observed that the liquid silicone accumulates at the antinodes of the mold. **e** Sketch of the streaming flow’s pressure distribution along one segment of one circumferential ring around the shell spanning one wavelength of the vibration. Red indicates high pressure while blue indicates low pressure. The blue arrows indicate the boundary condition at the lower boundary, which leads to the circulating flow shown by the streamlines. The red arrows show the x-component of the forcing due to the Reynolds stresses. The vibration pattern is shown at the bottom for reference. **f** Stroboscopic slow motion capture (frame rate equal to the frequency of the speaker) shows a sequence of fluid flow tracked using reflective particles, i–iv captures progressive accumulation of liquid silicone at antinodes (see SI Movie 3).

We proceed to understand the formation of imperfections. Specifically, we use slow-motion videography to capture how the vibration of the mold shapes the liquid silicone as it is poured over (see SI Movie 2). We choose one modal frequency *f* = 167 Hz as informed by FE analysis featuring 8 nodes and 8 antinodes distributed around the circumference. By adjusting the speaker volume, we observe periodic deformations on the mold matching the modal shape from the FE (Fig. 2a). By setting the frequency of video capture to match that of the speaker frequency, the resulting stroboscopic video shows negligible movement, demonstrating that the mold is vibrating in sync with the speaker (see SI Movie 2).

To experimentally identify nodal and antinodal locations, we illuminate the vibrating mold with a laser sheet positioned at a fixed elevation *z* = 0.25*r*. The laser sheet intersects the mold surface along a curve; we record the motion of this intersection curve around the circumference using time-integrated imaging. Locations with minimal motion are identified as nodes, whereas locations with maximal motion are identified as antinodes (Fig. 2b).

Once silicone is poured onto the mold, the onset of acoustic-driven vibration causes the uncured silicone to flow and accumulate at distinct points (Fig. 2c). The same laser sheet photography from the top view shows that the accumulations coincide with the locations of the antinodes (Fig. 2d), i.e., where the magnitude of the vibration amplitude is at a maximum in a standing-wave pattern.

This seems counterintuitive as the antinodes experience the largest vibration amplitude and could cause the liquid to flow away. A theoretical analysis of the flow reveals that the mold’s vibrations drive an oscillatory primary flow of the same frequency and wavelength as the vibrations. The primary flow creates Reynolds stresses with half the wavelength that, together with a boundary forcing created by the primary flow, drive a secondary, time-averaged, streaming flow with half the wavelength (Fig. 2e). The associated pressure would push an undeformed free surface outward at the locations of the antinodes, leading the liquid silicone to redistribute toward them. See SI Sec. 5 for more details.

To experimentally visualize the dynamics of this flow, we introduce reflective particles on the surface of the uncured silicone. Slow-motion imaging captures the progressive migration of particles toward antinodes during curing from all directions on the surface (Fig. 2f), driven by a secondary mean streaming flow, as predicted by the theory. Once the bumps are formed over a time period ~ 5 s (that is much longer than the period of the vibration), an internal toroidal circulation is observed from the motion of the particles that matches those predicted in Fig. 2e. Similar to vortex rings, the liquid silicone moves to the circumference of the bump before moving under and emerging at the apex (see SI Movie 3). This secondary streaming flow is much weaker than the primary oscillatory flow, and bears resemblance to the streaming flow associated with Faraday waves^35^. Here, gravity may bias the overall symmetry of the flow by acting as a body force on the accumulating fluid. If the speaker is shut off or tuned to another frequency, the fluid migrates accordingly until curing occurs in approximately 20 min, at which point the shell is peeled off the mold. This allows us to correlate the modal vibration to the eventual shape of the shells cast upon the elastic mold.

### Quantification of imperfection field

**Fig. 3:**
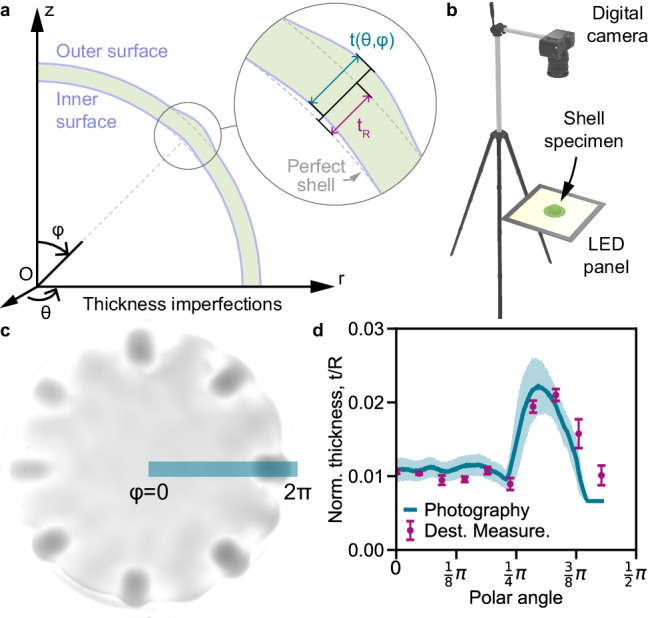
Quantification of vibration-induced imperfection as a measure of shell thickness. **a** Schematic of thickness measurement per spherical coordinate. **b** Photographic stage used to infer the shell’s thickness field. **c** Processed photograph of an imperfect shell featuring eight bump-like defects. **d** Comparison between inferred thickness from photography and destructive measurements of the same shell. Source data are provided as a Source data file.

To quantify the geometry of the imperfect shells fabricated using the above method, we reconstruct their thickness profile using optical photography. We observe that the vibration-generated imperfection generally takes the form of thickness variations across the surface of the shell (Fig. 3a). Further, deviations predominately occur on the outer surface of the shell; the inner surface remains spherical. We quantify the thickness of an imperfect shell at every spherical coordinate as *t*(*θ*, *ϕ*) = *d*_outer_(*θ*, *ϕ*) − *d*_inner_(*θ*, *ϕ*) where *d*_outer_ and *d*_inner_ are the distances from the outer and inner surfaces of the shell to the origin, respectively.

We simplify the process using optical photography as a measuring tool. We position each fabricated shell atop an LED light panel with constant ambient lighting. A digital camera (Nikon D780, 105 mm f/2.8G) is placed vertically above the shell at distance of 400 mm (Fig. 3b). Each resulting image is processed to remove optical distortion and then stereographically mapped to 2D (Fig. 3c)^37^. The degree of light penetration through the silicone serves as an indirect measure of thickness. With the exposure set to ensure no highlight or shadow clipping, i.e., no pure white or black, we are able to fit the relationship between intensity and thickness with an attenuation coefficient^38^.

To quantify the entire thickness field (see SI Sec. 2.2 for the image processing algorithm), we provide independent measures of the maximum and minimum thicknesses (${t}_{\max },{t}_{\min }$). This is achieved by excising segments of the fabricated shell and directly measuring their thickness using an optical microscope (Fig. 3d, see SI Sec. 2.1 for the destructive measurement method). These findings confirm that the vibration introduces repeatable, spatially structured imperfections whose geometry closely resembles vibrational mode shapes. See SI Fig. 2 for a library of different imperfection patterns.

Note that even in the absence of vibration, static casting of thin shells can produce axisymmetric thickness variations along the polar angle due to gravitational drainage and surface tension effects. For the silicone used in this study (Elite Double 32), Lee et al.^17^ reported baseline thickness deviations of approximately ± 6.6% relative to the mean thickness. Previous studies have shown that such uncontrolled, axisymmetric thickness variations have a weak influence on the critical buckling pressure compared to intentionally introduced geometric imperfections^22^. More recent work further demonstrates that when multiple imperfections are present and sufficiently separated, the largest imperfection dominates the buckling response^25^.

To ensure that this baseline effect does not influence our conclusions, we fabricated additional near-perfect shells without acoustic excitation using the identical experimental configuration. Measurements confirm that the polar thickness variation in these static shells remains within the same range reported in the literature and does not introduce significant non-axisymmetric features (see SI Fig. 8).

To assess repeatability, we fabricated multiple shells under identical excitation conditions and quantified the similarity of the resulting imperfection fields. Three near-perfect shells and three shells with eight bump-like defects were fabricated using multiple molds and photographed under identical conditions. Pairwise Pearson correlation coefficients were computed between the grayscale thickness maps of all specimens (see SI Fig. 3). Within each group, the mean correlation coefficients are 0.936 for near-perfect shells and 0.638 for shells with eight defects, indicating consistent reproduction of the targeted pattern. In contrast, correlations across the two groups are negligible (≈ −0.12).

### Tuning imperfection geometry

**Fig. 4:**
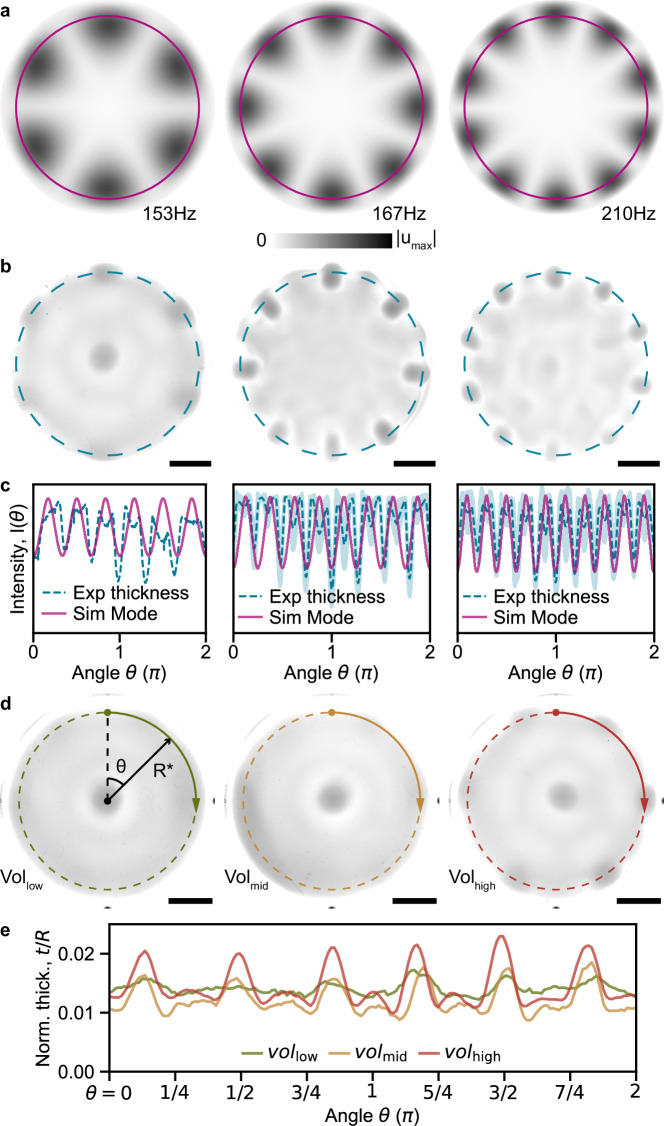
Comparisons between FE-predicted modes and the experimental imperfection patterns. **a** Simulated modal shapes for three distinct vibrational modes, showing radial deviation fields mapped onto 2D. **b** Photographs of the fabricated shells showing global shape deviations and mode symmetry. **c** Inferred intensity of the photographs as compared to the modal shape displacements, showing the correct number and distribution of peaks. **d** By varying the speaker volume and setting a constant frequency, we tune the amplitude of the thickness field *t*(*θ*, *ϕ*). **e** The thickness distribution at a single elevation is plotted for shells fabricated at three different volumes. All scale bars are 1 cm. Source data are provided as a Source data file.

We have shown earlier that the elastic mold vibrates in sync with the frequency of the speaker (Fig. 2a). This allows us to use FE simulations to predict and engineer the vibrational modes of the elastic mold as a function of the speaker frequency. Specifically, we calculate the natural vibrational modes and frequencies of the elastic mold with the equatorial degrees of freedom constrained. We select three different modal shapes for experimentation, namely *f* = 153, 167, and 210 Hz. These exhibit a single ring of six, eight, and ten antinodes, respectively. For each mode, we normalize the displacement field by its maximum absolute radial displacement, so that the largest deviation from the undeformed configuration equals one. We project the resulting displacement fields to 2D for visual comparison (Fig. 4a). See SI Sec. 3 for the detailed FE analysis protocol.

We then set the speaker to the corresponding frequencies and cast the shells using the aforementioned method. Although the FE vibrational mode shapes represent displacement fields of the mold and the experimentally measured shell geometries represent thickness variations, both are shown to share the same underlying spatial symmetry and antinode distribution (Fig. 4b). These observations support that vibrations can reliably embed modal features as geometric imperfections in thin shells. To quantitatively assess this correspondence, we compare the circumferential intensity patterns of the FE-predicted modes and the fabricated shells along a circular ring near the perimeter at *r* = 0.85*R*. For each specimen, the intensity is averaged along this ring and plotted as a function of azimuthal angle *θ*. The resulting profiles exhibit matching periodicity between simulation and experiment, with root-mean-square errors of 0.0798, 0.127, and 0.0924 for the 6-, 8-, and 10-defect cases, respectively. See Fig. 4c and SI Sec. 3.1 for details.

To further quantify periodicity, we compute the discrete Fourier transform of the azimuthal intensity profiles. The resulting power spectra show dominant peaks at harmonic indices corresponding to the number of antinodes in the targeted vibrational mode, both in the FE predictions and in the fabricated shells (SI Fig. 10d). Additional lower-amplitude harmonics reflect global asymmetry and nonlinearities at higher excitation amplitudes. A fully predictive description that couples acoustic excitation, structural vibration, oscillation-driven flow, and curing kinetics will be conducted in the future to quantitatively predict this correlation.

Next, we modulate the speaker volume, *v* = [55%, 69%, 72%], while keeping the frequency constant at *f* = 153 Hz. The resulting shells exhibit the same imperfection patterns qualitatively, however, the amplitude of thickness variation increases systematically (Fig. 4d). Plotting the thickness distribution along a single latitude shows six peaks in all three fabricated shells. The thickness profiles exhibit smooth, spatially graded variations that decay away from the antinodal peaks. This graded character is consistent with the spatial envelope of the FE-predicted modal displacement field. Their amplitude increases systematically as a function of the speaker volume.

By modulating both frequency and volume, we are able to systematically change imperfection geometry and severity. In principle, we have access to all modes as predicted by FE; however, experimentally, physically achievable eigenmodes are governed by several coupled experimental factors. First, the subwoofer used in this study accurately reproduces low-frequency excitation (40–250 Hz), while higher-order modes would require a mid-range or tweeter-type driver. Second, because the silicone mold is viscoelastic, maintaining the same vibration amplitude at higher frequencies would incur greater strain energy, which requires greater input power. If the speaker output remains constant, the amplitude decreases with frequency until the vibration becomes insufficient to influence the liquid layer. Finally, the secondary streaming flow that drives material redistribution penetrates a depth proportional to the vibrational wavelength. As the distance between neighboring antinodes decreases for higher modes, this flow becomes confined near the surface of the silicone mold and its magnitude diminishes.

### Mechanical response of imperfect shells

**Fig. 5:**
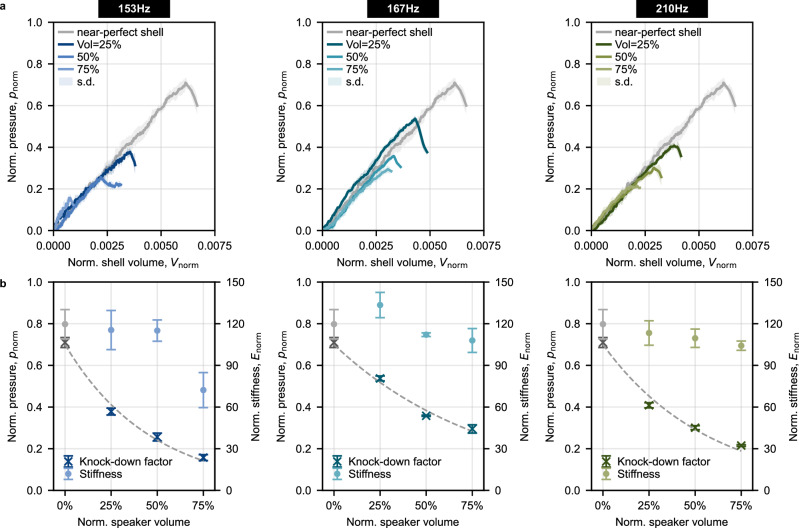
The buckling response of hemispherical shells with vibration-induced imperfections. **a** Normalized pressure–volume curves for shells fabricated with specific speaker excitation volume and frequencies. The behavior of a shell with no vibration-induced imperfections is plotted for reference. **b** Normalized pre-buckling stiffness *E*_norm_ and knockdown factors as a function of speaker volume, quantifying the reduction in structural stability with increasing imperfection severity. Source data are provided as a Source data file.

We quantify the mechanical response of this class of imperfect shells under increasing external pressure, achieved by progressively reducing the internal pressure of the shell (Fig. 5). In addition to perfect shells, a total of nine different imperfect hemispherical shells are fabricated and divided into three groups, each subjected to acoustic excitation at a fixed frequency listed in Fig. 4a. Within each group, the shells are exposed to varying excitation amplitudes by adjusting the speaker’s output volume. Quasi-static under-pressure experiments are performed on the shells. The interior of each shell is connected to an automated syringe pump via a flexible tube. The volume in the shell *V*_shell_, the tube *V*_tube_ and the 1 mL syringe *V*_syr_ form a closed system with a total volume *V*_total_. A pressure sensor is used to record pressure as the syringe draws air and expands the total volume (see SI Sec. 4.1 for detailed experimental setup). As the total volume increases, the pressure decreases until the shell buckles, at which point the volume decreases abruptly and the pressure rebounds.

Assuming the ideal gas law holds, we can isolate the volume under the shell (see SI Sec. 4.2 for calculation). This allows us to define a normalized volume *V*_norm_ as Δ*V*/*V*_shell_, where $\Delta V={V}_{{{{\rm{shell}}}}}-{{V}_{{{{\rm{shell}}}}}}^{{{{\rm{init}}}}}$. We further define a normalized internal pressure as *p*_norm_ = *p*/*p*_crit_, where *p*_crit_ is the under-pressure required to buckle a perfect hemispherical shell. We proceed to plot the normalized internal pressure as a function of normalized volume change (Fig. 5a). Each *PV* curve corresponds to a specific excitation volume and frequency. Increasing the speaker volume results in a systematic reduction in critical buckling pressure and earlier onset of instability.

We further quantify the stiffness as the slope of the quasi-linear portion of the normalized pressure–volume curves prior to the onset of instability (Fig. 5b). We compute a normalized stiffness as *E*_norm_ = *p*_norm_/*V*_norm_ over the initial linear regime (*V*_norm_ = 0.002). Across all excitation frequencies and volumes, the measured stiffness values remain within the experimental variability of the near-perfect shell, indicating that the imposed imperfections primarily affect the critical buckling pressure rather than the pre-buckling stiffness. A deviation is observed only for shells with six defects fabricated at the highest excitation volume, where large thickness variations likely violate the assumptions of thin-shell behavior. See SI Table 1 for numerical values.

We then calculate the corresponding knockdown factors *κ* of each shell as the ratio between the maximum buckling pressure of imperfect shells and that of the theoretical prediction of a perfect shell (see SI Sec. 4.3 for characterization of a perfect shell). These are plotted against speaker volume to confirm a consistent decline in structural capacity with stronger perturbation volume (Fig. 5b). To further study the relationship between excitation amplitude and structural capacity, we fit exponential decay curves of the form $\kappa=\alpha \exp (-k\cdot v)$ to the measured knockdown factors, where *v* is the normalized speaker volume. Across all three modal families, the data are well described by a shared pre-factor *α* = 0.7, with mode-dependent decay constants *k*_6_ = 0.532, *k*_8_ = 0.301, and *k*_10_ = 0.437 (*R*^2^ = 0.991, 0.988, 0.980, respectively). The shared pre-factor suggests that the baseline knockdown factor at low excitation is mode-independent, while the differing decay constants reflect the mode-dependent sensitivity of the buckling response to increasing imperfection severity. These fits are shown in Fig. 5b.

## Discussion

We have introduced a vibration-assisted fabrication method to create hemispherical shells with spatially distributed, mode-shaped geometric imperfections in the form of thickness variations. By casting liquid silicone atop a vibrating elastic mold, we exploit vibration-driven flow and secondary streaming to imprint modal shapes directly onto the shell’s thickness field. The resulting imperfections are tunable in geometry via excitation frequency and scalable in severity through speaker volume, enabling controlled access to classes of imperfection patterns that are difficult to realize using conventional fabrication approaches.

To explore the underlying mechanism, we address the following physical processes. Analysis of the vibration-driven fluid motion reveals a secondary streaming flow that drives material accumulation at regions of large vibration amplitude. Quantitative correlation analysis between the measured imperfection fields and FE-predicted vibrational modes demonstrates that the vibration-assisted patterning achieves high spatial fidelity and repeatability across shells fabricated under identical conditions. Mechanical buckling experiments demonstrate a systematic reduction in structural capacity with increasing imperfection amplitude.

Beyond single-mode excitation, this fabrication method enables the generation of mixed-mode and non-periodic imperfection fields through broadband excitation. By exciting the mold with broadband signals, the resulting thickness variations reflect both superposed and emerging modal features. The resulting shell geometries exhibit features both resembling the single-mode patterns shown in Fig. 4 and previously unobserved spatial patterns. Representative examples and experimental details are provided in the SI Sec. 1.6. The ability to imprint complex thickness landscapes highlights the flexibility of vibration-assisted patterning and suggests routes toward exploring the mechanics of imperfection interactions in thin shells.

**Fig. 6:**
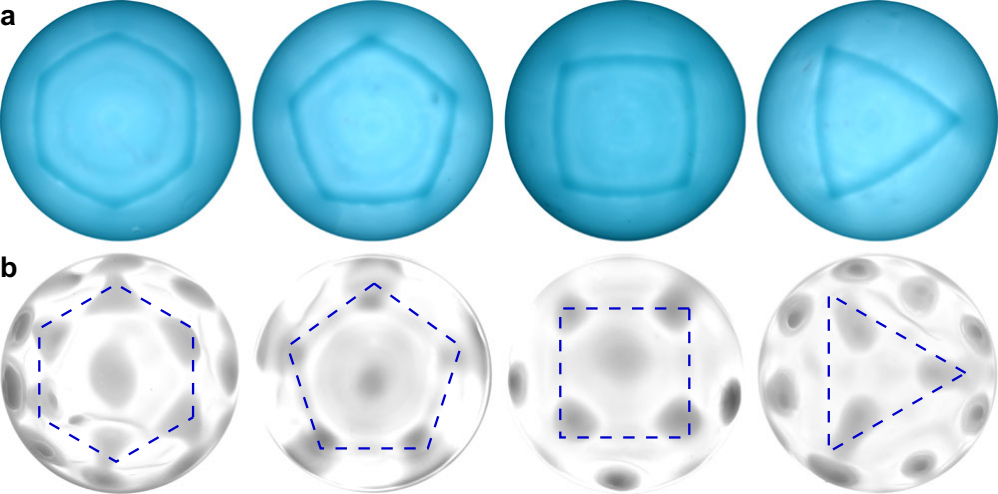
Imperfection shapes produced by altering the shape of the elastic hemispherical mold. **a** Protrusions of different shapes, including a hexagon, pentagon, square, and triangle, are embedded onto the mold. **b** The resulting imperfection shapes show silicone accumulating at the vertices of the mold polygons.

Furthermore, by pre-designing the mold geometry with polygonal features (i.e., triangular, square, pentagonal and hexagonal protrusions), the vibrational response of the mold can be designed to produce non-standard imperfection patterns, including configurations with odd numbers of bump-like defects (see Fig. 6 and SI Sec. 1.7). This demonstrates that combining mold geometry design with structural vibration significantly broadens the accessible design space of imperfection geometries.

While the present work focuses on hemispherical shells as a canonical system for imperfection-sensitive buckling, the underlying fabrication method is readily extendable to programming the mechanics of other soft material systems^39,40^. Here, we reinterpret thickness imperfections as architected features, and leverage the spatially distributed nature of such features as a design variable. Such features may be used in localizing compliance, biasing snap-through behavior, or encoding preferred post-buckling modes^41,42^.

In programmable materials and soft robotics, geometric instabilities are increasingly exploited to achieve large, rapid shape changes, mechanical logic, or passive actuation without embedded electronics^43–45^. Examples include morphable shells that snap between configurations, soft robotic grippers that close via elastic instability, and surfaces that reversibly change curvature under pressure^46–48^. The ability to imprint global, mode-shaped thickness fields provides a means to pre-program these behaviors directly into the material geometry, rather than relying on external constraints or active control. In particular, recent studies have shown that spatial disorder itself can be treated as a programmable design variable, enabling tunable mechanical response and emergent behavior in elastic systems^49–51^.

In conclusion, this study establishes vibration-assisted fabrication as an enabling platform for experimentally accessing distributed, tunable geometric features that reflect the complex defect landscapes encountered in real-world structures.

## Methods

### Fabrication

Elastic hemispherical molds are fabricated by repeatedly coating a precision-machined metal sphere (*D* = 50.8 mm) with a platinum-catalyzed silicone rubber (Mold Star 16 Fast, Smooth-On; PSR16), adding ≈ 0.35 mm per cure cycle. The Young’s modulus of PSR16 (*E* = 0.85 MPa) is measured by tensile testing of ASTM D412 dogbones. To cast imperfect shells, the mold is mounted on a PMMA plate bolted to the rim of an acoustic subwoofer (Polk Audio PSW10), sprayed with a mold-release (Ease Release 200), and coated with a two-part vinyl polysiloxane (Elite Double 32, Zhermack; VPS32) while the speaker is driven at a prescribed frequency and volume. Mold vibration redistributes the liquid silicone into a steady thickness pattern that is fixed in place over the ~ 20 min cure cycle. Imperfection geometry is set by the excitation frequency (chosen to coincide with a computed modal frequency of the mold; see below) and amplitude by the speaker volume. Mixed-mode patterns are generated using broadband audio signals, and non-standard patterns are produced by casting the mold against 3D-printed hemispherical negatives containing polygonal indentations. Full protocols, including material characterization and flow visualization (laser-sheet node/antinode identification, stroboscopic imaging, and reflective-particle tracking), are given in SI Sec. 1.

### Thickness measurement

Ground-truth thickness profiles are obtained destructively by excising strip segments and measuring them under an optical microscope. For non-destructive reconstruction, each shell is photographed from vertically above atop an LED light panel using a DSLR camera (Nikon D780, Nikkor 105 mm f/2.8G) under controlled lighting. Grayscale images are corrected for geometric edge-darkening and inverse-orthographically remapped, and local thickness is inferred via the Beer-Lambert law with the attenuation coefficient and incident intensity fit against the destructive measurements. Repeatability is assessed by Pearson correlation of grayscale thickness maps across independently fabricated specimens. Full calibration details are provided in SI Sec. 2.

### Finite element modal analysis

Modal analysis of the elastic mold is performed in Abaqus/Standard using reduced-integration shell elements (S4R) on a perfect hemisphere (*R* = 25.4 mm) with the equator fully constrained and the measured PSR16 properties (*E* = 0.85 MPa, *ν* = 0.5). The first 30 eigenmodes are extracted using the *Lanczos* solver. Modes with 6, 8, and 10 circumferential antinodes (*f* = 153, 167, and 210 Hz) are selected for fabrication. FE-predicted modes and fabricated shells are compared via root-mean-square error and discrete Fourier decomposition of circumferential intensity profiles at *r* = 0.85*R*. See SI Sec. 3.

### Mechanical buckling experiments

Quasi-static under-pressure buckling tests are performed in a closed-volume pneumatic setup comprising the air beneath the shell, flexible tubing, and an automated 1 mL syringe pump, sealed between two clamped PMMA plates. Internal pressure is monitored with an MPL3115A2 barometric sensor (ELEGOO UNO R3 microcontroller, 0.8 s sampling). The syringe slowly withdraws air until the shell buckles; the volume beneath the shell at each timepoint is recovered from the measured pressure via the ideal gas law. Pressure is normalized by the classical critical pressure ${p}_{{{{\rm{crit}}}}}=2E/\sqrt{3(1-{\nu }^{2})}\cdot {(t/R)}^{2}=102.4\,\,{\mbox{Pa}}$ for our VPS32 shells (*E* = 1.2 MPa, *ν* = 0.5, *t* = 0.2 mm, *R* = 25.4 mm), and volume by the initial shell volume. Pre-buckling stiffness is computed as the slope of the normalized pressure–volume curve over the initial linear regime. Knockdown factors are fit to $\kappa=\alpha \exp (-kv)$ by nonlinear least-squares regression. Three shells are tested per excitation condition. See SI Sec. 4.

### Weakly nonlinear analysis of the vibration-induced flow

To explain why liquid silicone accumulates at the mold’s antinodes with a free-surface wavenumber twice that of the mold’s vibration, we perform a weakly nonlinear analysis of a Newtonian fluid layer driven by a prescribed standing-wave deflection of the lower boundary. Expanding the velocity, pressure, and free-surface fields in powers of the vibration amplitude, the order-ϵ^*^ primary flow is linear and oscillatory, while the order-ϵ^*2^ secondary flow is driven by Reynolds stresses and a boundary forcing with wavenumber 2k^*^. Symmetry arguments fix the spatial phase, yielding a streaming flow with pressure maxima above the antinodes that deflects the free surface outward there. In the experimental parameter regime ($Re={{{\mathcal{O}}}}(1{0}^{-2})$, $Ca={{{\mathcal{O}}}}(1{0}^{2})$, and ${k}^{*}={{{\mathcal{O}}}}(1{0}^{-1})$), the steady free-surface amplitude scales as *A* ~ *λ*^2^*μ*^2^*ω*^2^*ϵ*^2^*γ*^−2^*H*^−1^. The full derivation is provided in SI Sec. 5.

## Supplementary information

Supplementary Information

Description of Additional Supplementary Files

Supplementary Movie 1

Supplementary Movie 2

Supplementary Movie 3

Transparent Peer Review file

## Source data

Source Data

## Data Availability

The source data underlying all figures presented in this study are provided with this paper as a Source data file. The complete dataset has been deposited in the Zenodo public repository and is available at 10.5281/zenodo.19682356^52^. No datasets with restricted access are associated with this study. Source data are provided with this paper.

## Code availability

All custom scripts for figure generation are deposited in the same Zenodo repository (10.5281/zenodo.19682356)^52^. Scripts are developed and tested with Python 3.10 (NumPy, pandas, SciPy, Matplotlib, OpenCV).
