# Supplementary material for: Vibration-assisted fabrication of thin shells with spatially distributed imperfections
Source: Nat Commun. 2026 May 20;17:7324. doi: 10.1038/s41467-026-73343-2 (PMC13402623; doi:10.1038/s41467-026-73343-2)
Supplement: Supplementary file 1 — Supplementary Information [file 41467_2026_73343_MOESM1_ESM.pdf]

Supplementary Information

Vibration-Assisted Fabrication of Thin Shells with Spatially Distributed Imperfections

Ilyes Krida[1], Jacob Tang[2], Leo Mangalath[1], Daniel Floryan[1], and Tian Chen[1,3*]

[1]Department of Mechanical & Aerospace Engineering, University of Houston, TX, United States of America
[2]Department of Aerospace & Mechanical Engineering, University of Southern California, CA, United States of America
[3]Department of Aeronautics, Imperial College London, England, United Kingdom
[*]Corresponding author: tian.chen@imperial.ac.uk

Contents

1 Fabrication protocol **2**
1.1 Material characterization . . . 2
1.2 Fabrication of elastic hemispherical molds . . . 2
1.3 Fabrication of imperfect hemispherical shells with acoustic excitation . . . 2
1.4 Different imperfect shells with varying speaker frequency and volume . . . 3
1.5 Robustness and repeatability . . . 4
1.6 Mixed-mode imperfections . . . 4
1.7 Imperfection geometries through mold design . . . 4

2 Measuring fabricated shell thickness profile **7**
2.1 Destructive method . . . 7
2.2 Photographic method . . . 7
2.3 Calibration photographs and inference of thickness . . . 8

3 Finite-element modal analysis **9**
3.1 Inference of shell thickness from FE analysis . . . 10

4 Mechanical characterization of hemispherical shells **12**
4.1 Experimental setup . . . 12
4.2 Calculation of shell volume . . . 12
4.3 Calculation of critical pressure of a perfect shell . . . 13
4.4 Stiffness measures . . . 13

5 Analysis of fluid motion **14**

6 Supplementary Movies **18**

1 Fabrication protocol

1.1 Material characterization

Two materials are used in this study. Mold Star 16 Fast Platinum Silicone Rubber (PSR16) for fabrication of the mold, and Zhermack Elite 32 (VPS32) for the fabrication of the imperfect shells. To predict the vibrational characteristics of the mold using Finite Element (FE), we conduct material characterization of the PSR16 material. We cast dogbones according to ASTM D412, type D geometry, and subject them to tension up to a strain of 0.02 at a displacement rate of $0.15\,\mathrm{mm\,s^{-1}}$. We capture the initial portion of the stress-strain curve (S.Fig. 1a) and calculate the Young's modulus of PSR16 to be $E = 0.85\,\mathrm{MPa}$.

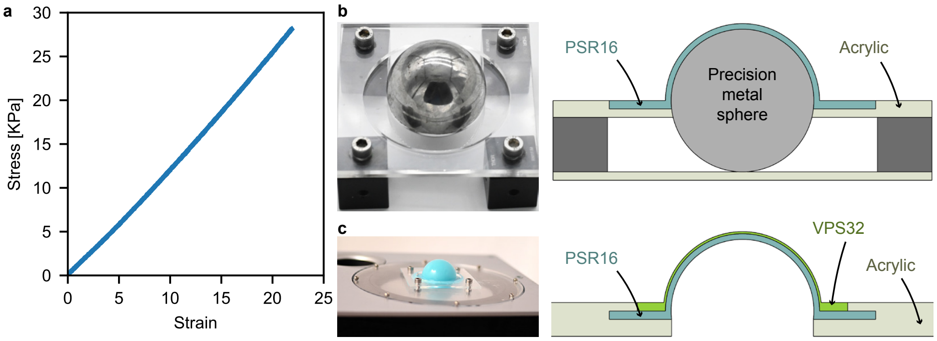

Supplementary Figure 1: **Fabrication of the mold and of the imperfect shells.** a. Stress-strain plot of the tensile test of mold material PSR16. b. Physical setup of the mold fabrication. Cross section showing the cavity upon which PSR16 is poured. c. Physical setup of the acoustic-assisted fabrication of the imperfect shells. Cross sectional schematic showing where the VPS32 is poured. Source data are provided as a Source Data file.

1.2 Fabrication of elastic hemispherical molds

The elastic hemispherical mold used in the acoustic casting is fabricated by repeatedly coating a precision-machined metal sphere (diameter $D = 50.8\,\mathrm{mm}$) with a platinum-catalyzed silicone rubber (Mold Star 16 Fast, Smooth-On). The setup positions the metal sphere using a PMMA sheet (S.Fig. 1). To prepare the mixture for pouring, we use a centrifugal mixer (ARE-250, Thinky Corporation) to homogenize the base and catalyst. The two-part mixtures of PSR16 are prepared at a 1:1 weight ratio of base to catalyst. Each mixture is mixed for 30 seconds at 2000 rpm in the clockwise direction, followed by another 30 seconds at 2000 rpm counterclockwise. Each layer is poured and allowed to fully cure for 20 minutes at room temperature before the next is added. Each curing cycle adds approximately 0.35 mm to the mold thickness.

1.3 Fabrication of imperfect hemispherical shells with acoustic excitation

Once the mold is fabricated, it is affixed using a PMMA sheet to an acoustic subwoofer (Polk Audio PSW10 10" Powered Subwoofer). The PMMA sheet clamps around the PSR16 elastic mold and has a circular cut out matching the diameter of the mold underneath (S.Fig. 1b).

Prior to each casting, the mold is sprayed with an aerosol-based mold release (Mann Release Technologies, Ease Release 200). Silicone (Zhermack Elite 32) prepared with the same procedure as above is then poured over the PSR16 elastic mold while the speaker is turned on at a prescribed

frequency and volume. This continues until the silicone fully cures after 20 minutes, and the imperfect shell is peeled off the elastic mold.

1.4 Different imperfect shells with varying speaker frequency and volume

By changing the frequency of the acoustic speaker, we can excite different vibrational modes of the mold. Conversely, by modulating the speaker volume, we can change the amplitude of that vibration. S.Figure 2 shows photographically the effect of the different frequencies and volumes on the eventual shape of the imperfection.

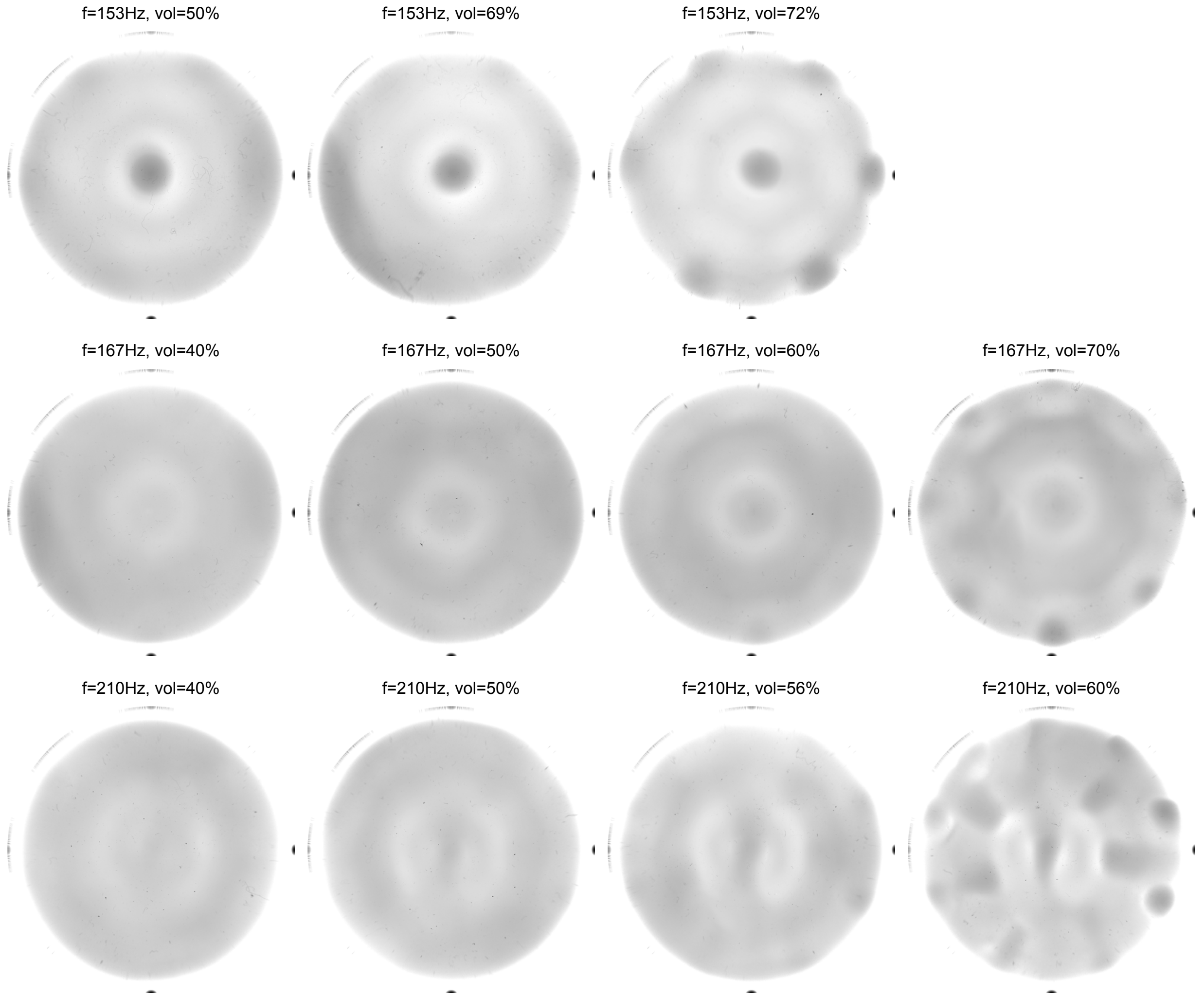

Supplementary Figure 2: **Top and side views of the elastic hemispherical shell responding to acoustic excitation at different frequencies and speaker volumes.** Each column shows the shell's shape under a specific excitation condition, with the frequency and volume noted above. The top view of each specimen shows the vibrational mode patterns, while the bottom image shows the same shell from the side.

1.5 Robustness and repeatability

To demonstrate the robustness and repeatability of the fabrication method, we fabricate three near-perfect and three shells with 8 bump-like defects, and calculate the Pearson correlation coefficient within each group. Note that since the lighting condition of the photographs are identical, the intensity of the pixels of the gray-scale images are compared directly. Each pair of images are treated as vectors of pixel intensities. Each image is first converted to a one-dimensional vector by stacking all pixels, and its mean intensity is subtracted to remove global brightness offsets. The Pearson correlation coefficient p is then calculated as the normalized inner product of the mean-centered vectors, obtained by dividing their dot product by the product of their Euclidean norms (Eq. 1),

$$p(I_1, I_2) = \frac{\sum_{i=1}^{N}(I_{1,i} - \bar{I}_1)(I_{2,i} - \bar{I}_2)}{\sqrt{\sum_{i=1}^{N}(I_{1,i} - \bar{I}_1)^2}\sqrt{\sum_{i=1}^{N}(I_{2,i} - \bar{I}_2)^2}} \tag{1}$$

, where I_1 and I_2 are the vectors of pixel intensities of two images being correlated, and N is the number of pixels within the circular mask of the photographs. This normalization renders the metric invariant to global intensity scaling and offset.

The correlation between three near-perfect shells and three shells with 8 bump-like defects are calculated based on Eq. 1 (S.Fig. 3). Within each group, the correlation means are $p_0 = 0.936$ and $p_8 = 0.638$ for near-perfect and 8 defects respectively, with $p = 1$ representing a perfect correlation. The deviations within each group can be attributed to three primary causes. The first is the time lapsed between silicone mixing and pouring. This delay influences the eventual thickness of the specimen [1] and may explain the difference between t8-s3 and the remaining two. The second is the imprecision in positioning and leveling the mold on the speaker. The mold is secured onto a circular PMMA plate, which is bolted to the rim of the loudspeaker. In this setup, the mold may not be perfectly concentric to the diaphragm of the speaker. Depending on the tightness of the bolts, the lip of the mold may be asymmetrically compressed. Both errors may result in the specimen appearing asymmetric. The third is in image processing, specifically, we manually align one bump-like defect along the horizontal axis by rotating the image. Nevertheless, the within-group correlations are substantially higher than cross-group correlations ($p_{0-8} = -0.117$), confirming that the fabrication process produces consistent imperfection patterns.

1.6 Mixed-mode imperfections

To demonstrate the ability of the fabrication method to generate mixed-mode and non-periodic imperfection fields, we fabricated additional shells under broadband and time-varying excitation conditions. Rather than applying single-frequency sinusoidal signals, the mold is driven using audio signals containing a wide range of frequencies and temporal modulations. This approach results in highly diverse, non-axisymmetric thickness patterns that differ qualitatively from the single-mode cases presented in the main text.

Representative examples include excitation using music spanning different genres and spectral contents, which serve here as convenient broadband inputs. The resulting imperfection fields exhibit both features resembling individual modal patterns and previously unobserved spatial organizations (S.Fig. 4).

1.7 Imperfection geometries through mold design

To demonstrate that the vibration-assisted fabrication method is not limited to standard modal shapes, we fabricate elastic molds with pre-designed geometric features. Rather than casting

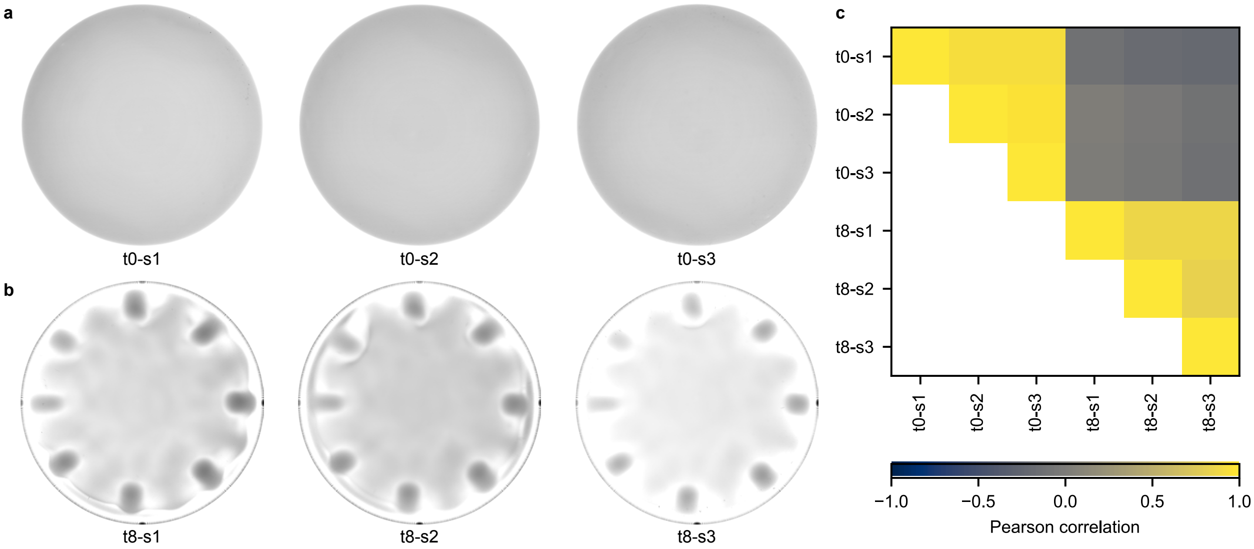

Supplementary Figure 3: **Shape comparison between shells based on photographic evidence.** a. Three near-perfect shells and b. three shells with 8 bump-like defects are compared. c. The resulting Pearson correlation is plotted showing close correlation between shells within each group, and minimal correlation across groups. Source data are provided as a Source Data file.

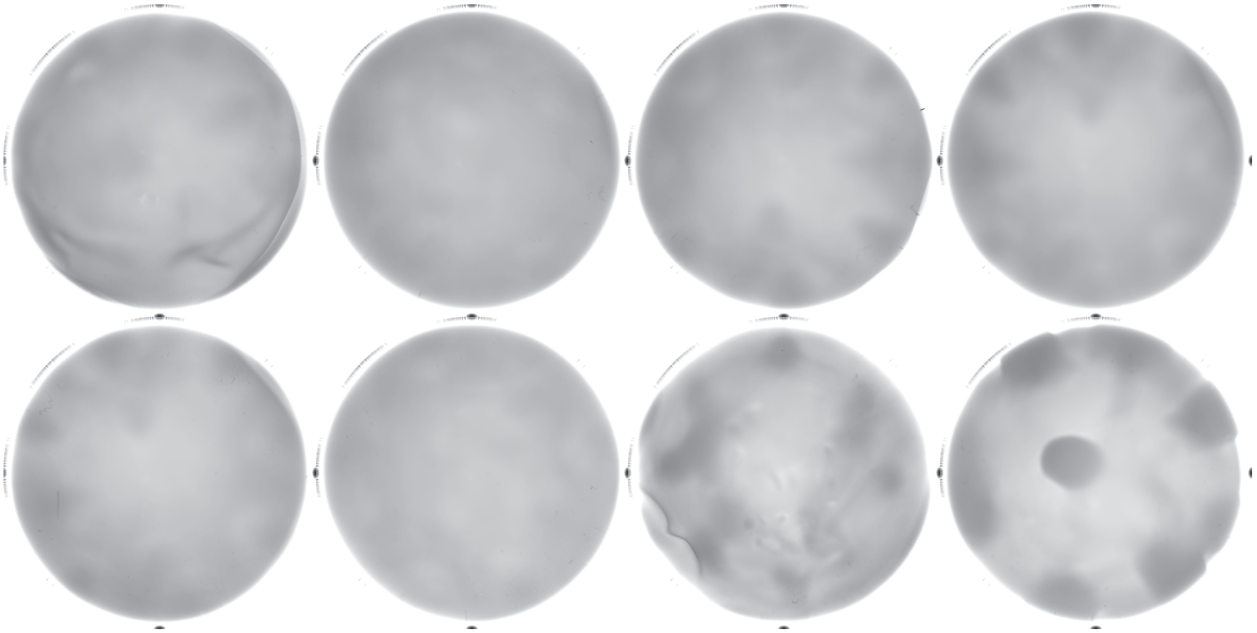

Supplementary Figure 4: **Mixed-mode imperfections.** Eight different shells fabricated using sequential broadband and time-varying excitation signals.

the elastic molds through repeated coating of a smooth metal sphere, we 3D print hemispherical negatives containing polygonal indentations (triangular, square, pentagonal and hexagonal, see S.Fig. 5a,b). These negatives are used to fabricate elastic molds with corresponding localized

thickness increase on their underside (S.Fig. 5c). The rest of the fabrication procedure remains identical: the mold is mounted onto the speaker, and silicone is poured as the mold is excited at a single frequency. The resulting cast shells (S.Fig. 5d) demonstrate that material accumulation concentrates at the antinodes of the modified molds, which coincide with the vertices of the polygonal features. This produces imperfection patterns that break the symmetry of standard vibrational modes, including configurations with odd numbers of bump-like defects that are not achievable with the hemispherical mold.

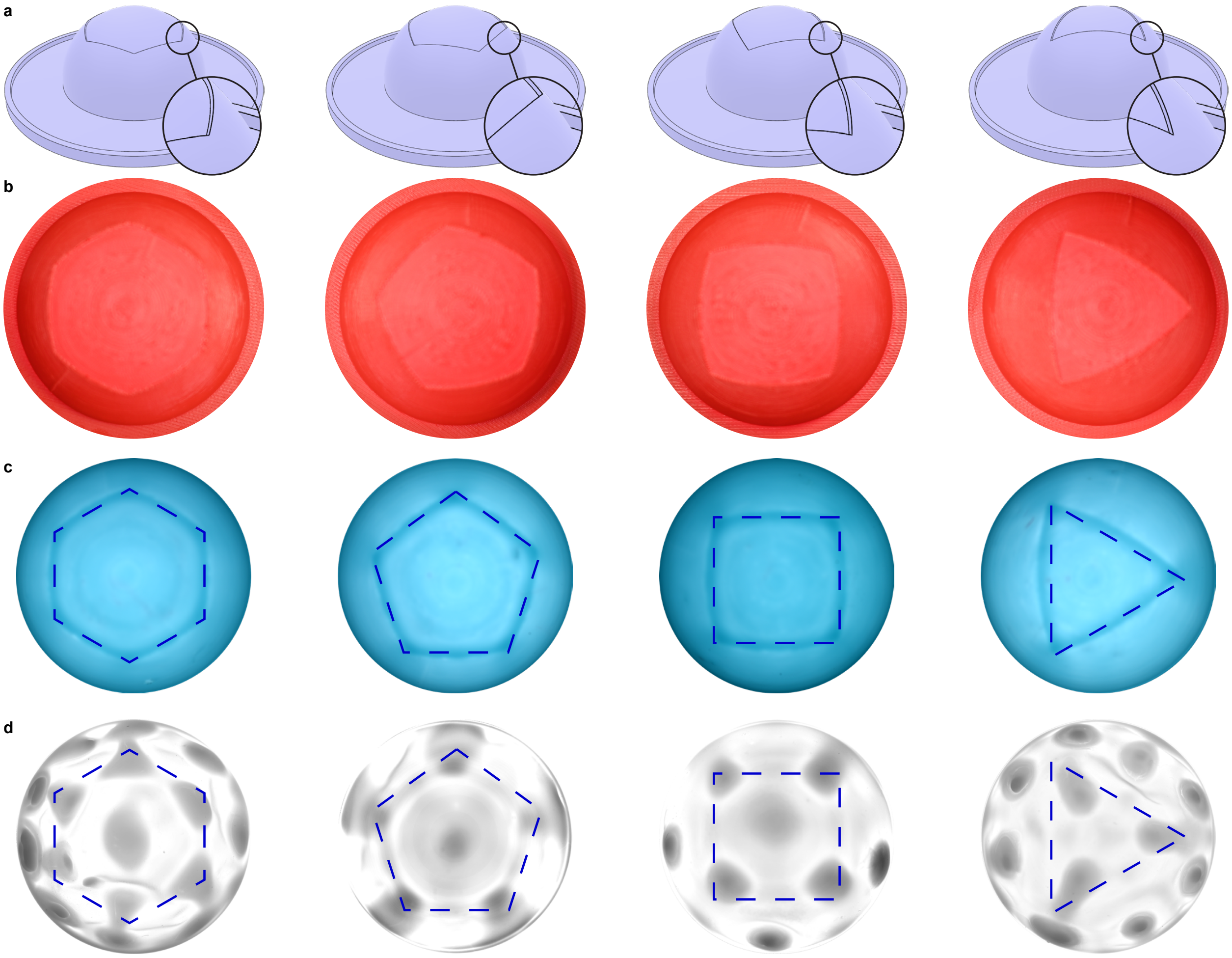

Supplementary Figure 5: **Casting using elastic molds with polygonal features.** a,b. Design and 3D printed hemispherical negatives with triangular, square, pentagonal and hexagonal indentations of 0.5 mm. c. Elastic molds where thickness is locally increased from the underside. d. The resulting cast shells showing that material accumulation is governed by the vertices of the polygonal features.

2 Measuring fabricated shell thickness profile

2.1 Destructive method

First we discuss the use of destructive measurement methods to capture the thickness profile of fabricated shells. To accurately measure the thickness variations in the shells, we extract strip segments (S.Fig. 6a) and measure the cross section thickness as a function of the polar angle using an optical microscope. We conduct this with three near-perfect shells fabricated without acoustic excitation (*i.e.*, static casts) but otherwise using the identical setup as our subsequent fabrication protocols (*i.e.*, thick elastic hemispherical mold atop the speaker). The same destructive measurement method is applied to specimens featuring 8 visible bump-like defects. These measurements serve as the ground-truth to correlate pixel intensity in photographs with thickness values.

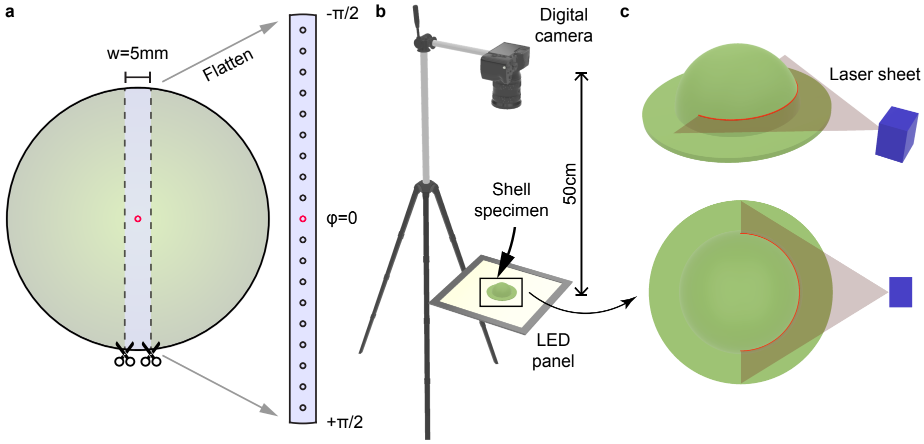

Supplementary Figure 6: **Destructive and non-destructive means of shell thickness measurement.** a. The segment extracted from the near-perfect shells. The segment is flattened and its thickness is measured from end to end, covering polar angles from $\phi = [-\pi/2, \pi/2]$. b. Photography setup to capture shell thickness profile. c. Schematic setup of laser sheet positioning in relation to the hemipsherical shells.

2.2 Photographic method

Next we inferred the shells' thickness profiles non-destructively through photography. Here, we ensure that the photography conditions including lighting, placement of specimens, and camera setup remain identical across all photos (S.Fig. 6b). With photography, each fabricated shell, both near-perfect and with-defects, is placed atop an LED panel (HSK A4 LED Light Box) in an otherwise dark room and photographed using a DSLR camera (Nikon D780) from the top at a fixed distance (0.5 meters) using a prime lens (Nikkor 105mm f/2.8G). From the resulting photos, we note that they are largely monochromatic, where there is limited Hue (H) and Saturation (S) distribution (see S.Fig. 7). This allows us to convert the Value (V) to greyscale, where each pixel has an intensity value I, without ambiguity. We then proceed to calibrate the images, and construct a correlation between the thickness profile of the shell and the greyscale value of the image ($0 \leq I \leq 1$ where 0 is black and 1 is white).

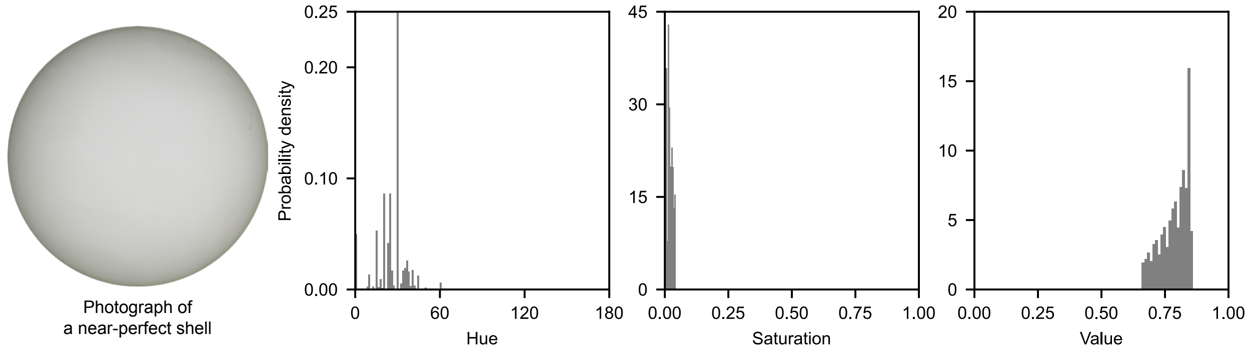

Supplementary Figure 7: **Analysis of a photographed shell showing the distribution of Hue, Saturation and Value.** The narrow distribution of Hue and Saturation allows the image to be converted to greyscale with minimal ambiguity. Source data are provided as a Source Data file.

2.3 Calibration photographs and inference of thickness

For calibration, we observe that the photographs of near-perfect shells show a progressive darkening towards the edge (see S.Fig. 8a). In physical measurements as well as in literature [1], this trend is much less pronounced for the silicone we used (Zhermack Elite Double Fast, Double 32). Rather, this optical effect arises from geometry rather than material variation. Under illumination, rays passing through the shell at the pole intersect the surface approximately orthogonally, whereas rays near the periphery traverse the shell at increasingly oblique angles. As a result, the optical path length through the material increases approximately as $1/\cos\phi$ where ϕ is the polar angle. Because transmitted intensity decays exponentially with optical path length according to Beer–Lambert attenuation, this geometric increase in path length leads to systematic edge darkening. We define a multiplicative correction filter F such that the corrected intensity $I_{\mathrm{c}}(\phi)$ equals to $I_{\mathrm{original}}(\phi) \cdot \xi(\phi)$, where

$$\xi(\phi) = \exp\left[K\left(\frac{1}{\cos\phi} - 1\right)\right]. \tag{2}$$

The correction strength factor K is chosen to empirically fit the photographed thickness of the near-perfect shells to the direct microscopy measurements and remains constant across all images (see S.Fig. 8b).

Next, the photographs are inverse-orthographically remapped to remove distortion arising from projecting a hemisphere (of the shell) to the flat plane of the camera sensor (Fig.8c). Under orthographical mapping, the pixels are expanded around the equator and/or compressed near the zenith in the radial direction as a function of the polar angle. To correct for this, the new radial coordinate of the inverse mapping equals to

$$r_{\mathrm{new}} = 2\frac{R}{\pi}\arcsin\frac{r}{R}, \tag{3}$$

where R is the radius of the hemisphere.

Finally, to correlate thickness, t, with respect to light intensity, we refer again to the Beer–Lambert law for a uniform material, $I \approx I_0 \exp -\mu t$, where with μ is the attenuation coefficient, I is the

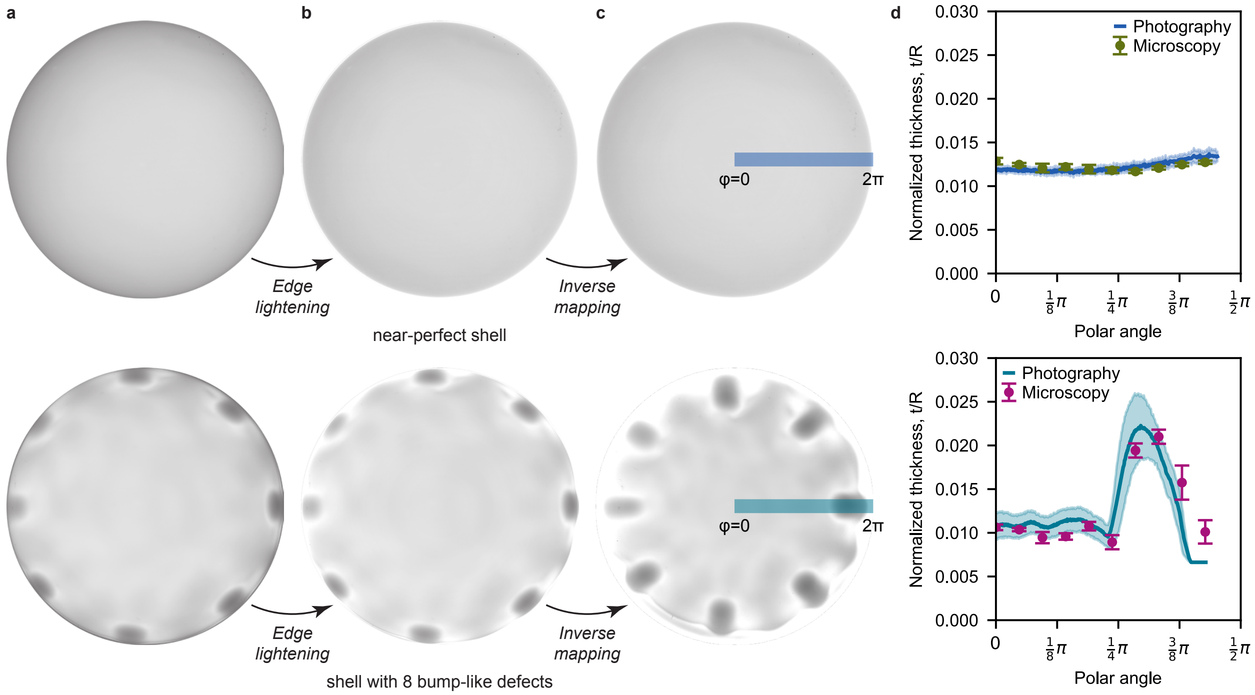

Supplementary Figure 8: **Processing of photographed shells, both near-perfect and with defects.** a. Greyscale version of the photographs, b. corrected for edge darkening, c. inverse orthographic remapping and d. thickness profile along one azimuthal angle across polar angles from 0 to $\pi/2$ as directly measured and as inferred through photography. Source data are provided as a Source Data file.

intensity and I_0 is the incident intensity. Rearranging to isolate the thickness,

$$t \approx -\frac{1}{\mu} \log \frac{I}{I_0}, \tag{4}$$

and we fit the attenuation coefficient and the incident intensity I_0 to the measurements of the shells discussed above. Specifically, they are empirically fitted to the destructive measurements of the imperfect shells, in particular, the ones with 8 bump-like defects. This is due to the fact that imperfect shells possess a larger range of thickness values whose extrema are thinner and thicker than the near-perfect shells at different points on the shell. As a result, the attenuation coefficient $\mu = 1.40\,\mathrm{mm}^{-1}$ and $I_0 = 1.264$. Using this, we plot the thickness profile along a single azimuthal angle picked to align with one of the bump-like defects, from zenith, $\phi = 0$, to the horizon, $\phi = \pi/2$, for near-perfect shells and for ones with 8 bump-like defects (S.Fig. 8d). The accuracy of the photography method allows us to rapidly measure the thickness of shells without further need of destructive methods or CT scanning.

3 Finite-element modal analysis

Finite element (FE) simulations are conducted using Abaqus to analyze the dynamical behavior of hemispherical molds. Specifically, the simulations are used to compute natural frequencies and mode shapes.

A geometrically perfect hemisphere is defined in spherical coordinates with radius $R = 25.4\,\text{mm}$, spanning the full hemisphere domain ($\theta \in [0, \pi/2]$,$\phi \in [0, 2\pi]$). The shell thickness is set to $t =$ 0.7 mm. The geometry is discretized using reduced-integration quadrilateral shell elements (S4R), with approximately *8100* elements used in total. The equatorial boundary is constrained in all degrees of freedom to emulate the experimental condition.

A linear perturbation step is used to extract the first 30 eigenmodes of the hemispherical shell. The *Lanczos* eigensolver is employed. The computed mode shapes characterize the shell's natural vibration patterns and are used as a basis for interpreting deformation patterns observed during acoustic excitation (S.Fig. 9).

3.1 Inference of shell thickness from FE analysis

Here, we measure the similarity between the photographs of the fabricated shells and the resulting shapes of the mold's normal mode. We elect to conduct linear frequency extraction analysis to demonstrate that the specimens can emulate the shape of vibrational modes of the elastic molds. Since all vibrations can be represented as a superposition of natural modes (exactly in the linear case, approximately otherwise), by showing correlation between the fabricated specimens and the FE modal shapes, we demonstrate the versatility of the fabrication method.

In Abaqus, the eigenvectors are normalized such that the maximum of each displacement component is 1. The magnitude of displacement is visualized using greyscale where white and black correspond to zero and maximum displacements respectively. As a result of the above analysis on the fluidic motion, this greyscale value is squared. The resulting images are scaled with the same number of pixels as the photographs of the fabricated shells. Note that we aim to correlate two different physical quantities, namely the modal displacements of the mold and the thickness of the shells fabricated using the mold. Therefore, rather than comparing the images on a pixel-level, we measure the similarities in the features of the images. Specifically, we assess whether the defects are present at the antinodes of the modal shape.

We proceed to extract three modal shapes at the requested frequencies; these feature 6, 8 and 10 bump-like defects respectively (S.Fig. 10a). The displacement fields resulting from FE modal analysis are plotted alongside the photographs of the imperfect shells (S.Fig. 10b). We plot the amplitudes of the intensity as a function of the azimuthal angle around a fixed radius ($0.85R$) to showcase the similarity between the two (S.Fig. 10c). The RMSE between experimental and

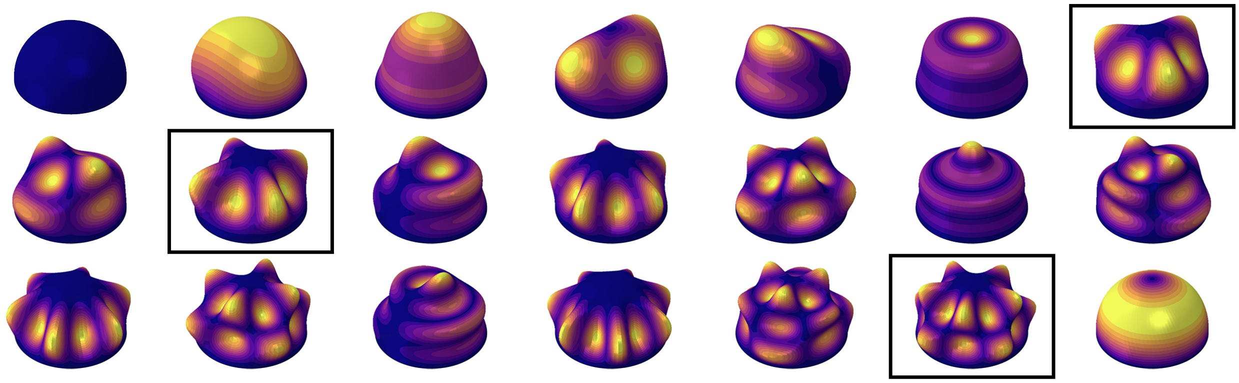

Supplementary Figure 9: **The first 21 unique modal shapes.** The three modes selected for fabrication are highlighted.

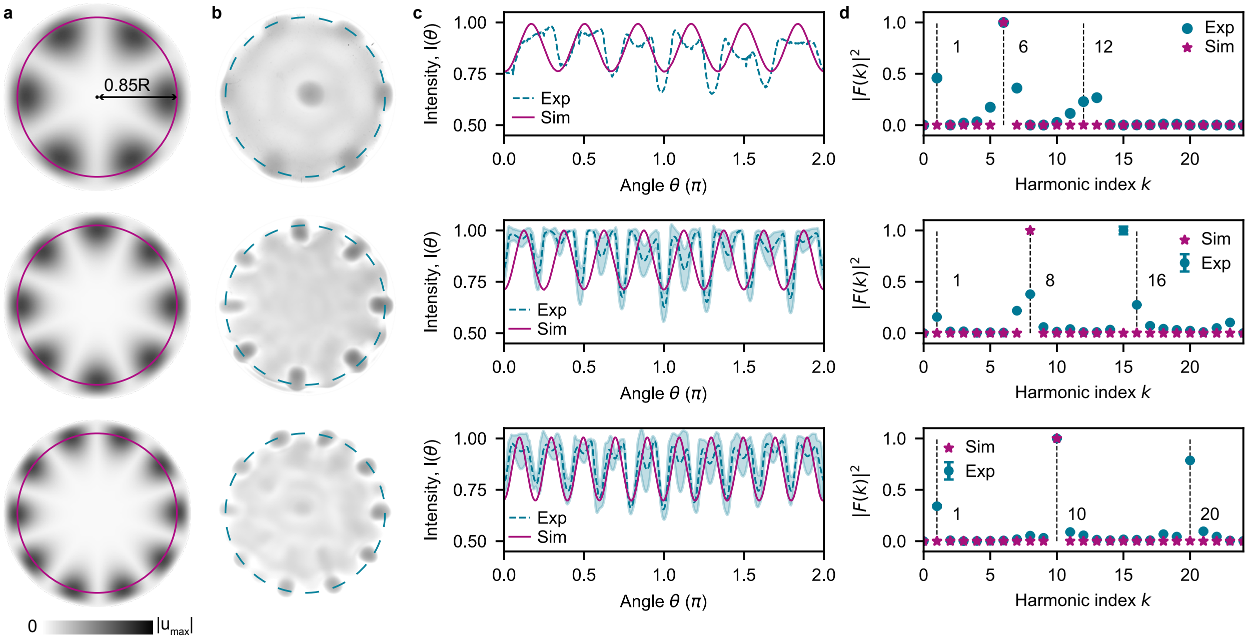

Supplementary Figure 10: **Comparison between modal shapes from FE modal analysis and photographs of imperfect shells.** a. Three modal shapes showing 6, 8, and 10 antinodes in a ring near the perimeter where the greyscale represents normalized displacement magnitudes. b. Photographs of fabricated shells with 6, 8 and 10 bump-like imperfections. c. Intensity plots around a ring at a radius of 0.85R. d) Power spectra showing the magnitude of patterns with different periodicities. Source data are provided as a Source Data file.

simulation intensity measurements for the three specimens are 0.0798, 0.127, 0.0924 respectively. We further observe slight thickening at the nodes of the modal shape as well. To quantify periodicity, we analyze intensity variations using a Fourier transform. Each image is first reduced to a one-dimensional signal by averaging pixel intensities over a circular ring near the defects ($0.85 \pm 0.05R$), yielding an intensity profile I. The profile is then mean-subtracted to remove the zero-frequency component and transformed using discrete Fourier transform.

The resulting Fourier coefficients decompose the signal into harmonics corresponding to periodic variations around the circumference. The magnitude of the coefficient of the power spectrum $|F(k)|^2$ at harmonic index k quantifies the strength of a pattern repeating k times over one revolution. When this is applied to the FE modal displacement, one clear peak is present, representing the number of antinodes in that particular mode (S.Fig. 10d). For the fabricated specimens, the power spectrum similarly shows a local maximum at the requested number of defects. However, other local maxima are also present, specifically, at $k = 1$ and at higher harmonics, *e.g.*, $k = 12$ for the specimens with 6 bump-like defects. The harmonic index of 1 reflects a global imperfection where one side of the hemisphere is thicker than the other. This can be attributed to the imprecision when mounting the mold onto the speaker and the acoustic quality of the speaker itself. The higher harmonics arise from the non-sinusoidal intensity profiles. The indices around the peaks can be attributed to nonlinearities in the acoustic forcing and geometrically nonlinear structural response at high excitation amplitudes. Altogether, these show that the imprinted imperfections can faithfully retain the feature of the natural vibrational shapes from FE modal analysis.

The above analyses show that the features of the thickness profile resulting from vibration-assisted casting process can be indirectly inferred from the FE modal shape. To do so, one would map the displacement field to greyscale intensity, then apply Eq. 4 to calculate the resulting thickness.

4 Mechanical characterization of hemispherical shells

4.1 Experimental setup

We characterize the pressure–volume relationship of the shell under partial vacuum loading. The shell is placed over an acrylic (PMMA) plate with a center hole connected to a syringe pump via a tube. To ensure an airtight seal, a second acrylic plate is positioned above the shell, and the two plates are clamped together with screws, forming a closed system consisting of the air inside the shell, the connecting tubing and the 1 mL syringe on the syringe pump.

Internal pressure is monitored using an MPL3115A2 barometric sensor. Data from the sensor is recorded in real-time with an ELEGOO UNO R3 microcontroller throughout the loading process at a period of 0.8 s.

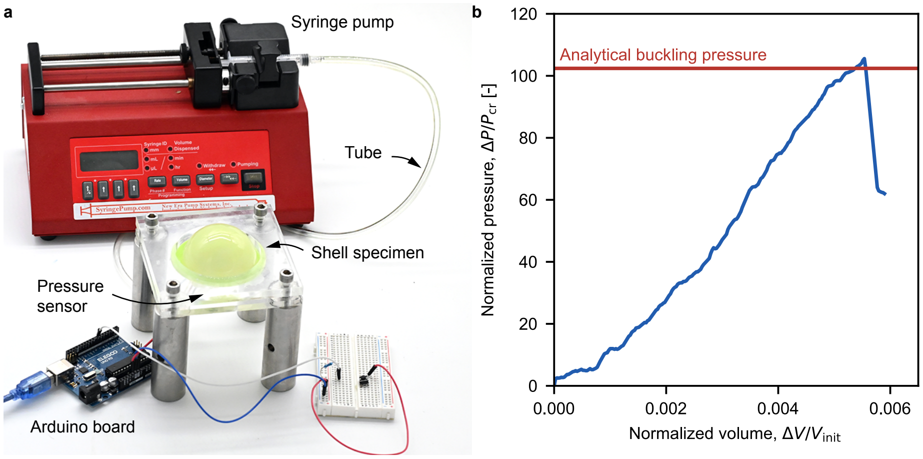

Supplementary Figure 11: **Mechanical testing of thin elastomeric shells.** a. Automated setup to extract air from the interior of the hemispherical shells while simultaneously recording its internal pressure. b. The pressure volume behavior of a near-perfect shell. Source data are provided as a Source Data file.

As the volume inside the closed system expands, the internal volume increases, and the pressure gradually decreases. This causes a pressure difference between the enclosed system and the surrounding. When the pressure difference exceeds a critical threshold, the shell loses stability and buckles, as indicated by the abrupt change in the pressure–volume response.

4.2 Calculation of shell volume

Since the system comprising of the air within the shell, the connected tube and the syringe is closed, and the temperature remains constant throughout the experiment, the pressure–volume relationship can be modeled using the ideal gas law. Under these conditions, the evolution of pressure and volume follows the equation, $p_0 v_0 = p_c v_c$, where p_0 and v_0 are the pressure and the volume of the air in the closed system at the initial state, and p_c and v_c represent the pressure and

the volume at subsequent points in time. Thus, we have,

$$p_0/p_{\mathrm{c}} = v_{\mathrm{c}}/v_0. \tag{5}$$

The total volume is the summation of the volume of the air in the syringe, in the tube and under the shell, *i.e.*, $V^{\mathrm{Total}} = V^{\mathrm{Sy}} + V^{\mathrm{Tu}} + V^{\mathrm{Sh}}$.

We can write Eq. 5, as

$$p_0/p_{\mathrm{c}} = \frac{V_c^{\mathrm{Sy}} + V_c^{\mathrm{Tu}} + V_c^{\mathrm{Sh}}}{V_0^{\mathrm{Sy}} + V_0^{\mathrm{Tu}} + V_0^{\mathrm{Sh}}}, \tag{6}$$

which, after rearrangement, gives,

$$V_c^{\mathrm{Sh}} = \frac{p_0}{p_{\mathrm{c}}}(V_0^{\mathrm{Sy}} + V_0^{\mathrm{Tu}} + V_0^{\mathrm{Sh}}) - (V_c^{\mathrm{Sy}} + V_c^{\mathrm{Tu}}). \tag{7}$$

Since at the initial state there is no volume in the syringe Eq. 7 can be simplified as,

$$V_c^{\mathrm{Sh}} = \frac{p_0}{p_{\mathrm{c}}}(V_0^{\mathrm{Tu}} + V_0^{\mathrm{Sh}}) - (V_c^{\mathrm{Sy}} + V_0^{\mathrm{Tu}}), \tag{8}$$

where,p_0 is the initial, or the atmospheric pressure, in the system, p_c is the pressure measured by the pressure sensor, V^{Sy} is the known volume in the syringe, V^{Tu} is the constant volume in the tube, *i.e.*, $V_0^{\mathrm{Tu}} = V_c^{\mathrm{Tu}}$ and V_c^{Sh} is the volume of air under the hemispherical shell. This allows us to plot the pressure-volume curves in Figure 5 of the manuscript.

4.3 Calculation of critical pressure of a perfect shell

For a geometrically perfect spherical shell under uniform compression, the critical buckling pressure in given by [2] as follows,

$$P_{\mathrm{cr}} = \frac{2E}{\sqrt{3(1-\nu^2)}}\left(\frac{t}{R}\right)^2. \tag{9}$$

For the geometry and material in our study, we substitute the following, $E = 1.2\,\mathrm{MPa}$, $\nu = 0.5$, $t = 0.2\,\mathrm{mm}$, $R = 25.4\,\mathrm{mm}$ and arrive at $P_{\mathrm{critical}} = 102.4\,\mathrm{Pa}$.

To verify that our fabrication prototype can reproduce shells that are geometrically near perfect, we turn off the acoustic perturbation. We experimentally test the shell using the method above and plot the resulting pressure volume curve in comparison to the analytical value (S.Fig. 11b).

4.4 Stiffness measures

The stiffness is measured from the quasi-linear portion of the normalized pressure-volume curves prior to the onset of instability is given in (Table 1). These normalized stiffness quantities are calculated as $E_{\mathrm{norm}} = p_{\mathrm{norm}}/V_{\mathrm{norm}}$ over the initial linear regime ($V_{\mathrm{norm}} = 0.002$). They are plotted in Fig. 5b of the manuscript.

To quantify the reduction in structural capacity with increasing excitation amplitude, we fit the measured knockdown factors to an exponential decay model $\kappa = \alpha \exp(-k \cdot v)$, where v is the normalized speaker volume, α is a shared pre-factor common to all modal families, and k is a mode-dependent decay constant. The fitting is performed using nonlinear least-squares regression (`scipy.optimize.curve_fit`).

Table 1: Linearized stiffness measure of the different shell specimens.

	**No volume** [−]	**Volume 1** [−]	**Volume 2** [−]	**Volume 3** [−]
**Near-perfect shell**	119.7 ± 10.54			
**6 bump-like defects**		115.5 ± 14.09	115.1 ± 7.685	72.21 ± 12.61
**8 bump-like defects**		133.5 ± 9.091	112.1 ± 1.304	107.9 ± 8.599
**10 bump-like defects**		113.3 ± 8.810	109.6 ± 6.624	104.2 ± 3.334

5 Analysis of fluid motion

The vibration of the mold drives a flow in the liquid silicone layer that is ultimately responsible for the steady deflection of the liquid silicone's free surface. To gain insight into the flow mechanisms that lead to the steady wavy free surface, we develop and analyze a minimal model that captures the essential physics of the problem. We particularly wish to understand why the free surface's wavenumber is twice that of the mold's vibration pattern, and why the free surface deflects outward at the antinodes of the mold's vibration.

Consider a two-dimensional layer of liquid of nominal thickness H that is bounded below by a vibrating surface and above by a free surface (S.Fig. 12). The bottom boundary has a prescribed deformation, and the free surface deforms in response to the flow. We take the bottom boundary's deflection to be given by a standing wave of frequency ω, wavenumber k, and amplitude ϵ,

$$h_L(x,t) = \epsilon \sin(\omega t) \cos(kx). \tag{10}$$

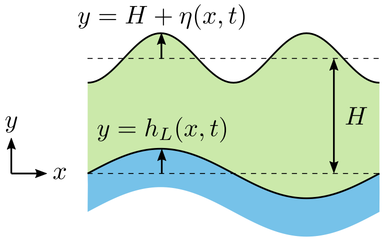

Supplementary Figure 12: **Model problem for the flow of the liquid silicone.** A fluid (green) with a free surface is driven by the motion of a solid boundary (blue).

The liquid silicone in the experiment is non-Newtonian, but its viscosity is weakly affected by shear [1,3], so we treat it as Newtonian for simplicity. Additionally, although the liquid silicone's properties change as it cures, this occurs on a time scale that is much slower than any time scales relevant to the flow, so we treat the liquid's properties as constant.

We perform a weakly nonlinear analysis to gain insight into the flow. This approach has been used in the past to gain insight into other flows driven by vibrating boundaries [4]. Lengths are scaled by H, time by ω^{-1}, velocity by ωH, and pressure by $\mu\omega$, with μ the fluid's viscosity. From here onward, all quantities are dimensionless. We expand the fluid velocity and pressure fields, as well as the deflection of the free surface η from its mean height, in powers of $\epsilon^* = \epsilon/H$,

$$\mathbf{u} = \epsilon^* \mathbf{u}_1 + \epsilon^{*2} \mathbf{u}_2 + \mathcal{O}(\epsilon^{*3}), \tag{11}$$

$$p = \epsilon^* p_1 + \epsilon^{*2} p_2 + \mathcal{O}(\epsilon^{*3}), \tag{12}$$

$$\eta = \epsilon^* \eta_1 + \epsilon^{*2} \eta_2 + \mathcal{O}(\epsilon^{*3}). \tag{13}$$

Substituting these expansions into the Navier-Stokes equations and grouping terms of the same order, the primary flow satisfies

$$\nabla \cdot \mathbf{u}_1 = 0, \tag{14}$$

$$Re\, \partial_t \mathbf{u}_1 = -\nabla p_1 + \nabla^2 \mathbf{u}_1. \tag{15}$$

The secondary flow satisfies

$$\nabla \cdot \mathbf{u}_2 = 0, \tag{16}$$

$$Re\, \partial_t \mathbf{u}_2 = -\nabla p_2 + \nabla^2 \mathbf{u}_2 - Re\, \mathbf{u}_1 \cdot \nabla \mathbf{u}_1. \tag{17}$$

Note that the primary flow appears as a forcing term for the secondary flow. In the experiment, the Reynolds number $Re = \rho\omega H^2/\mu$ is $\mathcal{O}(10^{-2})$, with ρ being the fluid's density.

The full flow field satisfies no-slip and no-penetration boundary conditions at the bottom boundary, while at the free surface it satisfies normal and tangential stress balances. Since in the experiment the free surface of the liquid silicone interfaces with air, the tangential stresses there can be ignored. Similarly, the normal stress balance simplifies to a balance between the normal stress in the liquid and the stress due to surface tension. Additionally, the free surface satisfies a kinematic boundary condition to ensure that its motion is consistent with that of the underlying fluid.

For our purposes, it will suffice to analyze the case where the free surface is fixed to be flat. The resulting pressure distribution at the free surface will then indicate how the free surface would deform once allowed to.

We start by analyzing the primary flow. Its governing equations are all linear and homogeneous, as are its boundary conditions, except for the inhomogeneous no-slip/no-penetration condition at the bottom boundary, which in dimensionless form reads

$$\mathbf{u}_1 = \cos(t)\cos(k^* x)\hat{\jmath} \quad \text{at } y = 0. \tag{18}$$

This simply reflects that the primary flow is driven entirely by the velocity of the bottom boundary. Because of the linearity of the first-order equations, all primary variables will be sinusoidal in time and space with dimensionless frequency 1 and wavenumber k^*. As a result, there is no mean flow, and the free surface does not have a mean deflection at first order. To determine the mean deflection of the free surface, we must move on to the secondary flow.

The secondary flow's governing equations and boundary conditions have inhomogeneities stemming from the primary flow. In particular, the time-averaged flow satisfies

$$0 = -\nabla\langle p_2\rangle + \nabla^2\langle \mathbf{u}_2\rangle - Re\,\langle \mathbf{u}_1 \cdot \nabla \mathbf{u}_1\rangle, \tag{19}$$

where $\langle\cdot\rangle$ denotes time-averaging. Since the primary flow is sinusoidal in time, the associated Reynolds stresses (which lead to the forcing term above) are non-zero, and they create a mean secondary flow, which we will call the streaming flow. In addition, the streaming flow has one inhomogeneous boundary condition,

$$\langle \mathbf{u}_2\rangle = -\frac{1}{\epsilon^*}\langle \partial_y \mathbf{u}_1 h_L\rangle = -\partial_y\langle \mathbf{u}_1 \sin(t)\rangle \cos(k^* x) \quad \text{at } y = 0, \tag{20}$$

which we will refer to as the boundary forcing. Since the x-component of the primary velocity is zero at $y = 0$, continuity implies that only the x-component of the above boundary condition for the streaming flow is inhomogeneous.

The pressure associated with the streaming flow leads to steady deformation of the free surface. If we can determine the spatial structure of the streaming flow's pressure field, then we can determine the form of the free surface's mean deflection. Doing so requires knowledge of the Reynolds stresses and boundary forcing, so we return to the primary flow.

Rather than precisely solving for the flow variables, we will instead deduce the structure of the free surface's mean deflection using physical arguments and the basic mathematical structure of the problem. Letting ψ denote the stream function, it follows that the primary flow's stream function takes the form

$$\psi_1(x, y, t) = f(y)\sin(k^* x + \phi_x)\cos(t + \phi_t), \tag{21}$$

where f is a yet-unknown function, and ϕ_x and ϕ_t are yet-unknown phases. Then the two velocity components are

$$u_1(x, y, t) = \partial_y \psi_1(x, y, t) = f'(y)\sin(k^* x + \phi_x)\cos(t + \phi_t), \tag{22}$$

$$v_1(x, y, t) = -\partial_x \psi_1(x, y, t) = -k^* f(y)\cos(k^* x + \phi_x)\cos(t + \phi_t). \tag{23}$$

The forcing due to the Reynolds stresses is then

$$-\langle \mathbf{u}_1 \cdot \nabla \mathbf{u}_1 \rangle = \frac{1}{4}k^*(ff'' - f'^2)\sin[2(k^* x + \phi_x)]\hat{\imath} - \frac{1}{2}k^{*2} ff' \hat{\jmath}, \tag{24}$$

and the inhomogeneous part of the streaming flow's boundary condition is

$$-\partial_y \langle u_1 \sin(t) \rangle \cos(k^* x)|_{y=0} = \frac{1}{2} f''(0)\sin(\phi_t)\left[\frac{1}{2}\sin(2k^* x)\cos(\phi_x) + \cos^2(k^* x)\sin(\phi_x)\right]. \tag{25}$$

Two features stand out. The first is that the y-component of the forcing from the Reynolds stresses is constant in x, so it will not tell us anything about the mean deflection of the free surface. The second is that the x-component of the forcing from the Reynolds stresses is purely sinusoidal in x with a wavenumber $2k^*$; the same is true for the boundary forcing. Therefore, the mean deflection of the free surface will also be sinusoidal in x with a wavenumber $2k^*$. This is exactly what we see in the experiment: the mean deflection of the free surface has twice the wavenumber of the mold's vibration pattern. The remaining element left to determine is the position of the free surface's mean deflection pattern relative to the bottom boundary.

To this end, we appeal to physical arguments to first determine the spatial phase ϕ_x. Because the problem has a reflection symmetry in the x-direction, from the inhomogeneous boundary condition for the streaming flow we deduce that $\phi_x = 0$ (otherwise it would have a non-zero spatial mean). We can also come to this conclusion by considering the physics of the primary flow. Where the bottom boundary moves up, the fluid is pushed up with it, and where it moves down, the fluid is pulled down with it. By symmetry, the streamlines above the antinodes of the bottom boundary must be vertical. Recalling that the coordinate system is chosen so that the shape of the bottom boundary is $\propto \cos(k^* x)$, it follows that $\phi_x = 0$. Thus, the pattern of the forcing due to the Reynolds stresses, as well as the pattern of the boundary forcing, are $\propto \sin(2k^* x)$.

Knowing that the two forcings are $\propto \sin(2k^* x)$ is not enough, however, since the sign of the pre-factor determines the spatial phase of the forcings. To determine the pattern of the forcing, we require additional knowledge of the primary flow. Where the bottom boundary moves up, the fluid is pushed up with it, which requires an increased pressure locally. Conversely, where the bottom boundary moves down, the fluid is pulled down with it, which requires a decreased pressure locally. The resulting pressure gradient leads to a circulating flow, as sketched in S.Fig. 13.

The qualitative picture of the primary flow is sufficient to determine the pattern of the forcing. The x-component of the forcing due to the Reynolds stresses is the mean of $-\mathbf{u}_1 \cdot \nabla u_1$, which is the

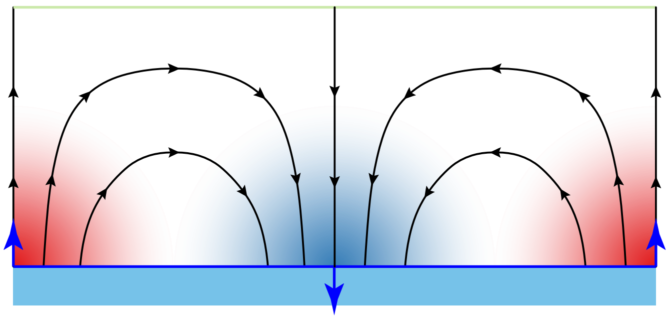

Supplementary Figure 13: **Sketch of the primary flow's pressure distribution along the vibrating lower boundary.** Red indicates high pressure while blue indicates low pressure. The blue arrows indicate the instantaneous motion of the bottom boundary. The pressure gradient leads to a circulating flow, as shown by the streamlines.

negative of the derivative of the horizontal velocity in the direction of the streamlines. Following the pattern of the streamlines, we deduce that the x-component of the forcing is directed toward the antinodes of the bottom boundary. Thus, the forcing due to the Reynolds stresses is $\propto \sin(2k^*x)$ with a negative pre-factor. The Reynolds stress forcing is shown with red arrows in S.Fig. 14. For the boundary forcing, we require $f''(0)$, which is related to $\partial_y u_1|_{y=0}$. From the pattern of the primary streamlines in S.Fig. 13, we deduce that $f''(0) > 0$. Additionally, we also require the temporal phase ϕ_t. For the low Reynolds number in the experiment, the low amount of inertia causes the primary flow to lag the motion of the boundary by a small amount, so ϕ_t is small and positive. (In fact, $\phi_t = \mathcal{O}(Re)$, so the boundary forcing and the forcing due to the Reynolds stresses are of the same order.) The resulting boundary condition for the streaming flow is shown with blue arrows in S.Fig. 14.

Because of the sinusoidal nature of the boundary forcing and the symmetry of the problem, the streamlines of the streaming flow must be vertical above the nodes and antinodes of the bottom boundary's vibration, with the direction of the flow as sketched in S.Fig. 14. From continuity, it follows that the flow takes on a circulatory nature, consisting of circulation cells of alternating sense. To determine the pressure distribution along the top surface, let us focus on the leftmost circulation cell in S.Fig. 14. As the fluid rises from the bottom, the forcing due to the Reynolds stresses accelerates the fluid leftward. Once the fluid starts to approach the upper-left corner of the cell, it must turn downward, which requires that it be accelerated rightward against the forcing of the Reynolds stresses. Thus, the pressure must increase locally above the antinode. The same logic applies to the other cells, leading to the pressure distribution sketched in S.Fig. 14: relatively high pressure above the antinodes, and relatively low pressure above the nodes.

To summarize, the vibration of the bottom boundary drives a time-periodic circulating flow in the fluid. This circulating flow has non-zero Reynolds stresses with twice the wavenumber that, together with a boundary forcing, create a streaming flow. The spatial patterns of the forcings and streaming flow lead to relatively high pressure above the bottom boundary's antinodes and relatively low pressure above the nodes. If the upper surface is allowed to move, it will respond to this pressure distribution, deflecting upward above the antinodes and downward above the nodes. In other words, fluid accumulates above the antinodes of the bottom boundary's vibration, just as in the experiment.

The mechanism described above sets the free surface into motion. Once it is in motion, it will modify the flow. Depending on the values of the parameters, the flow may take on a different form

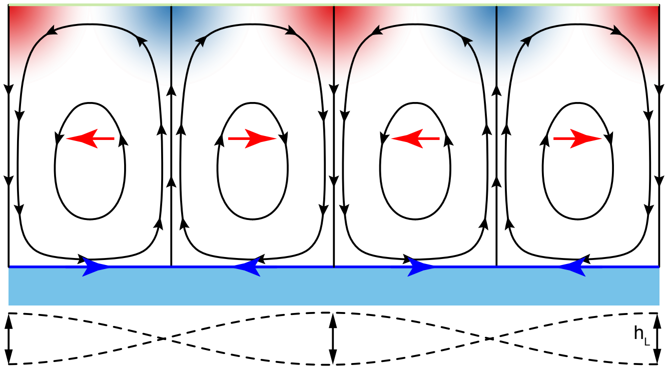

Supplementary Figure 14: **Sketch of the streaming flow's pressure distribution along the free surface.** Red indicates high pressure while blue indicates low pressure. The blue arrows indicate the boundary condition at the lower boundary, which leads to the circulating flow shown by the streamlines. The red arrows show the x-component of the forcing due to the Reynolds stresses. The vibration pattern is shown at the bottom for reference.

than what has been sketched above. The governing dimensionless parameters are the Reynolds number Re, the capillary number $Ca = \mu\omega H/\gamma$ (where γ is the surface tension), the wavenumber k^*, and the amplitude of vibration ϵ^* of the bottom boundary. In the experiment, we have $Re = \mathcal{O}(10^{-2})$, $Ca = \mathcal{O}(10^2)$, and $k^* = \mathcal{O}(10^{-1})$. In this parameter regime, according to the weakly nonlinear theory, the dimensionless amplitude of deflection of the free surface scales as $A^* \sim Re^0 Ca^2 k^{*-2} \epsilon^{*2}$. In dimensional terms, the amplitude scales as $A \sim \lambda^2 \mu^2 \omega^2 \epsilon^2 \gamma^{-2} H^{-1}$, where we have used the wavelength λ of the vibration instead of the wavenumber k. Note that this assumes that the frequency, wavelength, and amplitude of the vibration can be controlled independently.

6 Supplementary Movies

Supplementary Movie 1 Casting procedure and visualization of the vibration and fluid accumulation

Supplementary Movie 2 Vibration of the elastic hemispherical mold as visualized using slow-motion videography

Supplementary Movie 3 Migration of the fluid as a result of the structural vibration as visualized using reflective particles

References

[1] Anna Lee, P-T Brun, J Marthelot, G Balestra, F Gallaire, and Pedro M Reis. Fabrication of slender elastic shells by the coating of curved surfaces. *Nature communications*, 7(1):11155, 2016.

[2] Robert Zoelly. *Ueber ein Knickungsproblem an der Kugelschale.* Buchdr. Zürcher & Furrer, 1915.

[3] Ruslan Yu Lukin, Aidar M Kuchkaev, Aleksandr V Sukhov, Giyjaz E Bekmukhamedov, and Dmitry G Yakhvarov. Platinum-catalyzed hydrosilylation in polymer chemistry. *Polymers*, 12(10):2174, 2020.

[4] Jérôme Hoepffner and Koji Fukagata. Pumping or drag reduction? *Journal of Fluid Mechanics*, 635:171–187, 2009.
