# Supplementary material for: Vibration-assisted fabrication of thin shells with spatially distributed imperfections
Source: Nat Commun. 2026 May 20;17:7324. doi: 10.1038/s41467-026-73343-2 (PMC13402623; doi:10.1038/s41467-026-73343-2)
Supplement: Supplementary file 2 — Description of Additional Supplementary Files [file 41467_2026_73343_MOESM2_ESM.pdf]

Description of Additional Supplementary Files

Supplementary Movie 1. Casting procedure and visualization of the vibration and fluid accumulation

Supplementary Movie 2. Vibration of the elastic hemispherical mold as visualized using slow-motion videography

Supplementary Movie 3. Migration of the fluid as a result of the structural vibration as visualized using reflective particles
