# Supplementary material for: Vibration-assisted fabrication of thin shells with spatially distributed imperfections
Source: Nat Commun. 2026 May 20;17:7324. doi: 10.1038/s41467-026-73343-2 (PMC13402623; doi:10.1038/s41467-026-73343-2)
Supplement: Supplementary file 6 — Transparent Peer Review file [file 41467_2026_73343_MOESM6_ESM.pdf]

nature portfolio

Peer Review File

Vibration-Assisted Fabrication of Thin Shells with Spatially Distributed Imperfections

Corresponding Author: Dr Tian Chen

This file contains all reviewer reports in order by version, followed by all author rebuttals in order by version.

Version 0:

Reviewer comments:

Reviewer #1

(Remarks to the Author)
Review of:
Acoustic-Assisted Fabrication of Thin Shells with Spatially Distributed Imperfections

by:
Ilyes Krida, Jacob Tang, Daniel Floryan, and Tian Chen

This manuscript presents a new method for fabricating thin shells with spatially distributed imperfections that follow the eigenmodes obtained by dynamically exciting a silicone mold. The dynamic excitation is provided through the vibration of the inside air of the hemispherical shell through a speaker. This clever method enables different eigenmodes as well as intensities (amplitudes) of imperfections in a rather controlled fashion. Along with this feature of tunability, the next strength of the proposed fabrication method lies in the fact that such patterns follow a natural (dynamic) response of the shell. Such obtained patterns in my experience serve as an excellent tool to explore experimentally the effects of imperfection on the stability of shells. How meaningful is this for practical shell applications, where imperfections are some random dents resulting from manufacturing defects, transport, loading, environmental influence, etc., is another story. The paper is well written and the results that are provided are presented properly.

More specific remarks (+):
The paper answers the first few questions the reader has after going through the abstract, namely:
- on the resemblance of the experimentally obtained eigenmodes and numerically predicted ones. It is nice to see that the proposed fabrication method works in this aspect – at least qualitatively.
- on the excitation frequency determined in the fabrication process (that is informed by the FE analysis). Maybe this could be mentioned earlier in the paper, e.g., where first mentioned that the shells are excited by the speaker.

(-):
1. The paper includes a good review of modern contributions. Maybe, but not necessary, few historically important contributions could be mentioned. Some of them can be found in the cited literature.

2. “High speed imaging and uCT scanning reveal accumulation of material at the antinodes of the mold’s vibrational modes.” Is it really in the antinodes or specifically only in the bottom antinodes? As far as I am familiar with vibration of shells and plates one can find standing (half-)waves in regions that are bulged inward AND outward, forming antinodes.

3. “In our setup, by actively exciting a thick elastic hemispherical mold using a speaker with tunable frequencies and volume, we induce…” I would rephrase the sentence into: “In our setup, by actively exciting a thick elastic hemispherical mold with a speaker by adjusting a frequency and volume, we induce…” or something like that. I my opinion phrasing “speaker with tunable frequencies” sounds odd.

4. What is the thickness distribution over the polar angle of the hemispherical shell on the line where there is no

concentration of the material? In my experience the deviation from the apex to the equator could be 15 % and more in a completely static setting, depending on the viscosity of the liquid silicone. How does this affect the fabrication procedure and post-analyses of the buckling resistance?

5. the comparison in Fig. 4 is only visual. Why is there no quantification of results?

6. page 5: “These findings confirm that the acoustic excitation protocol introduces repeatable, spatially structured imperfections whose geometry closely resembles vibrational mode shapes.” I could not find the proof of that in the paper.

7. page 6: “under increasing vacuum pressure (Fig. 5).” This phrase sounds a bit awkward or ambiguous. Is the pressure increasing (less vacuum), or is the vacuum getting stronger (less pressure)? One can easily figure it out when learning about the buckling experiment with vacuuming the inner space of the shell, i.e. by applying the outside pressure (which is limited by the amount of the atmospheric pressure).

8. page 6: “The stiffness of all shells remains relatively constant.” What does this mean quantitatively? There is no proof of that given in the paper. (I hope I did not miss it.)

9. caption of Fig. 5: of of

10. “Quantitative measurements from μCT and optical imaging confirm the high fidelity of modal patterning, while mechanical buckling tests demonstrate a systematic reduction in structural capacity with increasing imperfection amplitude.” How is the high fidelity confirmed?

11. What is the range of achievable eigenmodes?

12. (just out of curiosity) Does it make sense to use mixed modes?

13. Was the effect of boundary conditions checked (I am referring to repeatability)? In other words, does something change if the silicone mold is demounted and mounted again? The boundary condition might change a little.

Nevertheless, while the proposed experimental approach is intriguing, the work lacks at least some basic statistical characterization. The question on robustness / repeatability remains open in my opinion, which is not good.

14. The reproducibility of thickness, (very importantly) patterns and consequently knockdown factor measurements must be quantified. I recommend presenting the results in means +/- SD for thickness, imperfection amplitude and critical pressure over at least three independent samples per fixed set.

Technically, the speaker essentially induces structural vibration of the mold through the air volume inside the shell. The term “vibration-assisted” would therefore be far more precise. Please note, that no acoustic effects were found on the shell. This comment does/should not diminish the cleverness of the proposed method.

15. Acoustic --> Vibration?

I recommend minor revisions before the manuscript can be considered for publication. These minor revisions can become major revisions if the authors did not save enough data for at least a basic statistical analysis.

Reviewer #2

(Remarks to the Author)
This manuscript introduces an interesting vibration-assisted casting method to imprint modal imperfections into thin shells. It could contribute to further work on imperfection-sensitive buckling and could find broader applications in soft matter engineering. However, in my opinion there are some conceptual points that need to be further clarified, and more importantly, although the authors propose this clever manufacturing procedure, no real advance is obtained in the understanding and prediction of buckling properties of architectured or defected shells. Therefore, I am not convinced that the work entails a sufficiently important breakthrough (“to advance understanding in a way that will move the field forward”) to be published in Nature Communications.
Main Comments:
- The manuscript refers to the acoustically imprinted thickness variations as “imperfections”, but the patterns are deliberately engineered, so it would be more appropriate to speak of “architectured features”. I think the term “imperfections” is potentially misleading and inconsistent with prior literature.
- Although the experiments are well-conceived and explained, and good agreement is found with FEM simulations, they would benefit from additional control experiments to provide a quantitative baseline (i.e., casting without acoustic excitation). For example, it would be useful to have data on shell thickness uniformity in the absence of vibration.
- The authors state that “the accumulations coincide with the locations of the antinodes (Fig. 2d). This seems counterintuitive as the antinodes experience the largest vibration amplitude and could cause the liquid to flow away”. This point would

require more discussion and analysis. The explanation of how and why material accumulates at antinodes is only qualitative and , as the authors indicate, somewhat counterintuitive. The reference to secondary streaming is plausible but not backed up with flow measurements or simulations. A simplified quantitative model might be useful, or at least reference to detailed hydrodynamic theory, to support this behaviour.
- The role of material properties is not discussed in the mechanism, e.g. elasticity, viscosity, rheology of the silicone during curing, curing time vs flow time. It would be important to try to analyse quantitatively how material properties influence the final shell shape.
- The match between FEM-computed vibration modes and measured shell geometries is mostly qualitative. A more rigorous quantitative comparison would strengthen this connection. It would also be interesting to see if more complex architectured structures can be created, e.g. by exciting at multiple frequencies and exploiting the superposition of modes.
- As mentioned, the authors propose a very clever manufacturing procedure to introduce architectured features in a hemispherical shell, demonstrating that the presence of these is correlated with the knock-down factor of the structure (which is not surprising). The correlation with speaker volume is only phenomenological, and can be used for practical manufacturing applications, but does not contribute to a deeper understanding of the buckling of defected hemispherical shells. To achieve broader impact, the authors might try to suggest more specific applications in programmable materials and soft robotics, as they are generically mentioned only briefly. A demonstration, prototype, or at least a more detailed conceptual framework for these applications would enhance the significance of the work.

Minor details:
Page 1: “Yet, it remains an active research topic on what “physically realistic” geometric imperfections most severely reduce the knockdown factor”. This phrase is unclear and somewhat awkward.
Page 3: “between successive coat” should be “between successive coatings”
Page 3: “Once we achieve a mold thickness that exceeds the thickness of the eventual hemispherical shells by an order of magnitude”: why an order of magnitude?
Page 3: “The frequency and volume of speaker is digitally…” should be “The frequency and volume of the speaker is digitally…”
Page 3: “(frame rate equally to the frequency of the speaker)” should be “(frame rate equal to the frequency of the speaker)”
Page 4: “A laser sheet is projected across the shell of radius r at a given elevation, z = 0.25r, to highlights the location of nodes and antinodes by recording the distortions along the intersection between the elastic mold and the laser (Fig. 2b).” . It is not clear what is being measured and how. Also, “highlights” should be “highlight”. Finally , reference to Fig. 2b does not match what is shown. Please revise.
Page 4: “The same laser sheet photography from the top view shows that the accumulations coincide with the locations of the antinodes”. Is there also a grading effect in the thickness as you move away from the antinode location?
Page 4: “This secondary streaming flow is much weaker than the primary oscillatory flow, and bears resemblance to the streaming flow associated with Faraday waves”: doesn’t really explain why the material is accumulating at the antinodes, please expand.
Page 4, section on flow dynamics: consider adding more description of the observed toroidal circulation in Fig. 2e. Does this circulation contribute to the steady-state accumulation pattern?
Page 5: “…with changing its characteristics.” should be “by changing its characteristics.”
Page 6: “Full-field non-linear dynamical analysis will be conducted in the future to quantitatively construct this correlation”. Why not in this work? It would increase its interest and impact
Page 6: “Increasing the speaker volume results in a systematic reduction in critical buckling pressure and earlier onset of instability.” This is not surprising, given the cited literature. It would be more remarkable to gain a quantitative prediction of this reduction.
Page 7: “This technique opens new opportunities for studying multi-mode and nonlinear imperfection interactions, as well as programmable buckling responses.” This work probably provides an additional tool to study these aspects, but does not address them directly. It would gain considerably in interest if it did

Reviewer #3

(Remarks to the Author)
Nature Communications manuscript NCOMMS-25-50095
Paper Title: Acoustic-Assisted Fabrication of Thin Shells with Spatially Distributed Imperfections
Authors: I. Krida, et al.
Review Report:
In this manuscript, the authors present an acoustic-assisted method for fabricating thin shells with spatially distributed, vibrational mode-shaped imperfections. Through experiments, they demonstrate that the geometry of these imperfections can be customized by tuning the acoustic frequency and intensity of a standing wave. The authors further suggest that this technique could enable new opportunities in the development of morphable surfaces, acoustically controlled soft robots, and artificial shells with programmed buckling behavior.
However, I regret to say that I do not find this work suitable for publication in a high-impact journal such as Nature Communications. The manuscript lacks sufficient novelty, originality, and scientific depth. The proposed method is straightforward and does not demonstrate significant scientific rigor.
Major concerns:
1. Research Motivation: The motivation for this research is not clearly articulated. It is unclear why the artificial introduction of imperfections into thin shell structures is necessary or significant. While the authors briefly mention potential applications related to buckling (in Discussion), the overall motivation remains vague, making it difficult for readers to appreciate the importance of the work.

2. Methodological Simplicity: The methodology—casting silicone onto a thick elastic mold excited by a speaker, with vibration-induced flow during curing creating thickness variations—is overly simplistic. The manuscript does not provide sufficient scientific merit or rigor in the experimental design or analysis.
3. Incremental Advancement: When compared to existing studies (e.g., Anna Lee et al., "Fabrication of slender elastic shells by the coating of curved surfaces," Nature Communications, 7(1):11155, 2016; Anna Lee et al., "The geometric role of precisely engineered imperfections on the critical buckling load of spherical elastic shells," Journal of Applied Mechanics, 83(11):111005, 2016), the current work appears to be incremental and closely follows established principles.
4. Lack of Theoretical Analysis: The manuscript lacks theoretical analysis to support parameter selection and optimization during fabrication. This omission limits the scientific contribution and understanding of the process.
5. Manuscript Preparation: The manuscript contains typographical errors (e.g., "artificial" in the last paragraph), which reflect a lack of careful preparation.

In summary, the quality and scientific contribution of this paper fall short of the standards expected for publication in Nature Communications.

Reviewer #4

(Remarks to the Author)
This paper presents a novel acoustic-assisted fabrication method for introducing spatially distributed, mode-shaped imperfections into thin shells. This work demonstrates significant innovation in the controllable fabrication of imperfections, surpassing traditional methods for introducing localized defects. It provides a powerful and scalable platform for systematically studying the influence of global imperfection fields on shell buckling. Prior to publication, the authors should consider the following reviewer comments:

1. It is recommended to more explicitly emphasize in the Introduction or Discussion the unique advantages of this method compared to existing techniques (such as the indentation or flexible mold methods mentioned in references [15-19]) and the research gap it fills.

2. The paper attributes the imperfection formation to secondary mean flow and provides valuable insight into the physical mechanism by observing particle migration towards the antinodes. However, the in-depth physical explanation for why the flow leads to material accumulation at the antinodes – which seems counter-intuitive given the maximum vibration amplitude at these points – is somewhat underdeveloped. It is suggested to explore this specific flow mechanism in more detail in the Discussion, citing more specific fluid mechanics theories and conducting a more in-depth quantitative analysis. Particularly, attempting to clarify the quantitative relationship between vibration velocity, frequency, and the accumulated thickness/radius is crucial for defect design.

3. The imperfection shapes formed in this study are all standard vibration modes of the elastic hemispherical shell structure. The authors should further consider how to generate triangular, rectangular, or even other irregular shapes on the mold, which is of great significance for translating the research findings into practical engineering applications. It is suggested to pre-design the mold with specific geometries based on its vibrational characteristics. Try to give an example.

Version 1:

Reviewer comments:

Reviewer #1

(Remarks to the Author)
The authors have addressed all major questions and responded to the points raised. I believe that these substantial revisions and updates have elevated the manuscript to a level suitable for publication in Nature Communications. I recommend the paper for publication.

Reviewer #2

(Remarks to the Author)
I am satisfied with the thorough way in which the authors have addressed most if not all the reviewer comments. I think the elements of novelty have been more clearly specified and the remaining unclear aspects have been addressed, so that I feel it merits publication in NatComm

Reviewer #3

(Remarks to the Author)
After carefully reviewing the revised manuscript, I must unfortunately reiterate my earlier recommendation for rejection. Although the authors have attempted to address my previous comments, the paper still does not, in my view, meet the standards required for publication in NC. In particular, I continue to have the following major concerns: not clear motivation, incremental contribution, and quality of presentation (the overall quality of the manuscript remains below the journal’s standards. This includes, but is not limited to, the figures and drawings, many of which are unclear, lack sufficient resolution or labeling, and do not effectively support the main arguments).

Please also the authors shall clarify why corresponding author's affiliation and email adress have been changed?

Reviewer #4

(Remarks to the Author)
The authors have made an extremely high-level revision in response to my concerns. They clearly articulate the academic contribution of the study, profoundly elucidate the fluid mechanics formation mechanism of the physical phenomenon, and further creatively achieve the fabrication of other irregular shapes. Therefore, I strongly recommend its acceptance for publication in Nature Communications.

Open Access This Peer Review File is licensed under a Creative Commons Attribution 4.0 International License, which permits use, sharing, adaptation, distribution and reproduction in any medium or format, as long as you give appropriate credit to the original author(s) and the source, provide a link to the Creative Commons license, and indicate if changes were made.
In cases where reviewers are anonymous, credit should be given to 'Anonymous Referee' and the source.
The images or other third party material in this Peer Review File are included in the article's Creative Commons license, unless indicated otherwise in a credit line to the material. If material is not included in the article's Creative Commons license and your intended use is not permitted by statutory regulation or exceeds the permitted use, you will need to obtain permission directly from the copyright holder.
To view a copy of this license, visit https://creativecommons.org/licenses/by/4.0/

Response to the reviewers' comments of manuscript:

Vibration-Assisted Fabrication of Thin Shells with Spatially Distributed Imperfections
by
Ilyes Krida, Jacob Tang, Leo Mangalath, Daniel Floryan, Tian Chen

Please refer to the "diff. version" appended with our resubmission for a detailed account of all the changes, corrections and additions to the revised version of the manuscript. The revised/new text is highlighted in **red**.

In the following, we address each of the Reviewers' comments/suggestions. In addition, we have included five appendices to address the concerns multiple reviewers have brought up. The numbering of the comments (e.g., R1C1, R1C2, ..., R2C1, R2C2,...) is added by us to aid in cross-referencing our responses. The rebuttals are blue whereas the changes to the manuscript that we have replicated in the rebuttal are highlighted in **red**.

Reviewer 1

This manuscript presents a new method for fabricating thin shells with spatially distributed imperfections that follow the eigenmodes obtained by dynamically exciting a silicone mold. The dynamic excitation is provided through the vibration of the inside air of the hemispherical shell through a speaker. This clever method enables different eigenmodes as well as intensities (amplitudes) of imperfections in a rather controlled fashion. Along with this feature of tunability, the next strength of the proposed fabrication method lies in the fact that such patterns follow a natural (dynamic) response of the shell. Such obtained patterns in my experience serve as an excellent tool to explore experimentally the effects of imperfection on the stability of shells. How meaningful is this for practical shell applications, where imperfections are some random dents resulting from manufacturing defects, transport, loading, environmental influence, etc., is another story. The paper is well written and the results that are provided are presented properly.

We wish to thank the reviewer for their encouraging comments and for remarking the ingenuity of the work.

More specific remarks (+):
The paper answers the first few questions the reader has after going through the abstract, namely:

R1C1. on the resemblance of the experimentally obtained eigenmodes and numerically predicted ones. It is nice to see that the proposed fabrication method works in this aspect – at least qualitatively.

We thank the reviewer for this positive comment. We are pleased that the correlation between simulated vibrational modes and experimentally realized imperfections was clear. In the revised manuscript, we further strengthen this point by adding a quantitative measure of similarity between the experimental imperfection fields and the FE eigenmodes. This is discussed in detail in **R1C7**.

R1C2. On the excitation frequency determined in the fabrication process (that is informed by the FE analysis). Maybe this could be mentioned earlier in the paper, e.g., where first mentioned that the shells are excited by the speaker.

We have revised the manuscript so that the connection between the Finite Element (FE) modal analysis and the experimentally applied excitation frequencies is introduced at the first mention of speaker excitation. This clarifies that the frequencies used in fabrication are preselected based on FE simulations of the mold's vibrational modes, rather than chosen empirically.

We note that, due to uncontrolled imperfections in the mold fabrication and how it mounts to the speaker, as well as the finite fidelity of the acoustic speaker itself, the experimentally selected excitation frequencies may deviate slightly from the ideal FE-predicted modal frequencies. In practice, the excitation frequency is tuned within a narrow band around each predicted mode to most clearly observe the vibrational response of the mold.

We have added the following sentence to the first paragraph of the *Results* section to clarify this procedure:

Prior to fabrication, we first determine the natural vibration modes of the elastic mold through Finite Element (FE) analysis. The frequencies used in the fabrication experiments are then chosen to coincide with these computed modal frequencies; due to mold manufacturing and mounting imperfections and the sound reproduction quality of the acoustic speaker, the experimentally selected frequencies may deviate slightly from the FE predictions.

(-):
R1C3. The paper includes a good review of modern contributions. Maybe, but not necessary, few historically important contributions could be mentioned. Some of them can be found in the cited literature.

We have expanded the introduction to more explicitly acknowledge the classical foundations of shell buckling and imperfection sensitivity. Specifically, we provide historical continuity between early analytical work and modern experimental studies. In addition to Zoelly and Koiter, we now reference seminal contributions by Donnell, Batdorf, von Kármán, Tsien, Babcock, and Hutchinson. These works established the connection between postbuckling behavior, imperfection sensitivity, and the emergence of empirical knockdown factors. These additions allow our work to be placed within the broader historical development of shell stability theory and clarify how modern fabrication-based approaches build upon long-standing theoretical and experimental challenges.

The added text is reproduced here:

This imperfection sensitivity has posed a fundamental challenge in shell mechanics for over a century. Early analyses by Zoelly [5] provided the first closed-form expression for the critical pressure of an ideal, perfectly spherical shell, while subsequent developments by Donnell and Batdorf established governing equations and stability criteria for cylindrical and spherical shells under external pressure [6, 7]. Von Karman later demonstrated that equilibrium states involving large deflections could be sustained at loads far below the classical prediction [8–10]. Tsien further attributed the discrepancy between theory and experiment to the extreme instability of the postbuckling path in the presence of arbitrary disturbances [11]. A unifying theoretical framework was established by Koiter, whose asymptotic postbuckling theory demonstrated that infinitesimal geometric imperfections can induce dramatic reductions in critical load [12]. Following this, extensive analytical and numerical studies sought to reconcile theory and experiment by examining the influence of thickness variations, nonuniform loading, boundary conditions, prebuckling deformations, and geometric imperfections

[13, 14]. Focusing on cylindrical shells, Babcock performed systematic comparisons of different imperfection types and concluded that geometric imperfections dominate buckling sensitivity [15].

Despite these advances, experimental progress has historically lagged behind theoretical and computational developments. As a result, practical shell design continues to rely on classical predictions augmented by empirical knockdown factors [8]. While careful experimental validation has been achieved for cylindrical shells and, in fewer cases, spherical shells [12, 13], traditional fabrication methods often introduce uncontrolled and stochastic defect fields, making deterministic links between imperfection geometry and load-bearing capacity difficult to establish. This limitation has motivated statistical approaches to shell buckling [16], as well as recent efforts to fabricate shells with controlled, prescribed imperfections [17–19].

—

[5] Robert Zoelly. Ueber ein Knickungsproblem an der Kugelschale. Buchdr. Zurcher & Furrer, 1915.
[6] Lloyd Hamilton Donnell. Stability of thinwalled tubes under torsion. Technical report, 1935.
[7] SB Batdorf. A simplified method of elastic-stability analysis for thin cylindrical shells. Technical report, 1947.
[8] Th Von Karman and Hsue-Shen Tsien. The buckling of spherical shells by external pressure. Journal of the Aeronautical Sciences, 7(2):43–50, 1939.
[9] Th Von Karman, Louis G Dunn, and HsueShen Tsien. The influence of curvature on the buckling characteristics of structures. Journal of the Aeronautical Sciences, 7(7):276–289, 1940.
[10] Theodore Von Karman and Hsue-Shen Tsien. The buckling of thin cylindrical shells under axial compression. Journal of the Aeronautical Sciences, 8(8):303–312, 1941.
[11] Hsue-Shen Tsien. A theory for the buckling of thin shells. Journal of the Aeronautical Sciences, 9(10):373–384, 1942.
[12] Warner Tjardus Koiter. On the stability of elastic equilibrium. National Aeronautics and Space Administration, 1967.
[13] John W Hutchinson. Imperfection sensitivity of externally pressurized spherical shells. 1967.
[14] JMT Thompson. The elastic instability of a complete spherical shell. Aeronautical Quarterly, 13(2):189–201, 1962.
[15] CD Babcock. Shell stability. 1983.
[16] Luis Augusto Godoy. Thin-walled structures with structural imperfections. Elsevier, 1996.
[17] Anna Lee, P-T Brun, J Marthelot, G Balestra, F Gallaire, and Pedro M Reis. Fabrication of slender elastic shells by the coating of curved surfaces. Nature communications, 7(1):11155, 2016.
[18] Joel Marthelot, Francisco L´opez Jim´enez, Anna Lee, John W Hutchinson, and Pedro M Reis. Buckling of a pressurized hemispherical shell subjected to a probing force. Journal of Applied Mechanics, 84(12):121005, 2017.
[19] Arefeh Abbasi, Fani Derveni, and Pedro M Reis. Comparing the buckling strength of spherical shells with dimpled versus bumpy defects. Journal of Applied Mechanics, 90(6):061008, 2023.

R1C4. “High speed imaging and uCT scanning reveal accumulation of material at the antinodes of the mold’s vibrational modes.” Is it really in the antinodes or specifically only in the bottom antinodes? As far as I am familiar with vibration of shells and plates one can find standing (half-)waves in regions that are bulged inward AND outward, forming antinodes.

Indeed, in a standing-wave pattern both inward and outward lobes correspond to displacement antinodes. In our setup, the elastic mold oscillates about its mean surface at frequencies of approximately 100–300 Hz, which is several orders of magnitude faster than the timescale of material redistribution in the uncured silicone (~1–5 s). As a result, the silicone does not respond to the

instantaneous direction of motion but rather to the **time-averaged effect of the vibration amplitude**. Regions corresponding to antinodes therefore experience the largest oscillation amplitude, while nodes experience minimal motion.

The fluid motion can be decomposed into a dominant linear oscillatory component and a weaker, higher-order component that includes a steady streaming flow. It is this secondary streaming flow that drives material accumulation at regions of maximum oscillation amplitude (the displacement antinodes), independent of whether the instantaneous motion is inward or outward. Gravity biases the overall symmetry of the flow through drainage effects but primarily acts as an additional body force on the accumulating material, rather than determining the spatial location of accumulation. A detailed analysis supporting this interpretation is provided in Appendix I: Analysis of the fluid motion included with this rebuttal (and in the revised SI Section 5).

We have revised the manuscript text accordingly to clarify that accumulation occurs at displacement antinodes defined by maximum vibration amplitude, rather than at antinodes of a particular sign. The revised text explicitly notes that the observed accumulation reflects a time-averaged streaming flow driven by oscillation amplitude rather than displacement direction, with gravity providing a secondary bias.

i.e., where the magnitude of the vibration amplitude is at a maximum in a standing-wave pattern. [...] Here, gravity may bias the overall symmetry of the flow by acting as a body force on the accumulating fluid.

R1C5. “In our setup, by actively exciting a thick elastic hemispherical mold using a speaker with tunable frequencies and volume, we induce…” I would rephrase the sentence into: “In our setup, by actively exciting a thick elastic hemispherical mold with a speaker by adjusting a frequency and volume, we induce…” or something like that. I my opinion phrasing “speaker with tunable frequencies” sounds odd.

We have revised the sentence to clarify that the *excitation frequency and volume* are controlled externally. The revised phrasing now reads:

In our setup, by actively exciting a thick elastic hemispherical mold with a speaker, and adjusting the excitation frequency and output volume, we induce resonant modal deformation of the mold surface during the curing of a thin liquid silicone layer.

R1C6. What is the thickness distribution over the polar angle of the hemispherical shell on the line where there is no concentration of the material? In my experience the deviation from the apex to the equator could be 15 % and more in a completely static setting, depending on the viscosity of the liquid silicone. How does this affect the fabrication procedure and post-analyses of the buckling resistance?

We agree that in static casting, gravitational drainage and surface tension can produce axisymmetric thickness variations along the polar angle. This was studied at length in *Lee, Anna, et al. Nature communications 7.1 (2016): 11155*, where they observed that the deviation is ±6.6% relative to the mean thickness for VPS32, the exact same silicone we use.

Literature suggests that this deviation from uniform thickness as a baseline does not have a strong influence on the critical buckling pressure. In a subsequent work (to the aforementioned *Nat. Comm.*

article) where imperfections were introduced (*Lee, et al. "The geometric role of precisely engineered imperfections on the critical buckling load of spherical elastic shells." Journal of Applied Mechanics 83.11 (2016): 111005*), it was stated that uncontrolled imperfections such as variations in the shell thickness (the ±6.6% stated above), air bubbles, inhomogeneity in the mixture and surface roughness of the mold are overshadowed by the intentionally-induced imperfections. In a later study where multiple imperfections of various amplitudes were introduced, it was found that if they were spaced apart sufficiently, the largest imperfection dominated the buckling pressure (Derveni, Fani, et al. Mechanics of Materials 204 (2025): 105295.).

Nevertheless, to quantify this baseline effect, we fabricated three additional near-perfect shells without acoustic excitation (i.e., *static casts*) but otherwise using the identical setup as our subsequent fabrication protocols (thick elastic hemispherical mold atop the speaker). This differs with *Lee, et al.* only that their mold is a solid metallic sphere whereas ours is a thick elastic hemisphere.

The thickness profiles of these near-perfect shells are measured both destructively (cutting and microscopy) and non-destructively (photography). The details of both procedures are provided in Appendix II of this rebuttal (which is included in the revised SI). The results (RFig. 1) confirm that the polar thickness variation in these static shells remains within the same range reported in the literature and does not introduce significant non-axisymmetric features.

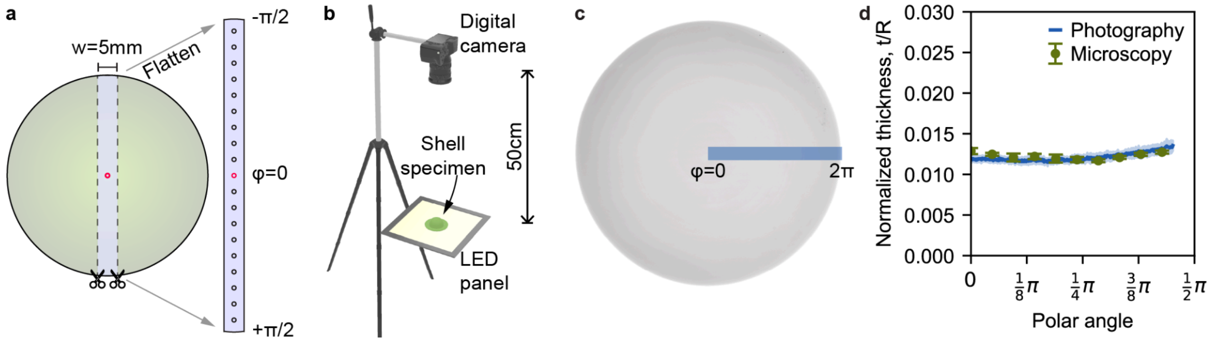

RFig. 1. [SI Fig. 6 and 8] Destructive and non-destructive measurement of shell thickness profiles. a. The segment extracted from the near-perfect shells. The segment is flattened and its thickness is measured from end to end. b. Photography setup to capture shell thickness profile. c. calibrated photograph of a near-perfect shell and d. thickness profile along one azimuthal angle across polar angles from 0 to /2 as directly measured and as inferred through photography.

The following is included in the manuscript, whereas the Appendix II of this rebuttal has been included in the SI in its entirety.

Even in the absence of acoustic excitation, static casting of thin shells can produce axisymmetric thickness variations along the polar angle due to gravitational drainage and surface tension effects. For the silicone used in this study (Elite Double 32), Lee et al. [17] reported baseline thickness deviations of approximately $\pm$ 6.6% relative to the mean thickness. Previous studies have shown that such uncontrolled, axisymmetric thickness variations have a weak influence on the critical buckling pressure compared to intentionally introduced geometric imperfections [21]. More recent work further demonstrates that when multiple imperfections are present and sufficiently separated, the largest imperfection dominates the buckling response [26].

To ensure that this baseline effect does not influence our conclusions, we fabricated additional near-perfect shells without acoustic excitation using the identical experimental configuration. Measurements confirm that the polar thickness variation in these static shells remains within the same range reported in the literature and does not introduce significant non-axisymmetric features (see SI Fig. 7).

R1C7. the comparison in Fig. 4 is only visual. Why is there no quantification of results?

We thank the reviewer for this important point. We agree that a quantifiable comparison would further establish correspondence between the predicted and measured patterns. It is important to note, however, that the finite-element (FE) vibrational mode shapes of the mold represent displacement fields, whereas the experimentally measured imperfection fields of the cast shells represent spatial variations in cured thickness. With Fig. 4 of the manuscript, we aim to showcase that even though the two quantities are not directly equivalent, both reflect the same underlying pattern, suggesting one is correlated with the other.

Rather than computing a spatial correlation on a coordinate-level, we measure the fabrication reproduction of the features of the FE-predicted modes, both in symmetry and in spatial distribution. Specifically, we assess whether the defects are present at the antinodes of the modal shape.

In Abaqus, the eigenvectors are normalized such that the maximum of each displacement component is 1. The magnitude of displacement is visualized using greyscale where white and black correspond to zero and maximum displacements respectively. As a result of the above analysis on the fluidic motion, this greyscale value is squared.

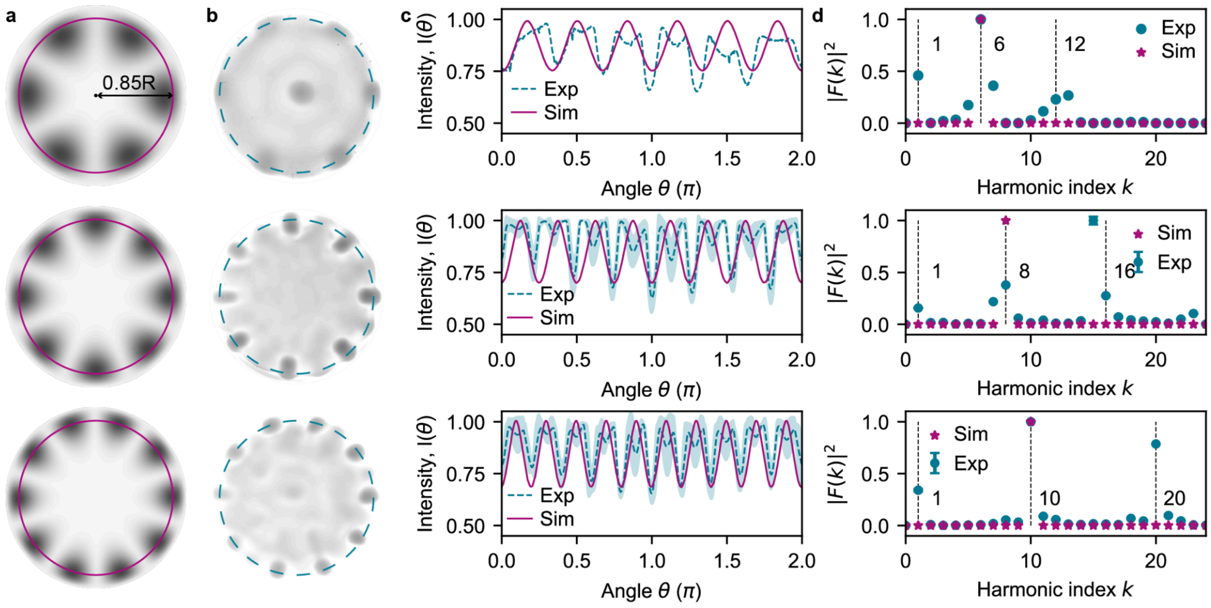

RFig 2. [SI Fig. 10] **Comparison between modal shapes from FE modal analysis and photographs of imperfect shells.** a. Three modal shapes showing 6, 8, and 10 antinodes in a ring near the perimeter where the greyscale represents normalized displacement magnitudes. b. Photographs of fabricated shells with 6, 8 and 10 bump-like imperfections. c. Intensity plots around a ring at a radius of 0.85R. d) Power spectra showing the magnitude of patterns with different periodicities.

We proceed to extract three modal shapes at the requested frequencies; these feature 6,8 and 10 bump-like defects respectively (RFig. 2a). The displacement fields resulting from FE modal analysis are plotted alongside the photographs of the imperfect shells (RFig. 2b). We plot the amplitudes of the intensity as a function of the azimuthal angle θ around a fixed radius (0.85R) to showcase the similarity between the two (RFig. 2c). The RMSE between experimental and simulation intensity measurements for the three specimens are 0.0798, 0.127, 0.0924 respectively. We further observe slight thickening at the nodes of the modal shape as well. To quantify periodicity, we analyze intensity variations using a Fourier transform. Each image is first reduced to a one-dimensional signal by averaging pixel intensities over a circular ring near the defects ($0.85 \pm 0.5R$), yielding an intensity profile $I(\theta)$. The profile is then mean-subtracted to remove the zero-frequency component and transformed using discrete Fourier transform.

The resulting Fourier coefficients decompose the signal into harmonics corresponding to periodic variations around the circumference. The magnitude of the coefficient of the power spectrum $|F(k)|^2$ at harmonic index k quantifies the strength of a pattern repeating k times over one revolution. When this is applied to the FE modal displacement, one clear peak is present, representing the number of antinodes in that particular mode (RFig. 2d). For the fabricated specimens, the power spectrum similarly shows a local maximum at the requested number of defects. However, other local maxima are also present, specifically, at k=1 and at higher harmonics (e.g., k=12 for the specimens with 6 bump-like defects). The harmonic index of 1 reflects a global imperfection where one side of the hemisphere is thicker than the other. This can be attributed to the imprecision when mounting the mold onto the speaker and the acoustic quality of the speaker itself. The higher harmonics arise from the non-sinusoidal intensity profiles. The signatures around the peaks can be attributed to nonlinearities in the acoustic forcing and geometrically nonlinear structural response at high excitation amplitudes. Altogether, these show that the imprinted imperfections can faithfully retain the feature of the natural vibrational shapes from FE modal analysis.

A full physical model linking acoustics to structural vibration to steady-state thickness redistribution is beyond the scope of the present work and will be developed in future studies.

This process is detailed in Appendix IV of this rebuttal and included in its entirety in the revised SI. We have also added the following in the main text of the manuscript:

Although the FE vibrational mode shapes represent displacement fields of the mold and the experimentally measured shell geometries represent thickness variations, both share the same underlying spatial symmetry and antinode distribution. To quantitatively assess this correspondence, we compare the circumferential intensity patterns of the FE-predicted modes and the fabricated shells along a circular ring near the perimeter (r = 0.85R). For each specimen, the intensity is averaged along this ring and plotted as a function of azimuthal angle. The resulting profiles exhibit matching periodicity between simulation and experiment, with root-mean-square errors of 0.0798, 0.127, and 0.0924 for the 6-, 8-, and 10-defect cases, respectively. See SI section 3.1 and SI Fig. 9 for detail.

To further quantify periodicity, we compute the discrete Fourier transform of the azimuthal intensity profiles. The resulting power spectra show dominant peaks at harmonic indices corresponding to the number of antinodes in the targeted vibrational mode, both in the FE predictions and in the fabricated shells (SI Fig. 10d). Additional lower-amplitude harmonics reflect global asymmetry and nonlinearities at higher excitation amplitudes.

R1C8. page 5: "These findings confirm that the acoustic excitation protocol introduces repeatable, spatially structured imperfections whose geometry closely resembles vibrational mode shapes." I could not find the proof of that in the paper.

The original statement referred to the reproducibility of the imperfection patterns across multiple shells fabricated under identical excitation conditions and to their qualitative resemblance to the predicted modal shapes. **R1C7** has shown feature resemblance between fabricated shapes and FE modal shape, here we show that the fabrication method is repeatable.

In addition to the three near-perfect shells fabricated in **R1C6**, we have further fabricated three shells with 8 bump-like defects. Multiple molds are used to strengthen the claim of repeatability. We photograph all 6 shells (3 near-perfect and 3 with 8 bumps) under identical conditions and calculate the Pearson Correlation coefficients (R.Eq. 1) between all pair combinations (15 in total),

$$p\left(I_1, I_2\right) = \frac{\sum_{i=1}^{N}\left(I_{1,i} - \bar{I}_1\right)\left(I_{2,i} - \bar{I}_2\right)}{\sqrt{\sum_{i=1}^{N}\left(I_{1,i} - \bar{I}_1\right)^2}\sqrt{\sum_{i=1}^{N}\left(I_{2,i} - \bar{I}_2\right)^2}}, \qquad \text{R.Eq. 1}$$

where I_1 and I_2 are the vectors of pixel intensities of two images being correlated, and N is the number of pixels within the circular mask of the photographs.

The results show that within each group (RFig. 3), the correlation means are $\bar{p}_0 = 0.936$ and $\bar{p}_8 = 0.638$ for near-perfect and 8 defects respectively, with $p = 1$ representing a perfect correlation. The deviations within each group can be attributed to three primary causes. The first is the time lapsed between silicone mixing and pouring. This delay influences the eventual thickness of the specimen (Lee, et al., Nature communications, 7(1):11155, 2016) and may explain the difference between t8-s3 and the remaining two. The second is the imprecision in positioning and leveling the mold on the speaker. The mold is secured onto a circular PMMA plate, which is bolted to the rim of the loudspeaker. In this setup, the mold may not be perfectly concentric to the diaphragm of the speaker. Depending on the tightness of the bolts, the lip of the mold may be asymmetrically compressed. Both errors may result in the specimen appearing asymmetric. The third is in image processing, specifically, we manually align one bump-like defect along the horizontal axis by rotating the image. Nevertheless, the within-group correlations are substantially higher than cross-group correlations, confirming that the fabrication process produces consistent imperfection patterns.

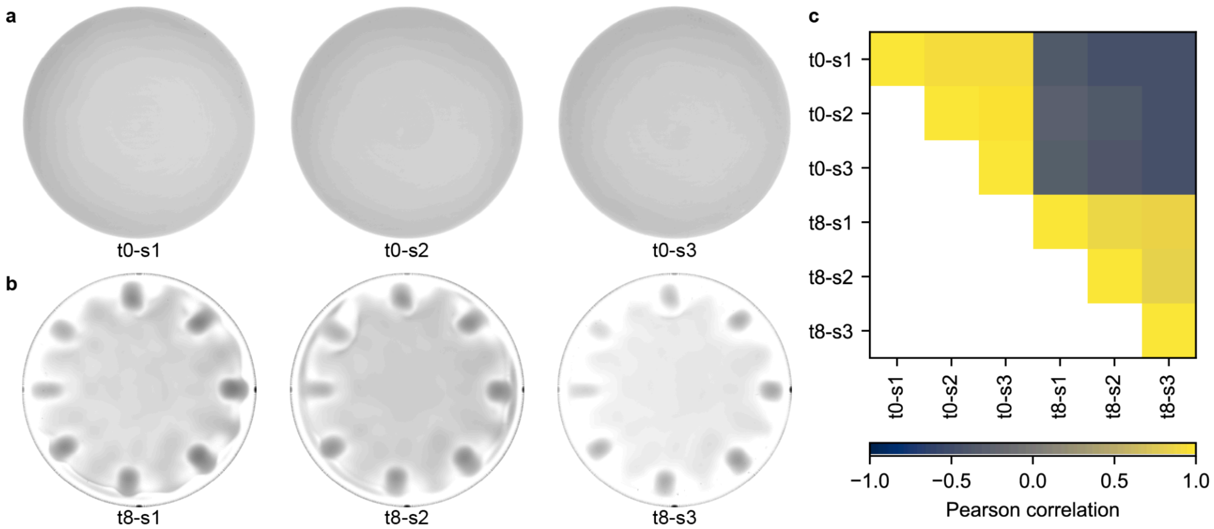

RFig. 3. [SI Fig. 3] **Shape comparison between shells based on photographic evidence.** a. Three near-perfect shells and b. three shells with 8 bump-like defects are compared. c. The resulting Pearson correlation is plotted showing close correlation between shells within each group, and minimal correlation across groups.

The above has been included in Appendix III of the rebuttal and reproduced in the revised SI. The following are included in the revised manuscript:

To assess repeatability, we fabricated multiple shells under identical excitation conditions and quantified the similarity of the resulting imperfection fields. Three near-perfect shells (no acoustic excitation) and three shells with eight bump-like defects were fabricated using multiple molds and photographed under identical conditions. Pairwise Pearson correlation coefficients were computed between the grayscale thickness maps of all specimens (see SI section 1.5 and SI Fig. 3). Within each group, the mean correlation coefficients are 0.936 for near-perfect shells and 0.638 for shells with eight defects, indicating consistent reproduction of the targeted pattern. In contrast, correlations across the two groups are negligible (≈-0.12).

R1C9. page 6: "under increasing vacuum pressure (Fig. 5)." This phrase sounds a bit awkward or ambiguous. Is the pressure increasing (less vacuum), or is the vacuum getting stronger (less pressure)? One can easily figure it out when learning about the buckling experiment with vacuuming the inner space of the shell, i.e. by applying the outside pressure (which is limited by the amount of the atmospheric pressure).

We agree that the phrase "increasing vacuum pressure" is unclear. In our experiments, the shells buckle as the internal pressure is gradually reduced, thereby increasing the net external pressure acting on the shell. We have revised the wording throughout the text to clearly describe this loading condition as "increasing external pressure by progressively reducing the internal pressure of the shell."

The modification is reproduced below:
Lastly, we quantify the mechanical response of this class of imperfect shells under increasing external pressure, achieved by progressively reducing the internal pressure of the shell (Fig. 5).

R1C10. page 6: "The stiffness of all shells remains relatively constant." What does this mean quantitatively? There is no proof of that given in the paper. (I hope I did not miss it.)

The statement refers to the pre-buckling stiffness, *i.e.*, the slope of the quasi-linear portion of the normalized pressure-volume (P-V) curves prior to the on-set of instability. We quantify this stiffness by computing $E_{norm} = p_{norm}/V_{norm}$ over the initial linear regime ($V_{norm} = 0.002$). This quantity (shown in the table below) is computed for 9 sets of specimens (three speaker frequency and three speaker volume variations), in addition to the specimen fabricated in absence of acoustic vibration.

RTable. 1. Linearized stiffness measure of the different shell specimens.

	No volume [-]	Volume 1 [-]	Volume 2 [-]	Volume 3 [-]
Near-perfect shell	119.7 ± 10.54			
6 bump-like defects		115.5 ± 14.09	115.1 ± 7.685	72.21 ± 12.61
8 bump-like defects		133.5 ± 9.091	112.1 ± 1.304	107.9 ± 8.599
10 bump-like defects		113.3 ± 8.810	109.6 ± 6.624	104.2 ± 3.334

The above results are plotted in an updated Fig. 5 of the manuscript (RFig. 4b), where it is observed that the stiffness measures for most shells are within the error-bounds of the near-perfect shell. The only deviation is the imperfect shells with 6 bump-like defects manufactured at the highest volume. It is hypothesized that large thickness defects invalidated the assumptions of thin-shell mechanics with "small" defects.

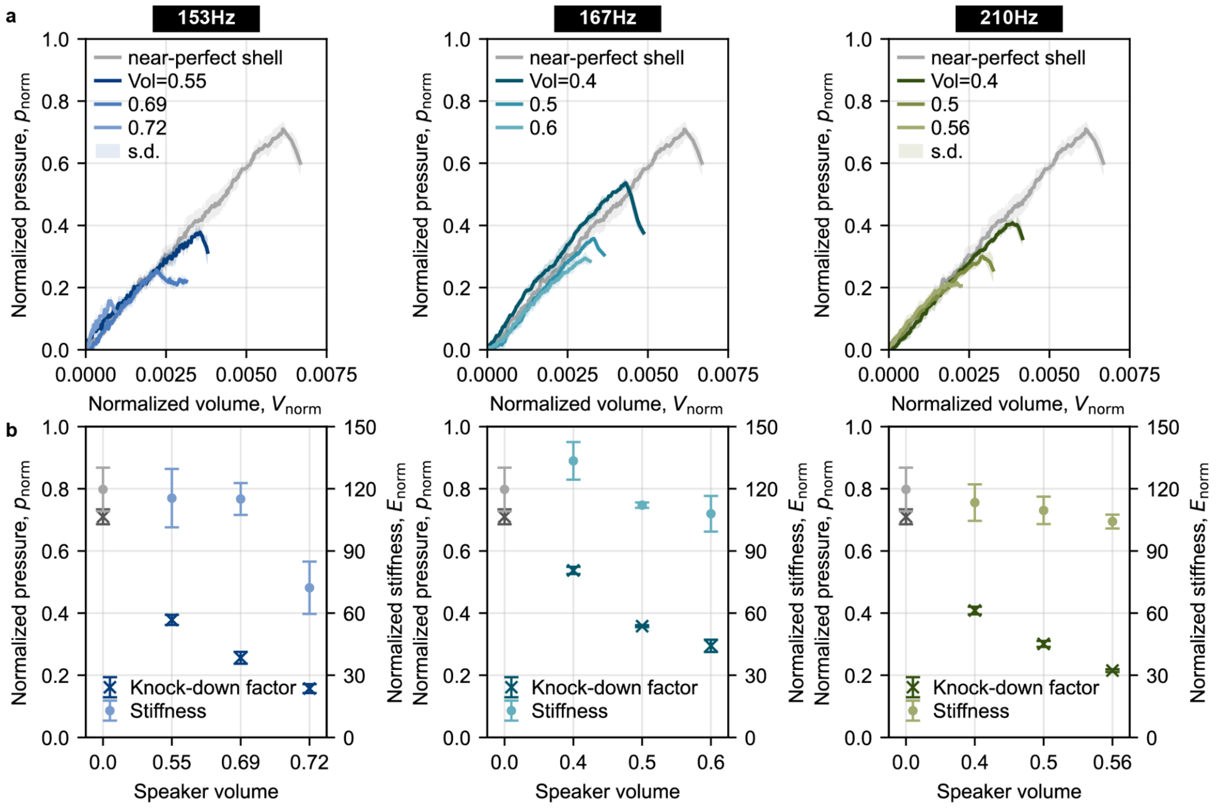

RFig. 4. [Manuscript Fig. 5] **The buckling response of hemispherical shells with vibration-induced imperfections.** a) Normalized pressure-volume curves for shells fabricated with specific speaker excitation volume and frequencies. Behavior of a shell with no vibration-induced imperfections is plotted for reference. b) Normalized pre-buckling stiffness E_{norm} and knock-down factors as a function of speaker volume, quantifying the reduction in structural stability with increasing imperfection severity.

In addition to the change to Fig. 5 of the manuscript, we have amended the text as follows,

We further quantify the stiffness as the slope of the quasi-linear portion of the normalized pressure-volume curves prior to the onset of instability (Fig. 5b). We compute a normalized stiffness as $E_{norm} = p_{norm}/V_{norm}$ over the initial linear regime ($V_{norm} = 0.002$). Across all excitation frequencies and volumes, the measured stiffness values remain within the experimental variability of the near-perfect shell, indicating that the imposed imperfections primarily affect the critical buckling pressure rather than the pre-buckling stiffness. A deviation is observed only for shells with six defects fabricated at the highest excitation volume, where large thickness variations likely violate the assumptions of thin-shell behavior. See SI Table 1 for numerical values.

The table RTable. 1 is reproduced in the SI.

R1C11. caption of Fig. 5: of of

Thank you, we have corrected this typo.

R1C12. "Quantitative measurements from μCT and optical imaging confirm the high fidelity of modal patterning, while mechanical buckling tests demonstrate a systematic reduction in structural capacity with increasing imperfection amplitude." How is the high fidelity confirmed?

The ambiguous phrase "high fidelity" now refers to the quantitative correspondence between the measured thickness imperfection fields and the FE-predicted vibrational mode shapes, as well as the consistency between shells fabricated under identical excitation conditions.
These two aspects are now explicitly demonstrated in our revisions addressing **R1C7** and **R1C8**. We have clarified this in the discussion of the manuscript:

Quantitative correlation analysis between the measured imperfection fields and FE-predicted vibrational modes demonstrates that the vibration-assisted patterning achieves high spatial fidelity and repeatability across shells fabricated under identical conditions. Mechanical buckling experiments demonstrate a systematic reduction in structural capacity with increasing imperfection amplitude.

R1C13. What is the range of achievable eigenmodes?

The range of experimentally achievable eigenmodes is governed by several coupled experimental factors:

1. **Speaker fidelity** – The subwoofer used in this work can accurately reproduce low frequencies in the 40–250 Hz range. Accessing higher-order modes would require a mid-range or tweeter-type acoustic driver capable of accurately generating vibrations above 500 Hz.

2. **Viscoelastic damping of the mold** – If the same amplitude of vibration is to be maintained at a higher frequency, the strain-rate in the material must necessarily increase. For a material that exhibits viscoelastic damping (such as the silicone we use), the strain energy must increase as well. This requires the power of the speaker to increase. Conversely, if the speaker power remains unchanged, the amplitude of vibration will decrease with higher frequencies until it cannot appreciably influence casting.
3. **Streaming flow penetration depth** – The secondary (streaming) flow that redistributes the uncured silicone penetrates a depth proportional to modal vibrational wavelength; at shorter distance between neighboring antinodes, the flow is confined near the surface of the mold and the magnitude of streaming force decreases.
4. **Resistance due to surface tension** – The surface tension force that resists deformation of the fluid-air interface is proportional to the square of the modal vibrational wavenumber. Higher-order modes are therefore strongly damped by surface tension.

As a result, the current setup reliably accesses the first three to four non-axisymmetric modal families (approximately 150–220 Hz). We have added a paragraph in the *Results* section clarifying these dependencies and limitations.

By modulating both frequency and volume, we are able to systematically change imperfection geometry and severity. In principle, we have access to all modes as predicted by FE, however, experimentally, physically achievable eigenmodes are governed by several coupled experimental factors. First, the subwoofer used in this study accurately reproduces low-frequency excitation (40–250Hz), while higher-order modes would require a mid-range or tweeter-type driver capable of generating vibrations above 500Hz. Second, because the silicone mold is viscoelastic, maintaining the same vibration amplitude at higher frequencies would incur greater strain energy, which requires input power. If the speaker output remains constant, the amplitude decreases with frequency until the vibration becomes insufficient to influence the liquid layer. Finally, the secondary streaming flow that drives material redistribution penetrates a depth proportional to the vibrational wavelength. As the distance between neighboring antinodes decreases for higher modes, this flow becomes confined near the surface of the silicone mold and its magnitude diminishes.

R1C14. (just out of curiosity) Does it make sense to use mixed modes?

Yes, by exciting the mold with simultaneous or sequential multi-frequency signals corresponding to multiple resonant modes, we can generate mixed-mode imperfection fields. Within a small-amplitude regime, we anticipate a superposition of modal displacement fields, and the resulting imperfection geometry would reflect this superposition. As the excitation amplitude increases, coupling between modes and the viscoelastic damping of the mold may cause one mode to dominate. Sequential excitation (switching between frequencies during curing) could result in coupling between the fluid accumulation and the kinetics of curing. A detailed analysis of this is outside of the scope of this work. However, we plan to use this fabrication method to create randomized imperfection fields. To this end, we have fabricated additional shells that are excited by randomized frequencies. Specifically, we play multiple songs representing a wide spectrum of genres and demonstrate the variety of different imperfection shapes we are able to obtain (RFig. 5).

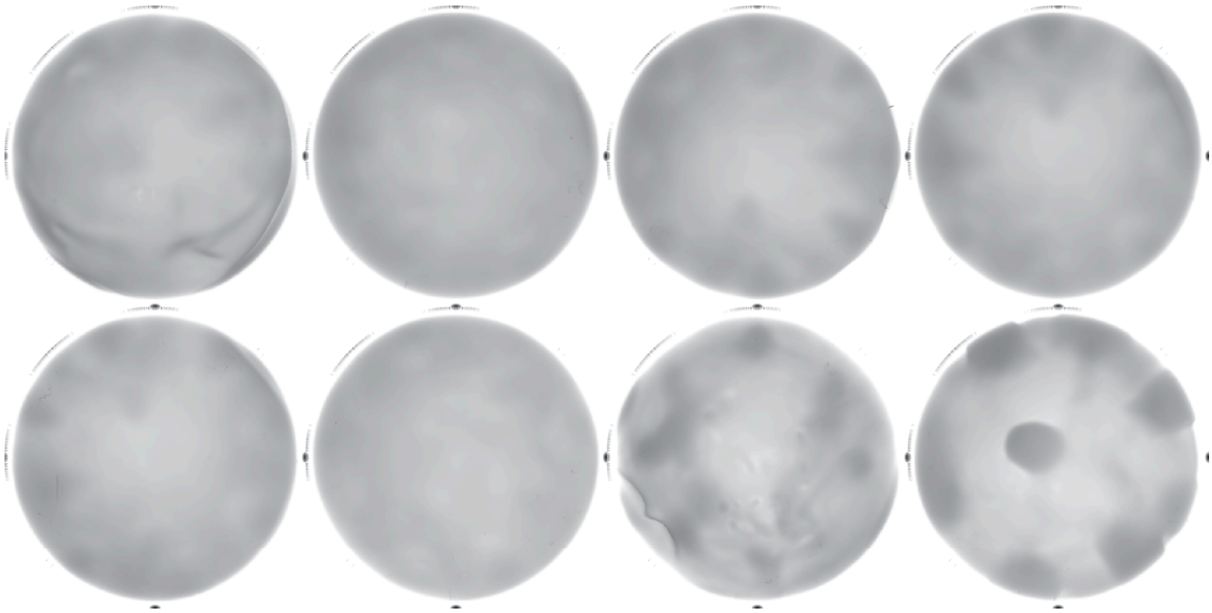

RFig. 5. [SI Fig. 4] **Mixed-mode imperfections.** Eight different shells fabricated using sequential broadband and time-varying excitation signals.

We have added the following to the discussion of the manuscript:

Beyond single-mode excitation, this fabrication method enables the generation of mixed-mode and non-periodic imperfection fields through sequential or broadband excitation. By exciting the mold with sequential multi-frequency signals, the resulting thickness variations reflect both superposed and emerging modal features. The resulting shell geometries exhibit both features resembling the single-mode patterns shown in Fig. 4 and previously unobserved spatial patterns. Representative examples and experimental details are provided in the SI section 1.6. The ability to imprint complex thickness landscapes highlights the flexibility of vibration-assisted patterning and suggests routes toward exploring the mechanics of imperfection interactions in thin shells.

We have included the following to the SI:

To demonstrate the ability of the fabrication method to generate mixed-mode and non-periodic imperfection fields, we fabricated additional shells under broadband and time-varying excitation conditions. Rather than applying single-frequency sinusoidal signals, the mold is driven using audio signals containing a wide range of frequencies and temporal modulations. This approach results in highly diverse, non-axisymmetric thickness patterns that differ qualitatively from the single-mode cases presented in the main text.

Representative examples include excitation using music spanning different genres and spectral contents, which serve here as convenient broadband inputs. The resulting imperfection fields exhibit both features resembling individual modal patterns and previously unobserved spatial organizations (SI Fig. 4).

R1C15. Was the effect of boundary conditions checked (I am referring to repeatability)? In other words, does something change if the silicone mold is demounted and mounted again? The boundary condition might change a little. Nevertheless, while the proposed experimental approach is intriguing, the work lacks at least some basic statistical characterization. The question on robustness /

repeatability remains open in my opinion, which is not good. The reproducibility of thickness, (very importantly) patterns and consequently knockdown factor measurements must be quantified. I recommend presenting the results in means +/- SD for thickness, imperfection amplitude and critical pressure over at least three independent samples per fixed set.

We agree with this point and believe we will have addressed it with the new results shown in **R1C8**. As described in that response, the repeat specimens were fabricated using different molds that were independently mounted onto the speaker, thereby capturing variability introduced by the boundary conditions.

R1C16. Technically, the speaker essentially induces structural vibration of the mold through the air volume inside the shell. The term "vibration-assisted" would therefore be far more precise. Please note, that no acoustic effects were found on the shell. This comment does/should not diminish the cleverness of the proposed method. Acoustic --> Vibration?

We agree that the physical mechanism in our setup is the structural vibration of the elastic mold. The use of a loudspeaker facilitates this vibration in a systematic way. We have revised the title of the paper to "Vibration-Assisted Fabrication of Thin Shells with Spatially Distributed Imperfections". We have also changed this phrasing throughout the manuscript.

R1C17. I recommend minor revisions before the manuscript can be considered for publication. These minor revisions can become major revisions if the authors did not save enough data for at least a basic statistical analysis.

Thank you very much for the insightful comments. I hope the extensive modifications we have made satisfactorily address the comments.

Reviewer 2

This manuscript introduces an interesting vibration-assisted casting method to imprint modal imperfections into thin shells. It could contribute to further work on imperfection-sensitive buckling and could find broader applications in soft matter engineering. However, in my opinion there are some conceptual points that need to be further clarified, and more importantly, although the authors propose this clever manufacturing procedure, no real advance is obtained in the understanding and prediction of buckling properties of architectured or defected shells. Therefore, I am not convinced that the work entails a sufficiently important breakthrough ("to advance understanding in a way that will move the field forward") to be published in Nature Communications.

We thank the reviewer for their comments. We agree that the primary contribution of this work is not the development of a new predictive theory for the buckling of imperfect shells. Rather, the central advance lies in the introduction of a scalable, tunable, and repeatable fabrication platform that enables the controlled imprinting of global, spatially distributed imperfection fields whose geometry is governed by well-defined vibrational modes.

We hope that by addressing the following comments, we can convince the reviewer of their concerns.

Main Comments:
R2C1. The manuscript refers to the acoustically imprinted thickness variations as "imperfections", but the patterns are deliberately engineered, so it would be more appropriate to speak of "architectured features". I think the term "imperfections" is potentially misleading and inconsistent with prior literature.

We agree that the thickness variations introduced in this work are deliberately engineered rather than unintended manufacturing defects. However, we use the term *"imperfections"* intentionally and in a context-specific sense that is consistent with the shell buckling literature.

In classical and modern shell mechanics, the term *geometric imperfection* refers broadly to any deviation from the idealized, perfectly axisymmetric shell geometry assumed in theoretical buckling analyses, regardless of whether that deviation arises unintentionally or is deliberately introduced for experimental control. Numerous prior studies explicitly employ intentionally prescribed dimples, thickness variations, or defect fields as *controlled imperfections* to probe imperfection sensitivity (e.g., Lee et al., Marthelot et al., Abbasi et al., see full list below).

In this work, the vibration imprinted thickness variations serve precisely this role: they are engineered deviations from the perfect shell used to interrogate imperfection-sensitive buckling behavior. To avoid ambiguity, we have revised the manuscript to explicitly clarify this definition at first use. Further, the reviewer brings up an interesting point where architected features may potentially enhance the buckling capacity.

The following is included in the introduction of the manuscript:

In these works, the term "imperfections" is used in the shell mechanics sense to denote deviations from an idealized perfect geometry, including deliberately prescribed features introduced to probe imperfection-sensitive buckling behavior.

References:

- Derveni, F., Choquart, F., Abbasi, A., Yan, D., & Reis, P. M. (2025). The most severe imperfection governs the buckling strength of pressurized multi-defect hemispherical shells. Mechanics of Materials, 204, 105295.
- Derveni, F., Abbasi, A., & Reis, P. M. (2025). Defect–Defect Interactions in the Buckling of Imperfect Spherical Shells. Journal of Applied Mechanics, 92(4), 041003.
- Derveni, F., Gueissaz, W., Yan, D., & Reis, P. M. (2023). Probabilistic buckling of imperfect hemispherical shells containing a distribution of defects. Philosophical Transactions of the Royal Society A, 381(2244), 20220298.
- Abbasi, A., Yan, D., & Reis, P. M. (2021). Probing the buckling of pressurized spherical shells. Journal of the Mechanics and Physics of Solids, 155, 104545.
- Lee, A., López Jiménez, F., Marthelot, J., Hutchinson, J. W., & Reis, P. M. (2016). The geometric role of precisely engineered imperfections on the critical buckling load of spherical elastic shells. Journal of Applied Mechanics, 83(11), 111005.

R2C2. Although the experiments are well-conceived and explained, and good agreement is found with FE modal analysis, they would benefit from additional control experiments to provide a quantitative baseline (i.e., casting without acoustic excitation). For example, it would be useful to have data on shell thickness uniformity in the absence of vibration.

We thank the reviewer for raising this important point. This suggestion is also raised by Reviewer 1 in **R1C6**. We fully agree that a quantitative baseline obtained from casting without acoustic excitation is essential. In brief, we performed static casting experiments without acoustic excitation to quantify baseline thickness uniformity and verify the absence of non-axisymmetric features. The results are consistent with literature (Lee, et al., Journal of Applied Mechanics, 83(11), 111005, 2017). These are now described in the Results section and included in the Supporting Information. We refer the reviewer to the detailed response under Reviewer 1, Comment **R1C6** as well as Appendix II of this rebuttal.

R2C3. The authors state that "the accumulations coincide with the locations of the antinodes (Fig. 2d). This seems counterintuitive as the antinodes experience the largest vibration amplitude and could cause the liquid to flow away". This point would require more discussion and analysis. The explanation of how and why material accumulates at antinodes is only qualitative and , as the authors indicate, somewhat counterintuitive. The reference to secondary streaming is plausible but not backed up with flow measurements or simulations. A simplified quantitative model might be useful, or at least reference to detailed hydrodynamic theory, to support this behaviour.

We thank the reviewer for this important suggestion. We have developed a quantitative analysis of the vibration-driven flow, presented in full in Appendix I of this rebuttal and included in the revised SI (Section 5).

We perform a weakly nonlinear analysis of a liquid layer driven by a vibrating lower boundary, using a perturbation expansion in the dimensionless vibration amplitude $\epsilon^* = \epsilon/H$, following approaches used in related vibrating-boundary problems (e.g., Hoepffner & Fukagata, *JFM*, 2009). The fluid is treated as Newtonian, consistent with the weakly shear-dependent rheology of the silicone used in our experiments.

At leading order, the vibration of the mold drives a time-periodic circulating primary flow with the same frequency and wavenumber as the mold's vibration pattern. Because this flow is sinusoidal in both space and time, it produces no mean deformation of the free surface.

At second order, the primary flow generates non-zero Reynolds stresses with twice the wavenumber of the vibration. These Reynolds stresses, together with an inhomogeneous boundary forcing arising from the primary flow, drive a steady secondary streaming flow. Using physical and symmetry arguments, we show that the pressure associated with this streaming flow is elevated above the antinodes of the mold's vibration and reduced above the nodes. If the free surface is allowed to deform, it deflects outward at the antinodes — that is, fluid accumulates at the antinodes, exactly as observed in our experiments.

The analysis also yields a key quantitative prediction: the free surface deflection has twice the wavenumber of the mold vibration, and its dimensional amplitude scales as $A \sim \lambda^2 \mu^2 \omega^2 \epsilon^2 \gamma^{-2} H^{-1}$, where λ the wavelength, μ the fluid's viscosity, ω the vibration frequency, ϵ the vibration amplitude, γ the surface tension, and H the liquid layer thickness.

These theoretical predictions are consistent with our experimental observations. The particle tracking experiments (Fig. 2e) directly visualize the progressive migration of material toward the antinodes driven by the secondary streaming flow, and the resulting thickness fields (Figs. 3–4) confirm that the spatial periodicity of the imperfections matches the predicted doubled wavenumber of the streaming flow. We note that this same physical mechanism was independently identified as a concern by Reviewers 3 and 4 (see R3C4 and R4C2), and the analysis presented here addresses all three comments.

The following text has been added to the manuscript:

A theoretical analysis of the flow reveals that the mold's vibrations drive an oscillatory primary flow of the same frequency and wavelength as the vibrations. The primary flow creates Reynolds stresses with half the wavelength that, together with a boundary forcing created by the primary flow, drive a secondary, time-averaged, streaming flow with half the wavelength. The associated pressure would push an undeformed free surface outward at the locations of the antinodes, leading the liquid silicone to redistribute toward the antinodes. See SI section 5 for more details.

R2C4. The role of material properties is not discussed in the mechanism, e.g. elasticity, viscosity, rheology of the silicone during curing, curing time vs flow time. It would be important to try to analyse quantitatively how material properties influence the final shell shape.

We thank the reviewer for raising this important point. We agree that material properties such as viscosity, elasticity, and curing kinetics influence vibration-assisted casting. In our system, however, the formation of the thickness pattern occurs on a timescale that is well separated from curing and solidification. Specifically, redistribution of the uncured silicone and the formation of the thickness variations occur within the first 1 to 5s after excitation, whereas the curing time of the silicone is approximately 20 min. As a result, the shell geometry is established while the viscosity of the material remains relatively constant (Lee et al., Nature Communications, 2016). The chemistry of curing primarily serves to freeze in an already-formed thickness distribution.

The rheological properties of the silicone used in this study (Elite Double 32) have been characterized in detail in literature, including viscosity, coating dynamics, and the evolution of elastic modulus during curing (Lee et al., Nature Communications, 2016). In the regime relevant here, the material behaves as a Newtonian fluid and does not exhibit shear-thickening or shear-thinning behavior.

Using a silicone with significantly different viscosity or surface tension would alter the streaming flow magnitude and the balance between redistribution and curing, potentially changing the achievable imperfection amplitude. The scaling law derived in SI Section 5 (included in Appendix I of this rebuttal) provides a quantitative framework for predicting these dependencies.

To clarify this mechanism, we have added a discussion of timescale separation and material behavior to the Methods section and replicated below.

Further, the exponential nature of curing suggests that, during the initial period following pouring, the silicone behaves predominantly as a viscous Newtonian fluid and does not exhibit shear-thickening or shear-thinning behavior [17,35].

The strong separation of timescales between vibration-driven redistribution (1 to 5 seconds) and curing (20 minutes) implies that the thickness patterns form well before solidification. The final shell geometry is therefore established during the liquid phase, while curing primarily serves to freeze an already-formed thickness distribution. Accordingly, the observed pattern formation is governed by vibration-induced flow.

R2C5. The match between FE modal analyses and measured shell geometries is mostly qualitative. A more rigorous quantitative comparison would strengthen this connection. It would also be interesting to see if more complex architectured structures can be created, e.g. by exciting at multiple frequencies and exploiting the superposition of modes.

We thank the reviewer for this comment. Both points raised here overlap with concerns independently raised by Reviewer 1 in **R1C7** and **R1C14**.

Regarding quantitative comparison between FE modal shapes and experimentally measured shell geometries, we now provide a quantitative comparison based on circumferential intensity profiles extracted from both FE-predicted displacement fields and experimentally measured thickness maps. Root-mean-square errors and Fourier spectral analyses are used to quantify agreement in spatial periodicity and symmetry. Please see our response to **R1C7** as well as Appendix IV of this rebuttal for a thorough discussion of this point. These additions have also been explained in the main text and incorporated into a new SI section 3.1.

Regarding the creation of more complex architectured structures via multi-frequency excitation and modal superposition, this point is addressed in response to Comment **R1C14**. We now clarify in the Discussion that sequential and broadband excitation can generate mixed-mode imperfection fields. Representative examples using time-varying excitation are provided in the SI section 1.6 (see response to **R1C14**), demonstrating the flexibility of the fabrication approach, while a systematic analysis of modal superposition is left for future work.

R2C6. As mentioned, the authors propose a very clever manufacturing procedure to introduce architectured features in a hemispherical shell, demonstrating that the presence of these is correlated

with the knock-down factor of the structure (which is not surprising). The correlation with speaker volume is only phenomenological, and can be used for practical manufacturing applications, but does not contribute to a deeper understanding of the buckling of defected hemispherical shells. To achieve broader impact, the authors might try to suggest more specific applications in programmable materials and soft robotics, as they are generically mentioned only briefly. A demonstration, prototype, or at least a more detailed conceptual framework for these applications would enhance the significance of the work.

We thank the reviewer for this thoughtful and constructive comment. We agree that the correlation between speaker volume and knock-down factor is phenomenological, and we do not claim that it constitutes a new predictive theory for the buckling of defected shells. Rather, a central contribution of this work is the introduction of a fabrication platform that enables the controlled, repeatable, and scalable imprinting of global, spatially distributed imperfections that are otherwise difficult to realize experimentally. While the resulting reduction in buckling load with increasing imperfection amplitude is consistent with classical understanding of imperfection sensitivity, the novelty lies in the ability to engineer imperfection fields that follow prescribed modal symmetries and to tune their severity continuously. This capability enables systematic experimental access to classes of imperfection geometries that bridge idealized theoretical models and the complex, distributed defect fields encountered in practice.

We agree with the reviewer that articulating broader implications beyond shell buckling strengthens the impact of the work. Here, we reframe the imposed imperfections as architected features that may enable novel applications in programmable materials, morphable structures, and soft robotics. In particular, vibration-assisted patterning may be used to encode deformation pathways, bias snap-through locations, and localize compliance in thin soft structures-functional attributes that are increasingly exploited in instability-enabled actuation and shape-morphing systems. While a full device-level demonstration or robotic prototype is beyond the scope of the present study, the added discussion situates the fabrication method within a broader class of geometry-programmed soft matter systems and highlights concrete directions for future application-driven work. We believe this framing more clearly communicates the broader relevance and enabling nature of the proposed approach.

While the present work focuses on hemispherical shells as a canonical system for imperfection-sensitive buckling, the underlying fabrication method is readily extendable to programming the mechanics of other soft material systems [49, 50]. Here, we reinterpret thickness imperfections as architected features, and leverage the spatially distributed nature of such features as a design variable. Such features may be used in localizing compliance, biasing snapthrough behavior, or encoding preferred postbuckling modes [38,39].

In programmable materials and soft robotics, geometric instabilities are increasingly exploited to achieve large, rapid shape changes, mechanical logic, or passive actuation without embedded electronics [40–42]. Examples include morphable shells that snap between configurations, soft robotic grippers that close via elastic instability, and surfaces that reversibly change curvature under pressure [43–45]. The ability to imprint global, mode-shaped thickness fields provides a means to pre-program these behaviors directly into the material geometry, rather than relying on external constraints or active control. In particular, recent studies have shown that spatial disorder itself can be treated as a programmable design variable, enabling tunable mechanical response and emergent behavior in elastic systems [46–48].

In conclusion, this study establishes vibration-assisted fabrication as an enabling platform for experimentally accessing distributed, tunable geometric features that reflect the complex defect landscapes encountered in real-world structures.

Minor details:
R2C7. Page 1: “Yet, it remains an active research topic on what “physically realistic” geometric imperfections most severely reduce the knockdown factor”. This phrase is unclear and somewhat awkward.

We have amended this sentence to the following and updated the manuscript.

While recent works have begun investigating multiple interacting imperfections [25, 26], it remains difficult for such fabrication methods to reproduce the complex, global imperfection fields that arise naturally from manufacturing variability. Moreover, constructing dedicated fabrication setups for all possible imperfection geometries is impractical.

R2C8. Page 3: “between successive coat” should be “between successive coatings”

This has been corrected.

R2C9. Page 3: “Once we achieve a mold thickness that exceeds the thickness of the eventual hemispherical shells by an order of magnitude”: why an order of magnitude?

When the mold is sufficiently thick relative to the liquid layer, its vibrational response is dominated by its own elastic properties rather than by fluid-structure coupling with the uncured silicone. This ensures that the vibrational mode shapes and frequencies of the mold remain well-defined, stable, and reproducible during casting. We have included the following in the manuscript:

This thickness ratio ensures that the vibrational response of the mold is dominated by its own elastic properties and is minimally influenced by fluid-structure coupling with the yet-uncured silicone layer.

R2C10. Page 3: “The frequency and volume of speaker is digitally…” should be “The frequency and volume of the speaker is digitally…”

This has been changed.

R2C11. Page 3: “(frame rate equally to the frequency of the speaker)” should be “(frame rate equal to the frequency of the speaker)”

This has been changed.

R2C12. Page 4: “A laser sheet is projected across the shell of radius r at a given elevation, $z = 0.25r$, to highlights the location of nodes and antinodes by recording the distortions along the intersection between the elastic mold and the laser (Fig. 2b).” . It is not clear what is being measured and how. Also, “highlights” should be “highlight”. Finally , reference to Fig. 2b does not match what is shown. Please revise.

We thank the reviewer for pointing out this lack of clarity. In this measurement, a thin laser sheet forms a planar light "slice" that intersects the surface of the mold or of the shell as they are being acoustically vibrated. The intersection appears as a bright curve that can be optically recorded using an overhead camera in an otherwise dark room (RFig. 6c). Since the shutter speed of the video camera is much slower than the frequency of the speaker, all captured images are "time-integrated". As the mold vibrates, the points that undergo larger out-of-plane motion produce a visibly larger illuminated region, the points that do not remain a thin curve. This can be seen in Fig 2b,d of the manuscript.

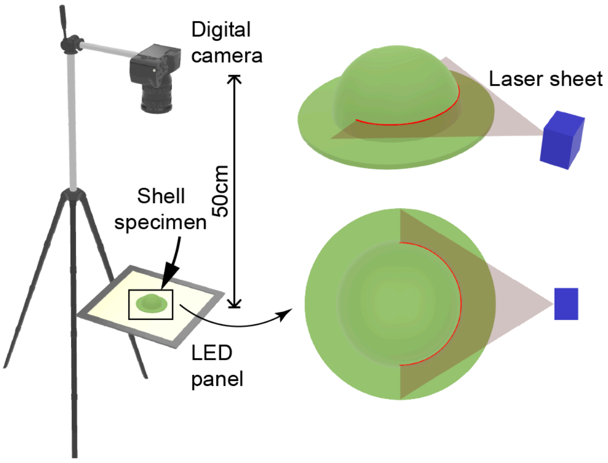

RFig. 6. [SI Fig. 6c] Photograph setup using a laser sheet to capture vibrational characteristics.

We use the recordings of the mold to identify the location of the nodes where there is minimal motion and the location of the antinodes where there is maximum motion (manuscript Fig 2b). We then pour liquid silicone onto the mold and continue recording. There, we observe that material accumulation occurs at the locations of antinodes of the mold (manuscript Fig 2d).

We have revised the manuscript to (i) describe explicitly what is measured (the motion of the laser-surface intersection curve), (ii) correct the grammar ("highlight" instead of "highlights"), (iii) revise Fig. 2's caption so that it clearly depicts the laser-sheet intersection and how nodal/antinodal locations are inferred, and (iv) included the above figure in the SI Fig. 6c.

These changes are reproduced below:

To experimentally identify nodal and antinodal locations, we illuminate the vibrating mold with a laser sheet positioned at a fixed elevation $z=0.25r$. The laser sheet intersects the mold surface along a curve; we record the motion of this intersection curve around the circumference using time-integrated imaging. Locations with minimal motion are identified as nodes, whereas locations with maximal motion are identified as antinodes (Fig.2b).

R2C13. Page 4: "The same laser sheet photography from the top view shows that the accumulations coincide with the locations of the antinodes". Is there also a grading effect in the thickness as you move away from the antinode location?

We thank the reviewer for this insightful question. Yes, the thickness variations induced by vibration-driven flow are not localized as point-like accumulations, but instead form spatially graded thickness fields that decay away from the antinodal locations.

The laser-sheet visualization qualitatively identifies the spatial correspondence between regions of material accumulation and the antinodes of the mold vibration. Quantitative characterization of the graded thickness field is obtained independently through destructive thickness measurements and optical photography. One representative example is shown in RFig. 7, where the experimentally measured thickness profile exhibits a smooth decay away from the antinode and closely follows the spatial envelope of the FE-predicted modal displacement. Details of the image analysis are provided in Appendix II of this rebuttal and are included in the revised SI.

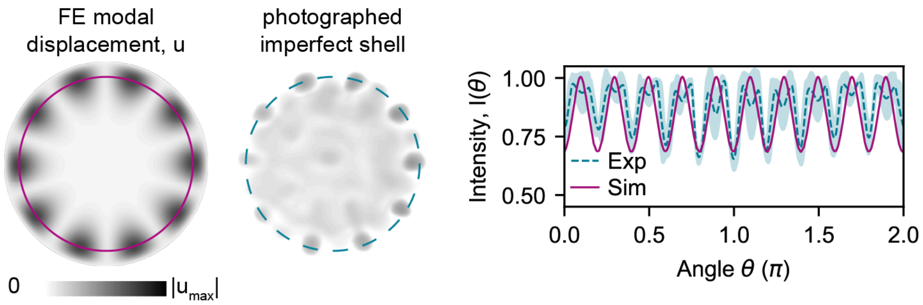

RFig 7. [Part of SI Fig. 10] Correspondence between modal displacement and the imperfect shell as photographed.

The following has been added to the results section of the manuscript:
The thickness profiles exhibit smooth, spatially graded variations that decay away from the antinodal peaks. This graded character is consistent with the spatial envelope of the FE-predicted modal displacement field.

R2C14. Page 4: "This secondary streaming flow is much weaker than the primary oscillatory flow, and bears resemblance to the streaming flow associated with Faraday waves": doesn't really explain why the material is accumulating at the antinodes, please expand.

This point is addressed in response to Comment **R2C3**. In brief, the weakly nonlinear analysis developed in Appendix I shows that the primary oscillatory flow generates Reynolds stresses that drive a steady secondary streaming flow, whose associated pressure field is elevated at the antinodes, causing the free surface to deflect outward and material to accumulate there.

R2C15. Page 4, section on flow dynamics: consider adding more description of the observed toroidal circulation in Fig. 2e. Does this circulation contribute to the steady-state accumulation pattern?

The steady accumulation pattern is created by the flow of the liquid silicone, and is associated with its secondary streaming component, as detailed in the weakly nonlinear analysis developed in Appendix I of this rebuttal (SI Section 5). The toroidal circulation visible in Fig. 2f — where material moves toward the circumference of each bump before recirculating under and emerging at the apex — is consistent with the steady streaming cells predicted by this analysis. These circulation cells are the physical manifestation of the secondary streaming flow that drives material toward the antinodes and maintains the accumulation pattern. The primary oscillatory flow alone would not produce net material transport; it is the nonlinear interaction through Reynolds stresses and boundary forcing that generates the steady circulatory pattern observed experimentally.

We note that gravity, which is absent from the 2D model in Appendix I, may bias the symmetry of the toroidal circulation by preferentially driving material downward along the outer surface of each bump and upward through the interior, consistent with the directionality observed in Fig. 2e. This is noted in the revised manuscript: "Here, gravity may bias the overall symmetry of the flow by acting as a body force on the accumulating fluid."

R2C16. Page 5: "…with changing its characteristics." should be "by changing its characteristics."

This has been changed.

R2C17. Page 6: "Full-field non-linear dynamical analysis will be conducted in the future to quantitatively construct this correlation". Why not in this work? It would increase its interest and impact

We thank the reviewer for this important question. With the revised text, we address the underlying mechanisms individually. First, we provide an analysis of the vibration-driven fluid motion and demonstrate the existence of a secondary (streaming) flow that drives material accumulation at regions of large vibration amplitude. This is included in Appendix I and in the SI Section 5. Second, the synchronization between the mold vibration and the excitation frequency of the acoustic driver is established experimentally through stroboscopic imaging. Third, the curing kinetics of the silicone are decoupled from the pattern-formation process through a clear separation of timescales, showing that the thickness field is established during the liquid phase and subsequently cured. Lastly, Appendix IV of this rebuttal constructs a quantitative correlation between the FE-predicted vibrational mode shapes and the measured shell geometries. It demonstrates that the spatial distribution of shell thickness can be inferred directly from the modal displacement fields. Together, these analyses establish a consistent experimental and phenomenological link between excitation, vibration, flow, and final geometry. Developing a single full-field nonlinear dynamical model that simultaneously couples large-amplitude structural vibration, oscillatory and streaming flow in a thin liquid layer, free-surface evolution, and time-dependent curing rheology would require solving a coupled multiphysics problem with significant modeling assumptions and parameter calibration. Such a formulation would constitute a substantial theoretical effort in its own right and is beyond the scope of the present study.

The primary contribution of this work is experimental: we introduce and validate a fabrication platform that enables controlled, repeatable imprinting of spatially distributed, mode-shaped thickness fields. By establishing experimental access to these imperfection geometries and quantifying their mechanics, the present study provides a foundation upon which future theoretical models can be built.

We have included this in the manuscript and reproduced below:

A fully predictive description that couples acoustic excitation, structural vibration, oscillation-driven flow, and curing kinetics will be conducted in the future to quantitatively predict this correlation. [..] To explore the underlying mechanism, we address the following physical processes. Analysis of the vibration-driven fluid motion reveals a secondary streaming flow that drives material accumulation at regions of large vibration amplitude.

R2C18. Page 6: "Increasing the speaker volume results in a systematic reduction in critical buckling pressure and earlier onset of instability." This is not surprising, given the cited literature. It would be more remarkable to gain a quantitative prediction of this reduction.

We wish to clarify the statement. While it is well established that larger geometric imperfections reduce the critical buckling pressure of thin shells, it is not immediately obvious that increasing the speaker volume in our system would produce a systematic and monotonic increase in imperfection magnitude, *i.e.*, without changing the shape of the imperfection. The relationship between excitation amplitude and final shell geometry is mediated by a strongly coupled multiphysics process involving structural vibration of a viscoelastic mold, oscillatory and streaming flow in the thin liquid layer, free-surface evolution under gravity, and curing kinetics. In such systems, nonlinearities, modal coupling, or flow instabilities could in principle produce entirely different pattern formation characteristics as excitation amplitude increases. Therefore, the observation that increasing speaker volume yields a controlled and reproducible increase in thickness variation amplitude is not trivial.

To quantify this relationship, we fit exponential decay curves to the measured knockdown factors as a function of speaker volume. The knockdown factor is modeled as $\kappa = \alpha\, exp(-\ k \cdot v)$, where v is the normalized speaker volume, α is a shared pre-factor, and k is a mode-dependent decay constant. Across all three modal families, the data are well described by a common pre-factor $\alpha = 0.7$, with decay constants $k_6 = 0.532$, $k_8 = 0.301$, and $k_{10} = 0.437$ for shells with 6, 8, and 10 bump-like defects respectively. The fits yield R^2 values of 0.991, 0.988, and 0.980 respectively, confirming the quality of the exponential model. These fits are overlaid on the knockdown factor data in the revised Fig. 5b.

The shared pre-factor across all three modes suggests that the baseline knockdown factor at low excitation is mode-independent, while the differing decay constants reflect the mode-dependent sensitivity of the buckling response to increasing imperfection amplitude. The faster decay for the 6-bump case is consistent with the observation that these shells exhibit the largest thickness variations at high speaker volumes, where thin-shell assumptions begin to break down (see **R1C10**). The scaling law from the streaming flow analysis (Appendix I, SI Section 5) predicts a quadratic dependence of imperfection amplitude on vibration amplitude, providing a theoretical basis for the observed exponential reduction in knockdown factor with increasing excitation.

These fits and the associated discussion have been added to the revised manuscript and SI.

To Manuscript:
To quantify the relationship between excitation amplitude and structural capacity, we fit exponential decay curves of the form $\kappa = \alpha\, exp(-\ k \cdot v)$ to the measured knockdown factors, where v is the normalized speaker volume. Across all three modal families, the data are well described by a shared pre-factor $\alpha = 0.7$, with mode-dependent decay constants $k_6 = 0.532$, $k_8 = 0.301$, and $k_{10} = 0.437$ ($R^2 = 0.991,\ 0.988,\ 0.980$ respectively). The shared pre-factor suggests that the baseline knockdown factor at low excitation is mode-independent, while the differing decay constants reflect the mode-dependent sensitivity of the buckling response to increasing imperfection severity. These fits are shown in Fig. 5b.

To SI Section 4.4:
To quantify the reduction in structural capacity with increasing excitation amplitude, we fit the measured knockdown factors to an exponential decay model $\kappa = \alpha\, exp(-\ k \cdot v)$, where v is the normalized speaker volume, α is a shared pre-factor common to all modal families, and k is a

mode-dependent decay constant. The fitting is performed using nonlinear least-squares regression (scipy.optimize.curve_fit).

R2C19. Page 7: "This technique opens new opportunities for studying multi-mode and nonlinear imperfection interactions, as well as programmable buckling responses." This work probably provides an additional tool to study these aspects, but does not address them directly. It would gain considerably in interest if it did.

We agree with the reviewer that the present work does not directly address multi-mode imperfection interactions or programmable buckling responses, and we have revised the manuscript to avoid overclaiming in this regard. However, we believe the work provides concrete first steps in several directions that collectively demonstrate the breadth of the platform.

Regarding multi-mode interactions, as described in our response to **R1C14**, we have fabricated additional shells under broadband and sequential multi-frequency excitation, demonstrating that the method can generate mixed-mode and non-periodic imperfection fields. Representative examples are provided in SI Section 1.6. While a systematic study of how these mixed-mode imperfection fields interact to influence buckling is beyond the scope of this work, these preliminary results demonstrate that the fabrication platform is capable of producing the complex imperfection geometries needed for such studies.

Regarding non-standard imperfection geometries, as described in our response to **R4C3**, we have demonstrated that by pre-designing the mold geometry with polygonal features, the vibrational response can be modified to produce imperfection patterns that break the symmetry of standard eigenmodes, including configurations with odd numbers of defects (SI Section 1.7). This further expands the accessible design space beyond what natural modal shapes alone can provide.

Regarding programmable buckling responses, as discussed in our response to **R2C6**, we have expanded the Discussion to articulate specific mechanisms by which mode-shaped thickness fields could be exploited as functional design variables — including localizing compliance, biasing snap-through behavior, and encoding preferred postbuckling configurations.

We have revised the relevant sentence in the manuscript to more accurately reflect the current scope of the work:

In conclusion, this study establishes vibration-assisted fabrication as an enabling platform for experimentally accessing distributed, tunable geometric features that reflect the complex defect landscapes encountered in real-world structures.

Reviewer 3

Nature Communications manuscript NCOMMS-25-50095
Paper Title: Acoustic-Assisted Fabrication of Thin Shells with Spatially Distributed Imperfections
Authors: I. Krida, et al.

Review Report:
In this manuscript, the authors present an acoustic-assisted method for fabricating thin shells with spatially distributed, vibrational mode-shaped imperfections. Through experiments, they demonstrate that the geometry of these imperfections can be customized by tuning the acoustic frequency and intensity of a standing wave. The authors further suggest that this technique could enable new opportunities in the development of morphable surfaces, acoustically controlled soft robots, and artificial shells with programmed buckling behavior.
However, I regret to say that I do not find this work suitable for publication in a high-impact journal such as Nature Communications. The manuscript lacks sufficient novelty, originality, and scientific depth. The proposed method is straightforward and does not demonstrate significant scientific rigor.

We thank the reviewer for their critical comments. We address the two comments separately. First, we have significantly expanded the manuscript to convince the review of the work's novelty, originality and scientific depth. Specifically, we have added (i) a weakly nonlinear analysis of the vibration-driven flow mechanism (SI Section 5), (ii) quantitative comparison between FE-predicted modes and fabricated shells via RMSE and Fourier spectral analysis (SI Section 3.1), (iii) statistical repeatability characterization across multiple molds (SI Section 1.5), (iv) baseline control experiments without acoustic excitation (SI Section 2.1), and (v) demonstration of mixed-mode imperfection fields (SI Section 2.3). However, we do claim that the "straightforwardness" of the method is one of its advantages.

R3C1. Research Motivation: The motivation for this research is not clearly articulated. It is unclear why the artificial introduction of imperfections into thin shell structures is necessary or significant. While the authors briefly mention potential applications related to buckling (in Discussion), the overall motivation remains vague, making it difficult for readers to appreciate the importance of the work.

The deliberate introduction of geometric imperfections into thin shells serves two distinct purposes. The first is scientific: imperfection sensitivity has been one of the central challenges in shell mechanics for over a century, yet experimental progress has historically lagged behind theoretical and computational developments. The classical buckling pressure of a perfect shell (Zoelly, 1915) may overpredict the load-bearing capacity by a factor of five or more, and Koiter's asymptotic theory demonstrated that infinitesimal geometric imperfections are responsible for this dramatic discrepancy.

However, traditional fabrication methods introduce uncontrolled, stochastic defect fields, making it difficult to establish deterministic links between imperfection geometry and structural response. By fabricating shells with prescribed, well-characterized imperfection fields, we enable systematic experimental interrogation of imperfection-sensitive buckling. This bridges the gap between theoretical predictions and real-world structural behavior.

This approach has been pioneered by a sustained body of experimental work, primarily from the group of Reis and collaborators, who developed silicone-based hemispherical shells as a model

system for studying imperfection-sensitive buckling. Their decade-long contributions include the fabrication of thin elastic shells by coating curved surfaces [Lee et al., *Nature Communications*, 2016], the first quantitative prediction of knockdown factors for shells with precisely engineered dimple-like imperfections [Lee et al., *Journal of Applied Mechanics*, 2016], characterization of the knockdown factor plateau for large-amplitude defects [López Jiménez et al., *Journal of Applied Mechanics*, 2017], buckling under probing forces [Marthelot et al., *Journal of Applied Mechanics*, 2017], probing the buckling of pressurized spherical shells [Abbasi et al., *Journal of the Mechanics and Physics of Solids*, 2021], comparison of dimpled versus bumpy defects [Abbasi et al., *Journal of Applied Mechanics*, 2023], probabilistic buckling of shells with distributions of defects [Derveni et al., *Philosophical Transactions of the Royal Society A*, 2023], the dominance of the most severe imperfection in multi-defect shells [Derveni et al., *Mechanics of Materials*, 2025], and defect–defect interactions [Derveni et al., *Journal of Applied Mechanics*, 2025]. Collectively, these studies have established a rigorous experimental and computational framework for understanding imperfection-sensitive buckling. However, all of the imperfection geometries studied to date have been localized — single or multiple discrete dimples or bumps — fabricated using indentation, 3D-printed molds, or flexible substrates.

The second purpose is practical: in real structures, imperfections are spatially distributed and arise from manufacturing variability, transport, loading, and environmental factors. The localized imperfections studied in prior work do not capture the global, multi-modal character of realistic defect fields. Our vibration-assisted method is, to our knowledge, the first fabrication approach capable of producing controlled, spatially distributed imperfection fields whose geometry follows well-defined modal symmetries. This enables experimental access to a class of imperfection patterns that has been studied theoretically but has remained largely inaccessible experimentally.

These motivations are now explicitly stated in the revised Introduction (see the added historical context in response to **R1C3**). The Discussion has also been expanded to articulate broader applications in programmable materials and soft robotics (see response to **R2C6**), where the imposed thickness variations can be reinterpreted as architected features for encoding mechanical functionality.

These studies have established an experimental framework for imperfection-sensitive buckling of spherical shells, including quantitative prediction of knockdown factors for precisely engineered dimple-like defects [21], characterization of large-amplitude defect plateaus [22], buckling under probing forces [18], comparison of dimpled versus bumpy defects [19], probabilistic buckling of shells with distributions of defects [23], and defect–defect interactions in multi-defect shells [24, 25]. While recent works have begun investigating multiple interacting imperfections [24, 26], it remains difficult for such fabrication methods to reproduce the complex, global imperfection fields that arise naturally from manufacturing variability. Moreover, constructing dedicated fabrication setups for all possible imperfection geometries remains impractical.

R3C2. Methodological Simplicity: The methodology—casting silicone onto a thick elastic mold excited by a speaker, with vibration-induced flow during curing creating thickness variations—is overly simplistic. The manuscript does not provide sufficient scientific merit or rigor in the experimental design or analysis.

The simplicity of the setup is a deliberate design choice as it enables accessibility, scalability, and reproducibility without requiring specialized equipment such as 3D printers, clean rooms, or precision machining beyond the initial metal sphere.

We have significantly expanded upon the underlying physics of this vibration-assisted manufacturing procedure. The fabrication process involves the coupling of several distinct physical phenomena: structural vibration of a viscoelastic hemispherical mold, oscillatory and secondary streaming flow in a thin liquid layer, free-surface evolution under the combined action of vibration-induced pressure and gravity, and time-dependent curing kinetics. In the revised manuscript, we have developed and presented a rigorous analysis of this process:

1. A weakly nonlinear perturbation analysis (Appendix I of this rebuttal and SI Section 5) identifies the secondary streaming flow driven by Reynolds stresses as the mechanism responsible for material accumulation at the antinodes. This analysis provides both a qualitative explanation (why fluid accumulates at antinodes) and a quantitative scaling law for the free surface deflection amplitude in terms of the governing physical parameters.
2. The excitation frequencies are not chosen empirically but are informed by finite element modal analysis of the mold (SI Section 4). Quantitative agreement between FE-predicted mode shapes and experimentally realized thickness fields is demonstrated through circumferential intensity comparisons (RMSE) and Fourier spectral analysis (SI Section 3.1, SI Fig. 10).
3. Statistical repeatability is established through Pearson correlation analysis across multiple specimens fabricated using different molds under identical conditions (SI Section 1.5, SI Fig. 3).
4. Control experiments without acoustic excitation quantify the baseline thickness uniformity and confirm that non-axisymmetric features are absent in statically cast shells (SI Section 2.1).
5. Quasi-static buckling experiments under controlled pressure loading quantify the knockdown factor as a function of both excitation frequency and volume, with pre-buckling stiffness independently measured (SI Section 5, SI Table 1).

R3C3. Incremental Advancement: When compared to existing studies (e.g., Anna Lee et al., "Fabrication of slender elastic shells by the coating of curved surfaces," Nature Communications, 7(1):11155, 2016; Anna Lee et al., "The geometric role of precisely engineered imperfections on the critical buckling load of spherical elastic shells," Journal of Applied Mechanics, 83(11):111005, 2016), the current work appears to be incremental and closely follows established principles.

We respectfully disagree that the present work is incremental. While we build upon the silicone shell casting platform established by Lee et al. (2016), our work introduces a fundamentally new fabrication capability and addresses a qualitatively different class of imperfection geometries. Further, the new technique of vibration-assisted manufacturing is broadly applicable to problems beyond shell buckling.

The key distinctions are as follows:

1. All prior experimental studies in this field — including the works cited by the reviewer, which we have cited in the manuscript — have been limited to localized imperfections: single or multiple discrete dimples or bumps introduced via indentation, 3D-printed mold features, or probe-based techniques. These geometries, while valuable for establishing quantitative knockdown factor relationships, do not capture the global, spatially distributed character of imperfection fields that arise in real structures from manufacturing variability. Our method is, to our knowledge, the first to produce controlled, global imperfection fields with prescribed

modal symmetry. This opens an entirely new class of imperfection geometries to systematic experimental study.

2. Rather than mechanically imposing a geometric feature onto a mold, our approach exploits vibration-driven fluid dynamics to self-organize the thickness field during casting. The underlying mechanism — secondary streaming flow driven by Reynolds stresses — is fundamentally different from any prior shell fabrication method. We have developed a weakly nonlinear analysis (SI Section 5) that identifies this mechanism and provides scaling predictions. This represents a new connection between fluid mechanics and structural imperfection patterning that did not exist in prior work.
3. Prior methods require fabricating a new mold or indentation setup for each imperfection geometry. In our approach, the imperfection pattern is selected by changing the excitation frequency (a digital parameter), and its severity is tuned continuously by adjusting the speaker volume. Mixed-mode and non-periodic imperfection fields can be generated through broadband excitation without any physical modification to the setup. This level of tunability and flexibility is unprecedented in the shell buckling literature.
4. The method requires only a speaker and a cast mold — no 3D printing, precision machining of mold features, or mechanical indentation apparatus. This makes it readily adoptable by other research groups and scalable to different shell geometries and sizes.

R3C4. Lack of Theoretical Analysis: The manuscript lacks theoretical analysis to support parameter selection and optimization during fabrication. This omission limits the scientific contribution and understanding of the process.

We appreciate the reviewer's concern and have addressed it with a substantial theoretical addition. We have performed a weakly nonlinear perturbation analysis of the vibration-driven flow in the liquid silicone layer, presented in full in Appendix I of this rebuttal and included in the revised SI Section 5. This analysis follows established approaches for flows driven by vibrating boundaries (e.g., Hoepffner & Fukagata, *JFM*, 2009) and identifies the physical mechanism responsible for material redistribution during casting.

The analysis reveals three key results. First, the vibration of the mold drives an oscillatory primary flow that generates non-zero Reynolds stresses. These Reynolds stresses, together with an inhomogeneous boundary forcing, drive a steady secondary streaming flow whose pressure field is elevated at the antinodes of the mold's vibration. This explains why material accumulates at the antinodes.

Second, the analysis predicts that the free surface deflection has twice the wavenumber of the mold's vibration pattern. This is a theoretical prediction that arises naturally from the quadratic structure of the Reynolds stresses and is confirmed by our experimental observations.

Third, in the parameter regime relevant to our experiments, the analysis yields a scaling law for the dimensional amplitude of material accumulation: $A \sim \lambda^2 \mu^2 f^2 \epsilon^2 \gamma^{-2} H^{-1}$, where λ is the wavelength of the mold's vibration, μ is the viscosity of the liquid, f is the frequency of the mold's vibration, ϵ is the amplitude of the mold's vibration, γ is the surface tension of the liquid, and H is the nominal thickness of the liquid layer. This scaling law provides direct guidance for fabrication parameter selection: the imperfection amplitude increases with excitation frequency and vibration amplitude, and decreases with surface tension. In practice, the excitation frequencies are selected based on FE modal analysis of

the mold (SI Section 4), while the imperfection severity is controlled continuously via speaker volume, which governs the vibration amplitude ϵ.

This theoretical contribution also addresses related concerns raised by Reviewer 2 (R2C3) and Reviewer 4 (R4C2) regarding the mechanism of material accumulation. The corresponding manuscript text has been revised accordingly (see R2C3 for the added text).

R3C5. Manuscript Preparation: The manuscript contains typographical errors (e.g., "artificial" in the last paragraph), which reflect a lack of careful preparation.

We have thoroughly gone through the paper and corrected typographical errors.

Reviewer 4

This paper presents a novel acoustic-assisted fabrication method for introducing spatially distributed, mode-shaped imperfections into thin shells. This work demonstrates significant innovation in the controllable fabrication of imperfections, surpassing traditional methods for introducing localized defects. It provides a powerful and scalable platform for systematically studying the influence of global imperfection fields on shell buckling. Prior to publication, the authors should consider the following reviewer comments:

We sincerely thank the reviewer for their positive assessment of the work and for recognizing the novelty of the vibration-assisted fabrication method and its potential as a platform for studying global imperfection fields in shell buckling. We are encouraged by the reviewer's support for publication and have carefully addressed each of the following comments to further strengthen the manuscript.

R4C1. It is recommended to more explicitly emphasize in the Introduction or Discussion the unique advantages of this method compared to existing techniques (such as the indentation or flexible mold methods mentioned in references [15-19]) and the research gap it fills.

We have revised both the Introduction and Discussion to more clearly articulate the research gap and the unique advantages of our method. In the revised Introduction, we now provide a comprehensive overview of prior experimental work on imperfection-sensitive shell buckling, highlighting that existing fabrication methods — including indentation, 3D-printed molds, and flexible substrates — have been limited to localized imperfections such as single or multiple discrete dimples or bumps. While these studies have established quantitative relationships between defect geometry and knockdown factors, they cannot reproduce the spatially distributed, global imperfection fields that arise naturally from manufacturing variability in real structures. We have also added the following sentence to make this gap explicit:

While recent works have begun investigating multiple interacting imperfections [27, 28], it remains difficult for such fabrication methods to reproduce the complex, global imperfection fields that arise naturally from manufacturing variability. Moreover, constructing dedicated fabrication setups for all possible imperfection geometries remains impractical.

Our method addresses this gap with several unique advantages over existing techniques. First, the imperfection geometry is selected digitally by changing the excitation frequency, eliminating the need to fabricate a new mold or indentation setup for each pattern. Second, the imperfection severity is tuned continuously via speaker volume, enabling systematic parametric studies. Third, the method naturally produces global, spatially distributed thickness fields governed by the vibrational eigenmodes of the mold, accessing a class of imperfection patterns that was previously inaccessible experimentally. Fourth, mixed-mode and non-periodic imperfection fields can be generated through broadband excitation without any physical modification to the setup (see SI Section 1.6). Lastly, one can easily combine customized mold with vibration to further broaden the spectrum of available imperfection shapes. These advantages are now discussed explicitly in both the Introduction and the Discussion of the revised manuscript (see also our responses to R1C3, R2C6, and R3C1 for the specific text additions).

R4C2. The paper attributes the imperfection formation to secondary mean flow and provides valuable insight into the physical mechanism by observing particle migration towards the antinodes. However,

the in-depth physical explanation for why the flow leads to material accumulation at the antinodes – which seems counter-intuitive given the maximum vibration amplitude at these points – is somewhat underdeveloped. It is suggested to explore this specific flow mechanism in more detail in the Discussion, citing more specific fluid mechanics theories and conducting a more in-depth quantitative analysis. Particularly, attempting to clarify the quantitative relationship between vibration velocity, frequency, and the accumulated thickness/radius is crucial for defect design.

We thank the reviewer for this insightful comment, which directly motivated one of the most substantial additions to the revised manuscript. We have developed a weakly nonlinear perturbation analysis of the vibration-driven flow, presented in full in Appendix I of this rebuttal and included in the revised SI (Section 5). This analysis follows established approaches for flows driven by vibrating boundaries (e.g., Hoepffner & Fukagata, *JFM*, 2009).

The analysis resolves the counterintuitive observation as follows. The mold's vibration drives an oscillatory primary flow at the same frequency and wavenumber as the vibration. This primary flow generates non-zero Reynolds stresses with twice the wavenumber, which, together with an inhomogeneous boundary forcing, drive a steady secondary streaming flow. The pressure associated with this streaming flow is elevated at the antinodes and reduced at the nodes. If the free surface is allowed to deform, it deflects outward above the antinodes — causing fluid to accumulate there, exactly as observed experimentally. The particle tracking experiments (manuscript Fig. 2e) directly visualize this progressive migration driven by the streaming flow.

Regarding the quantitative relationship requested by the reviewer, in the parameter regime relevant to our experiments ($Re = O(10^{-2})$, $Ca = O(10^{2})$, and $k^{*} = O(10^{-1})$), the analysis yields a scaling law for the dimensional amplitude of free surface deflection: $A \sim \lambda^{2}\mu^{2}f^{2}\epsilon^{2}\gamma^{-2}H^{-1}$, where λ is the wavelength of the mold's vibration, μ is the viscosity of the liquid, f is the frequency of the mold's vibration, ϵ is the amplitude of the mold's vibration, γ is the surface tension of the liquid, and H is the nominal thickness of the liquid layer. This provides the quantitative link between vibration parameters and accumulated thickness that the reviewer identifies as crucial for defect design: imperfection amplitude increases quadratically with both frequency and vibration amplitude, and is suppressed by surface tension.

The following text has been added to the manuscript (see also R2C3 for the full added passage):

A theoretical analysis of the flow reveals that the mold's vibrations drive an oscillatory primary flow of the same frequency and wavelength as the vibrations. The primary flow creates Reynolds stresses with half the wavelength that, together with a boundary forcing created by the primary flow, drive a secondary, time-averaged, streaming flow with half the wavelength. The associated pressure would push an undeformed free surface outward at the locations of the antinodes, leading the liquid silicone to redistribute toward the antinodes. See SI section 5 for more details.

R4C3. The imperfection shapes formed in this study are all standard vibration modes of the elastic hemispherical shell structure. The authors should further consider how to generate triangular, rectangular, or even other irregular shapes on the mold, which is of great significance for translating the research findings into practical engineering applications. It is suggested to pre-design the mold with specific geometries based on its vibrational characteristics. Try to give an example.

We thank the reviewer for this creative suggestion. Following this comment, we have experimented with altering the modal response of the mold by introducing geometric features into its shape. Rather than casting the elastic molds through repeated coating of a smooth metal sphere, we first 3D print hemispherical negatives containing polygonal indentations (triangular, square, pentagonal and hexagonal), and use these to fabricate elastic molds with corresponding localized thickness increase on their underside. The rest of the fabrication procedure remains identical: the mold is mounted onto the speaker, and silicone is poured as the mold is excited at a single frequency.

The resulting cast shells (RFig. 8) demonstrate that the material accumulation pattern can be directly controlled by pre-designing the mold geometry. Specifically, the vibration-induced thickness variations concentrate at the antinodes of the modified mold, which coincide with the vertices of the polygonal features. This produces imperfection patterns that break the symmetry of the standard vibrational modes, including configurations with odd numbers of bump-like defects — geometries that are not achievable with the unmodified hemispherical mold vibrating in its natural eigenmodes.

This result demonstrates that the vibration-assisted fabrication method is not limited to standard modal shapes, and that by combining mold geometry design with acoustic excitation, a significantly broader design space of imperfection patterns becomes accessible. A systematic study of how mold geometry modifies the vibrational response and the resulting imperfection fields is an exciting direction for future work.

These results are included in the revised SI (Section 1.7). We have also included this in the Discussion of the manuscript noting the extensibility of the method to non-standard imperfection geometries through mold design.

In the manuscript:
Furthermore, by pre-designing the mold geometry with polygonal features (*e.g.*, triangular, square, pentagonal or hexagonal protrusions), the vibrational response of the mold can be designed to produce non-standard imperfection patterns, including configurations with odd numbers of bump-like defects (see SI section 1.7). This demonstrates that combining mold geometry design with acoustic excitation significantly broadens the accessible design space of imperfection geometries.

In the SI Section 1.7:
Imperfection geometries through mold design
To demonstrate that the vibration-assisted fabrication method is not limited to standard modal shapes, we fabricate elastic molds with pre-designed geometric features. Rather than casting the elastic molds through repeated coating of a smooth metal sphere, we 3D print hemispherical negatives containing polygonal indentations (triangular, square, pentagonal and hexagonal, see SI Fig. 5a,b). These negatives are used to fabricate elastic molds with corresponding localized thickness increase on their underside (SI Fig. 5c). The rest of the fabrication procedure remains identical: the mold is mounted onto the speaker, and silicone is poured as the mold is excited at a single frequency. The resulting cast shells (SI Fig. 5d) demonstrate that material accumulation concentrates at the antinodes of the modified molds, which coincide with the vertices of the polygonal features. This produces imperfection patterns that break the symmetry of standard vibrational modes, including configurations with odd numbers of bump-like defects that are not achievable with the hemispherical mold.

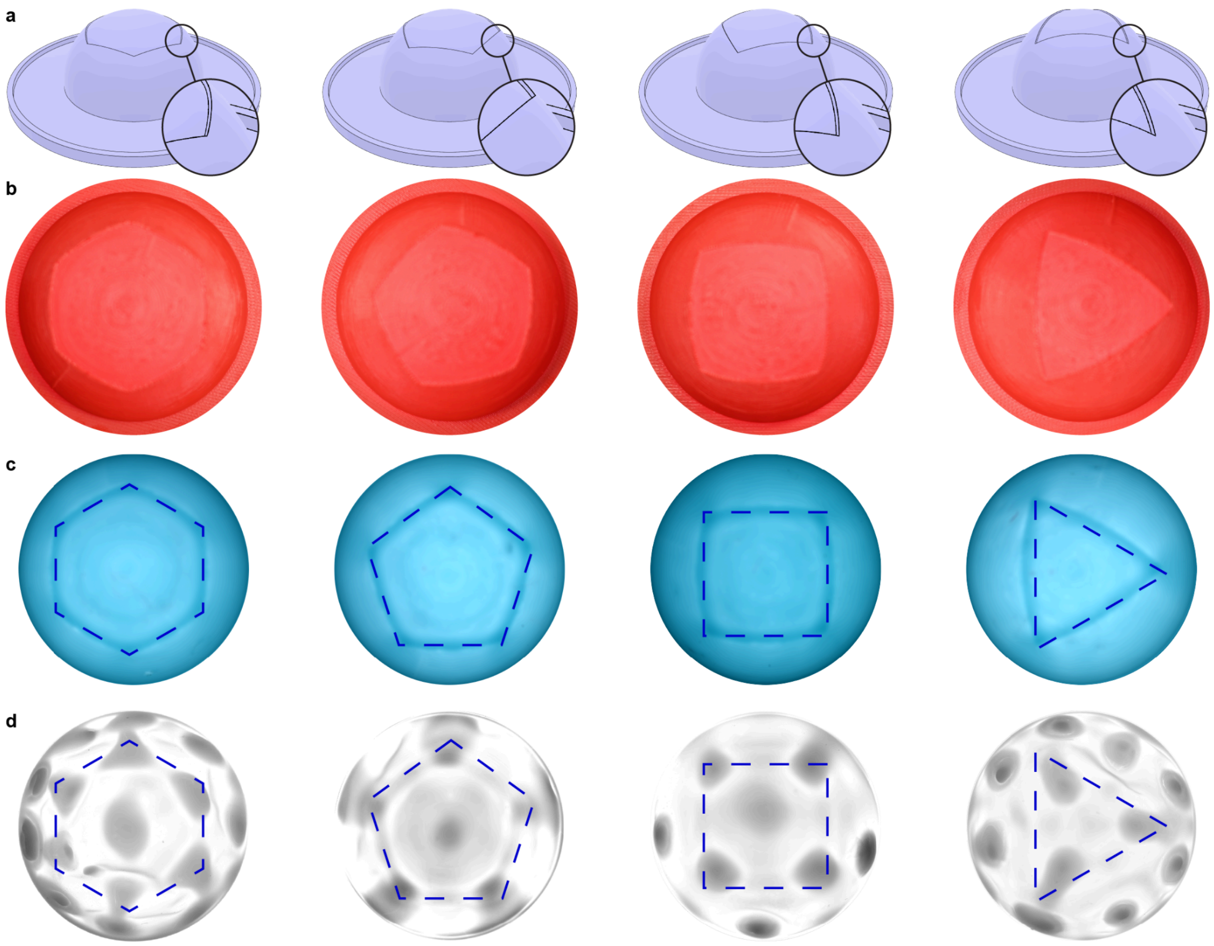

RFig. 8 [SI Fig. 5] **Casting with elastic molds featuring polygonal features.** a,b) Design and 3D printed hemispherical negatives with triangular, square, pentagonal and hexagonal indentations of 0.5mm. c) Elastic molds where thickness is locally increased from the underside. d) The resulting cast shells showing that material accumulation is governed by the vertices of the polygonal features.

Appendix I: Analysis of the fluid

The vibration of the mold drives a flow in the liquid silicone layer that is ultimately responsible for the steady deflection of the liquid silicone's free surface. To gain insight into the flow mechanisms that lead to the steady wavy free surface, we develop and analyze a minimal model that captures the essential physics of the problem. We particularly wish to understand why the free surface's wavenumber is twice that of the mold's vibration pattern, and why the free surface deflects outward at the antinodes of the mold's vibration.

Consider a two-dimensional layer of liquid of nominal thickness H that is bounded below by a vibrating surface and above by a free surface (RFig. 9). The bottom boundary has a prescribed deformation, and the free surface deforms in response to the flow. We take the bottom boundary's deflection to be given by a standing wave of frequency ω, wavenumber k, and amplitude ϵ,

$$h_L(x,t) = \epsilon \sin(\omega t)\cos(kx).$$

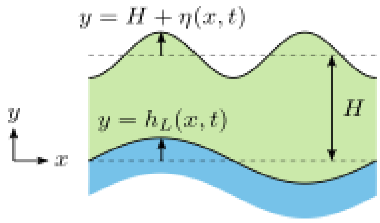

RFig. 9. [SI Fig. 12] Model problem for the flow of the liquid silicone. A fluid (green) with a free surface is driven by the motion of a solid boundary (blue).

The liquid silicone in the experiment is non-Newtonian, but its viscosity is weakly affected by shear [Lee, Nat. Comm., 7.1 (2016); Lukin, Polymers, 12.10 (2020)], so we treat it as Newtonian for simplicity. Additionally, although the liquid silicone's properties change as it cures, this occurs on a time scale that is much slower than any time scales relevant to the flow, so we treat the liquid's properties as constant.

We perform a weakly nonlinear analysis to gain insight into the flow. This approach has been used in the past to gain insight into other flows driven by vibrating boundaries [Hoepffner, Jerome, Koji, Journal of Fluid Mechanics, 635 (2009)]. Lengths are scaled by H, time by ω^{-1}, velocity by ωH, and pressure by $\mu\omega$, with μ the fluid's viscosity. From here onward, all quantities are dimensionless. We expand the fluid velocity and pressure fields, as well as the deflection of the free surface η from its mean height, in powers of $\epsilon^* = \epsilon/H$,

$$\vec{u} = \epsilon^* \vec{u}_1 + \epsilon^{*2} \vec{u}_2 + O(\epsilon^{*3}),$$
$$p = \epsilon^* p_1 + \epsilon^{*2} p_2 + O(\epsilon^{*3}),$$
$$\eta = \epsilon^* \eta_1 + \epsilon^{*2} \eta_2 + O(\epsilon^{*3}).$$

Substituting these expansions into the Navier-Stokes equations and grouping terms of the same order, the primary flow satisfies

$$\nabla \cdot \vec{u}_1 = 0,$$

$$Re\, \partial_t \vec{u}_1 = -\nabla p_1 + \nabla^2 \vec{u}_1.$$

The secondary flow satisfies

$$\nabla \cdot \vec{u}_2 = 0,$$

$$Re\, \partial_t \vec{u}_2 = -\nabla p_2 + \nabla^2 \vec{u}_2 - Re\, \vec{u}_1 \cdot \nabla \vec{u}_1.$$

Note that the primary flow appears as a forcing term for the secondary flow. In the experiment, the Reynolds number $Re = \rho\omega H^2/\mu$ is $O(10^{-2})$, with ρ being the fluid's density.

The full flow field satisfies no-slip and no-penetration boundary conditions at the bottom boundary, while at the free surface it satisfies normal and tangential stress balances. Since in the experiment the free surface of the liquid silicone interfaces with air, the tangential stresses there can be ignored. Similarly, the normal stress balance simplifies to a balance between the normal stress in the liquid and the stress due to surface tension. Additionally, the free surface satisfies a kinematic boundary condition to ensure that its motion is consistent with that of the underlying fluid.

For our purposes, it will suffice to analyze the case where the free surface is fixed to be flat. The resulting pressure distribution at the free surface will then indicate how the free surface would deform once allowed to.

We start by analyzing the primary flow. Its governing equations are all linear and homogeneous, as are its boundary conditions, except for the inhomogeneous no-slip/no-penetration condition at the bottom boundary, which in dimensionless form reads

$$\vec{u}_1 = \cos(t)\cos(k^* x)\,\hat{j} \qquad \text{at } y = 0.$$

This simply reflects that the primary flow is driven entirely by the velocity of the bottom boundary. Because of the linearity of the first-order equations, all primary variables will be sinusoidal in time and space with dimensionless frequency 1 and wavenumber k^*. As a result, there is no mean flow, and the free surface does not have a mean deflection at first order. To determine the mean deflection of the free surface, we must move on to the secondary flow.

The secondary flow's governing equations and boundary conditions have inhomogeneities stemming from the primary flow. In particular, the time-averaged flow satisfies

$$0 = -\nabla < p_2 > + \nabla^2 < \vec{u}_2 > - Re < \vec{u}_1 \cdot \nabla \vec{u}_1 >,$$

where $<\cdot>$ denotes time-averaging. Since the primary flow is sinusoidal in time, the associated Reynolds stresses (which lead to the forcing term above) are non-zero, and they create a mean

secondary flow, which we will call the streaming flow. In addition, the streaming flow has one inhomogeneous boundary condition,

$$<\vec{u}_2> \;=\; <-\frac{1}{\epsilon^*}\partial_y \vec{u}_1 h_L> \;=\; -\partial_y <\vec{u}_1 \sin(t)> \cos(k^* x) \text{ at } y = 0,$$

which we will refer to as the boundary forcing. Since the x-component of the primary velocity is zero at $y = 0$, continuity implies that only the x-component of the above boundary condition for the streaming flow is inhomogeneous.

The pressure associated with the streaming flow leads to steady deformation of the free surface. If we can determine the spatial structure of the streaming flow's pressure field, then we can determine the form of the free surface's mean deflection. Doing so requires knowledge of the Reynolds stresses and boundary forcing, so we return to the primary flow.

Rather than precisely solving for the flow variables, we will instead deduce the structure of the free surface's mean deflection using physical arguments and the basic mathematical structure of the problem. Letting ψ denote the stream function, it follows that the primary flow's stream function takes the form

$$\psi_1(x, y, t) = f(y) \sin(k^* x + \phi_x) \cos(y + \phi_t),$$

where f is a yet-unknown function, and ϕ_x and ϕ_t are yet-unknown phases. Then the two velocity components are

$$u_1(x, y, t) = \partial_y \psi_1(x, y, t) = f'(y) \sin(k^* x + \phi_x) \cos(t + \phi_t),$$
$$v_1(x, y, t) = -\partial_x \psi_1(x, y, t) = -k^* f(y) \cos(k^* x + \phi_x) \cos(t + \phi_t).$$

The forcing due to the Reynolds stresses is then

$$-<\vec{u}_1 \cdot \nabla \vec{u}_1> = \frac{1}{4} k^* (f f'' - f'^2)\ \sin(2(k^* x + \phi_x))\ \hat{i} - \frac{1}{2} k^{*2} f f' \hat{j},$$

and the inhomogeneous part of the streaming flow's boundary condition is

$$-\partial_y < u_1 \sin(t) > \cos(k^* x)|_{y=0} = \frac{1}{2} f''(0) \sin(\phi_t)[\frac{1}{2} \sin(2k^* x) \cos(\phi_x) + \cos^2(k^* x) \sin(\phi_x)].$$

Two features stand out. The first is that the y-component of the forcing from the Reynolds stresses is constant in x, so it will not tell us anything about the mean deflection of the free surface. The second is that the x-component of the forcing from the Reynolds stresses is purely sinusoidal in x with a wavenumber $2k^*$; the same is true for the boundary forcing. Therefore, the mean deflection of the free surface will also be sinusoidal in x with a wavenumber $2k^*$. This is exactly what we see in the experiment: the mean deflection of the free surface has twice the wavenumber of the mold's vibration

pattern. The remaining element left to determine is the position of the free surface's mean deflection pattern relative to the bottom boundary.

To this end, we appeal to physical arguments to first determine the spatial phase ϕ_x. Because the problem has a reflection symmetry in the x-direction, from the inhomogeneous boundary condition for the streaming flow we deduce that $\phi_x = 0$ (otherwise it would have a non-zero spatial mean). We can also come to this conclusion by considering the physics of the primary flow. Where the bottom boundary moves up, the fluid is pushed up with it, and where it moves down, the fluid is pulled down with it. By symmetry, the streamlines above the antinodes of the bottom boundary must be vertical. Recalling that the coordinate system was chosen so that the shape of the bottom boundary is $\propto \cos(k^* x)$, it follows that $\phi_x = 0$. Thus, the pattern of the forcing due to the Reynolds stresses, as well as the pattern of the boundary forcing, are $\propto \sin(2k^* x)$.

Knowing that the two forcings are $\propto \sin(2k^* x)$ is not enough, however, since the sign of the pre-factor determines the spatial phase of the forcings. To determine the pattern of the forcing, we require additional knowledge of the primary flow. Where the bottom boundary moves up, the fluid is pushed up with it, which requires an increased pressure locally. Conversely, where the bottom boundary moves down, the fluid is pulled down with it, which requires a decreased pressure locally. The resulting pressure gradient leads to a circulating flow, as sketched in RFig. 10.

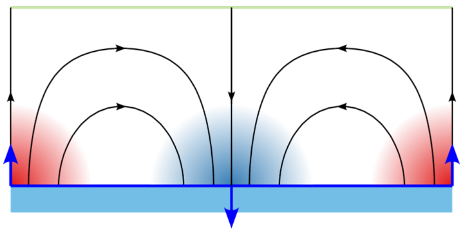

RFig. 10. [SI Fig. 13] Sketch of the primary flow's pressure distribution along the vibrating lower boundary. Red indicates high pressure while blue indicates low pressure. The blue arrows indicate the instantaneous motion of the bottom boundary. The pressure gradient leads to a circulating flow, as shown by the streamlines.

The qualitative picture of the primary flow is sufficient to determine the pattern of the forcing. The x-component of the forcing due to the Reynolds stresses is the mean of $-\vec{u}_1 \cdot \nabla u_1$, which is the negative of the derivative of the horizontal velocity in the direction of the streamlines. Following the pattern of the streamlines, we deduce that the x-component of the forcing is directed toward the antinodes of the bottom boundary. Thus, the forcing due to the Reynolds stresses is $\propto \sin(2k^* x)$ with a negative pre-factor. The Reynolds stress forcing is shown with red arrows in RFig. 11. For the boundary forcing, we require $f''(0)$, which is related to $\partial_y u_1|_{y=0}$. From the pattern of the primary streamlines in RFig. 10, we deduce that $f''(0) > 0$. Additionally, we also require the temporal phase ϕ_t. For the low Reynolds number in the experiment, the low amount of inertia causes the primary flow to lag the motion of the boundary by a small amount, so ϕ_t is small and positive. (In fact, $\phi_t = O(Re)$, so the boundary forcing and the forcing due to the Reynolds stresses are of the same

order.) The resulting boundary condition for the streaming flow is shown with blue arrows in RFig. 11.

Because of the sinusoidal nature of the boundary forcing and the symmetry of the problem, the streamlines of the streaming flow must be vertical above the nodes and antinodes of the bottom boundary's vibration, with the direction of the flow as sketched in RFig. 11. From continuity, it follows that the flow takes on a circulatory nature, consisting of circulation cells of alternating sense. To determine the pressure distribution along the top surface, let us focus on the leftmost circulation cell in RFig. 11. As the fluid rises from the bottom, the forcing due to the Reynolds stresses accelerates the fluid leftward. Once the fluid starts to approach the upper-left corner of the cell, it must turn downward, which requires that it be accelerated rightward against the forcing of the Reynolds stresses. Thus, the pressure must increase locally above the antinode. The same logic applies to the other cells, leading to the pressure distribution sketched in RFig. 11: relatively high pressure above the antinodes, and relatively low pressure above the nodes.

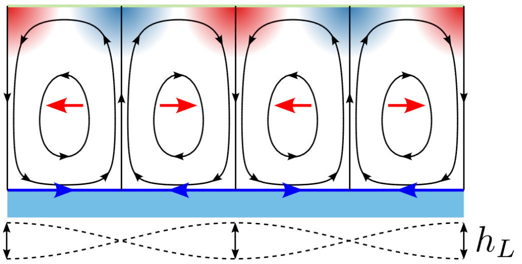

RFig. 11. [SI Fig. 14] Sketch of the streaming flow's pressure distribution along the free surface. Red indicates high pressure while blue indicates low pressure. The blue arrows indicate the boundary condition at the lower boundary, which leads to the circulating flow shown by the streamlines. The red arrows show the x-component of the forcing due to the Reynolds stresses. The vibration pattern is shown at the bottom for reference.

To summarize, the vibration of the bottom boundary drives a time-periodic circulating flow in the fluid. This circulating flow has non-zero Reynolds stresses with twice the wavenumber that, together with a boundary forcing, create a streaming flow. The spatial patterns of the forcings and streaming flow lead to relatively high pressure above the bottom boundary's antinodes and relatively low pressure above the nodes. If the upper surface is allowed to move, it will respond to this pressure distribution, deflecting upward above the antinodes and downward above the nodes. In other words, fluid accumulates above the antinodes of the bottom boundary's vibration, just as in the experiment.

The mechanism described above sets the free surface into motion. Once it is in motion, it will modify the flow. Depending on the values of the parameters, the flow may take on a different form than what has been sketched above. The governing dimensionless parameters are the Reynolds number Re, the capillary number $Ca = \mu\omega H/\gamma$ (where γ is the surface tension), the wavenumber k^*, and the amplitude of vibration ϵ^* of the bottom boundary. In the experiment, we have $Re = O(10^{-2})$, $Ca = O(10^2)$, and $k^* = O(10^{-1})$. In this parameter regime, according to the weakly nonlinear theory, the dimensionless amplitude of deflection of the free surface scales as $A^* \sim Re^0 Ca^2 k^{*-2} \epsilon^{*2}$. In dimensional terms, the amplitude scales as $A \sim \lambda^2 \mu^2 \omega^2 \epsilon^2 \gamma^{-2} H^{-1}$, where we have used the wavelength λ of the vibration instead of the wavenumber k. Note that this assumes that the frequency, wavelength, and amplitude of the vibration can be controlled independently.

Appendix II: Measuring fabricated shell thickness profile

This section analyzes the correlation between the thickness profile of the fabricated imperfect shells and the modal shape resulting from linear perturbation analysis of the elastic hemispherical mold. In doing so, the section demonstrates that 1) digital photography can accurately capture the thickness profile of elastomeric shells, 2) the fabrication method can produce repeatable specimens with or without defects, and 3) that resulting shapes can be inferred by means of linear modal analyses.

The following text is inserted into the SI.

Destructive method

First we discuss the use of destructive measurement methods to capture the thickness profile of fabricated shells. To accurately measure the thickness variations in the shells, we extract strip segments (RFig. 12a) and measure the cross section thickness as a function of the polar angle using an optical microscope. We conduct this with three near-perfect shells fabricated without acoustic excitation (*i.e.*, static casts) but otherwise using the identical setup as our subsequent fabrication protocols (*i.e.*, thick elastic hemispherical mold atop the speaker). The same destructive measurement method is applied to specimens featuring 8 visible bump-like defects. These measurements serve as the ground-truth to correlate pixel intensity in photographs with thickness values.

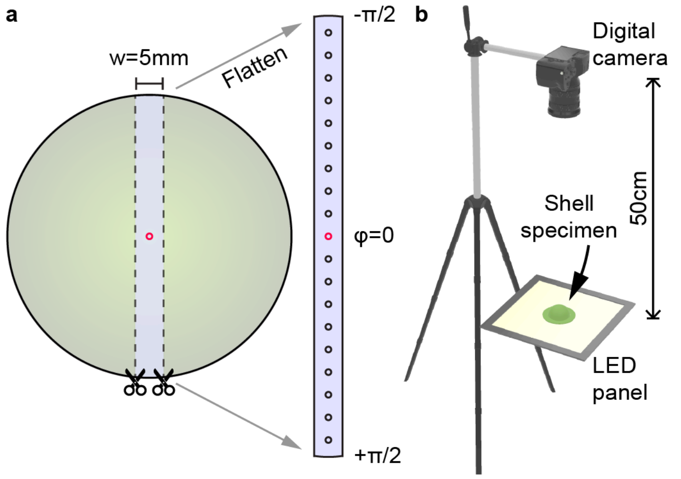

RFig 12. [SI Fig. 6a,b] **Destructive and non-destructive means of shell thickness measurement.** a. The segment extracted from the near-perfect shells. The segment is flattened and its thickness is measured from end to end, covering polar angles from $\varphi = [-\pi/2, \pi/2]$. b. Photography setup to capture shell thickness profile.

Photographic method

Next we inferred the shells' thickness profiles non-destructively through photography. Here, we ensure that the photography conditions including lighting, placement of specimens, and camera setup remain identical across all photos (RFig. 12b). With photography, each fabricated shell, both near-perfect and with-defects, is placed atop an LED panel (HSK A4 LED Light Box) in an otherwise dark room and photographed using a DSLR camera (Nikon D780) from the top at a fixed distance (0.5 meters) using a prime lens (Nikkor 105mm f/2.8G). From the resulting photos, we note that they are largely monochromatic, where there is limited Hue (H) and Saturation (S) distribution (see RFig. 13). This allows us to convert the Value (V) to greyscale, where each pixel has an intensity value I,

without ambiguity. We then proceed to calibrate the images, and construct a correlation between the thickness profile of the shell and the greyscale value of the image ($0 \leq I \leq 1$ where 0 is black and 1 is white).

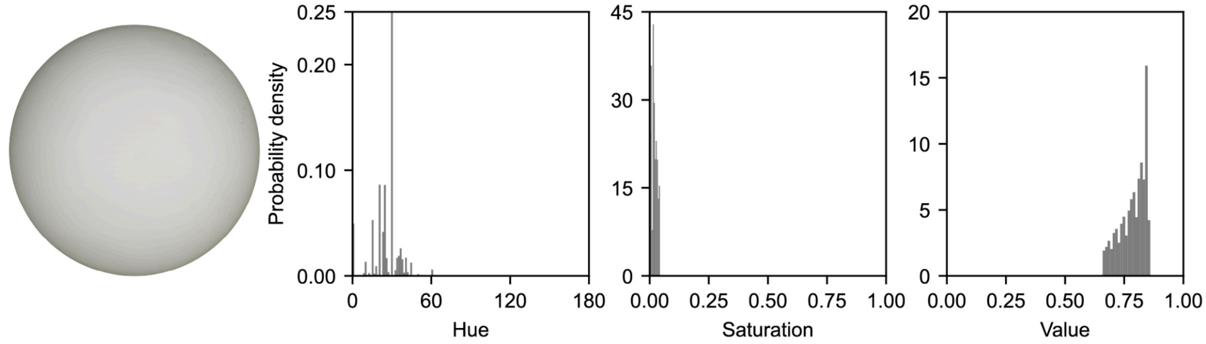

RFig 13. [SI Fig. 7] **Analysis of a photographed shell showing the distribution of Hue, Saturation and Value.**

Calibration photographs and inference of thickness

For calibration, we observe that the photographs of near-perfect shells show a progressive darkening towards the edge (see RFig. 14a). In physical measurements and as well as in literature [Lee, Nat. Comm., 7.1 (2016)], this trend is much less pronounced for the silicone we used (Zhermack Elite Double Fast, Double 32). Rather, this optical effect arises from geometry rather than material variation. Under illumination, rays passing through the shell at the pole intersect the surface approximately orthogonally, whereas rays near the periphery traverse the shell at increasingly oblique angles. As a result, the optical path length through the material increases approximately as $1/\cos\varphi$ where φ is the polar angle. Because transmitted intensity decays exponentially with optical path length according to Beer–Lambert attenuation, this geometric increase in path length leads to systematic edge darkening. We define a multiplicative correction filter $F(\varphi)$ such that the corrected intensity $I_c(\varphi)$ equals to $I_{original}(\varphi) \cdot F(\varphi)$, where

$$F(\varphi) = \exp\left[K\left(\frac{1}{\cos\varphi} - 1\right)\right]. \qquad \text{R.Eq. 2}$$

The correction strength factor K is chosen to empirically fit the photographed thickness of the near-perfect shells to the direct microscopy measurements and remains constant across all images (RFig. 14b).

Next, the photographs are inverse-orthographically remapped to remove distortion arising from projecting a hemisphere (of the shell) to the flat plane of the camera sensor (RFig. 14c). Under orthographical mapping, the pixels are expanded around the equator and/or compressed near the zenith in the radial direction as a function of the polar angle φ. To correct for this, the new radial coordinate of the inverse mapping equals to

$$r_{new} = 2\frac{R}{\pi}\arcsin\left(\frac{r_{old}}{R}\right) \qquad \text{R.Eq.3}$$

where R is the radius of the hemisphere.

Finally, to correlate thickness, t, with respect to light intensity, we refer to the Beer–Lambert law for a uniform material, $I \approx I_0 \exp(-\mu t)$, where with μ is the attenuation coefficient, I is the intensity and I_0 is the incident intensity. Rearranging to isolate the thickness,

$$t \approx -1/\mu \log\left(I/I_0\right), \quad \text{R.Eq. 4}$$

and we fit the attenuation coefficient μ and the incident intensity I_0 to the measurements of the shells discussed above. Specifically, they are empirically fitted to the destructive measurements of the imperfect shells, in particular, the ones with 8 bump-like defects. This is due to the fact that imperfect shells possess a larger range of thickness values whose extrema are thinner and thicker than the near-perfect shells at different points on the shell. As a result, the attenuation coefficient $\mu \approx 1.40m^{-1}$ and $I_0 = 1.264$. Using this, we plot the thickness profile along a single azimuthal angle picked to align with one of the bump-like defects, from zenith, $\varphi = 0$, to the horizon, $\varphi = \pi/2$, for near-perfect shells and for ones with 8 bump-like defects (RFig. 14d). The accuracy of the photography method allows us to rapidly measure the thickness of shells without the need of destructive methods or CT scanning.

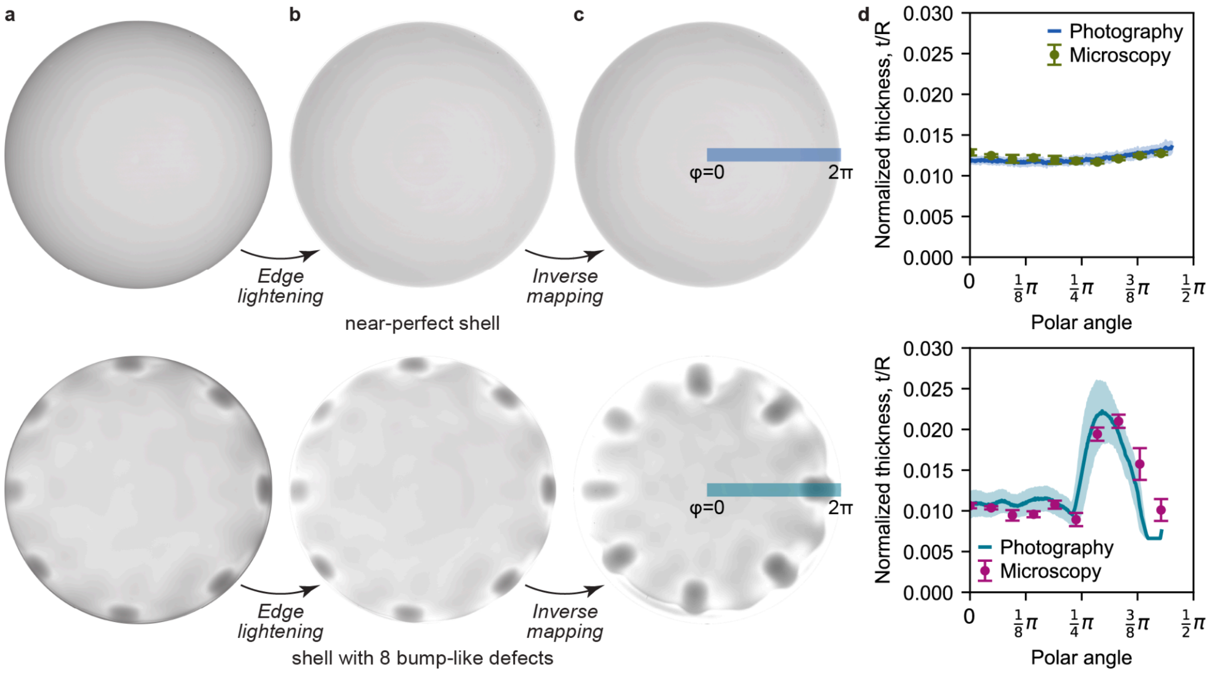

RFig 14. [SI Fig. 8] **Processing of photographed shells, both near-perfect and with defects.** a. Greyscale version of the photographs, b. corrected for edge darkening, c. inverse orthographic remapping and d. thickness profile along one azimuthal angle across polar angles from 0 to π/2 as directly measured and as inferred through photography.

Appendix III: Robustness and repeatability

To demonstrate the robustness and repeatability of the fabrication method, we fabricate three near-perfect and three shells with 8 bump-like defects, and calculate the Pearson correlation coefficient within each group. Note that since the lighting condition of the photographs are identical, the intensity of the pixels of the gray-scale images are compared directly. Each pair of images are treated as vectors of pixel intensities. Each image is first converted to a one-dimensional vector by stacking all pixels, and its mean intensity is subtracted to remove global brightness offsets. The

Pearson correlation coefficient p is then calculated as the normalized inner product of the mean-centered vectors, obtained by dividing their dot product by the product of their Euclidean norms,

$$p\left(I_1, I_2\right) = \frac{\sum_{i=1}^{N}\left(I_{1,i}-\bar{I}_1\right)\left(I_{2,i}-\bar{I}_2\right)}{\sqrt{\sum_{i=1}^{N}\left(I_{1,i}-\bar{I}_1\right)^2}\sqrt{\sum_{i=1}^{N}\left(I_{2,i}-\bar{I}_2\right)^2}} \qquad \text{R.Eq. 5}$$

where I_1 and I_2 are the vectors of pixel intensities of two images being correlated, and N is the number of pixels within the circular mask of the photographs. This normalization renders the metric invariant to global intensity scaling and offset.

The correlation between three near-perfect shells and three shells with 8 bump-like defects are calculated based on Eq. 5 (RFig. 15). Within each group, the correlation means are $\bar{p}_0 = 0.936$ and $\bar{p}_8 = 0.638$ for near-perfect and 8 defects respectively, with $p = 1$ representing a perfect correlation. The deviations within each group can be attributed to three primary causes. The first is the time lapsed between silicone mixing and pouring. This delay influences the eventual thickness of the specimen [Lee, Nat. Comm., 7.1 (2016)] and may explain the difference between t8-s3 and the remaining two. The second is the imprecision in positioning and leveling the mold on the speaker. The mold is secured onto a circular PMMA plate, which is bolted to the rim of the loudspeaker. In this setup, the mold may not be perfectly concentric to the diaphragm of the speaker. Depending on the tightness of the bolts, the lip of the mold may be asymmetrically compressed. Both errors may result in the specimen appearing asymmetric. The third is in image processing, specifically, we manually align one bump-like defect along the horizontal axis by rotating the image. Nevertheless, the within-group correlations are substantially higher than cross-group correlations ($\bar{p}_{0-8} =- 0.117$), confirming that the fabrication process produces consistent imperfection patterns.

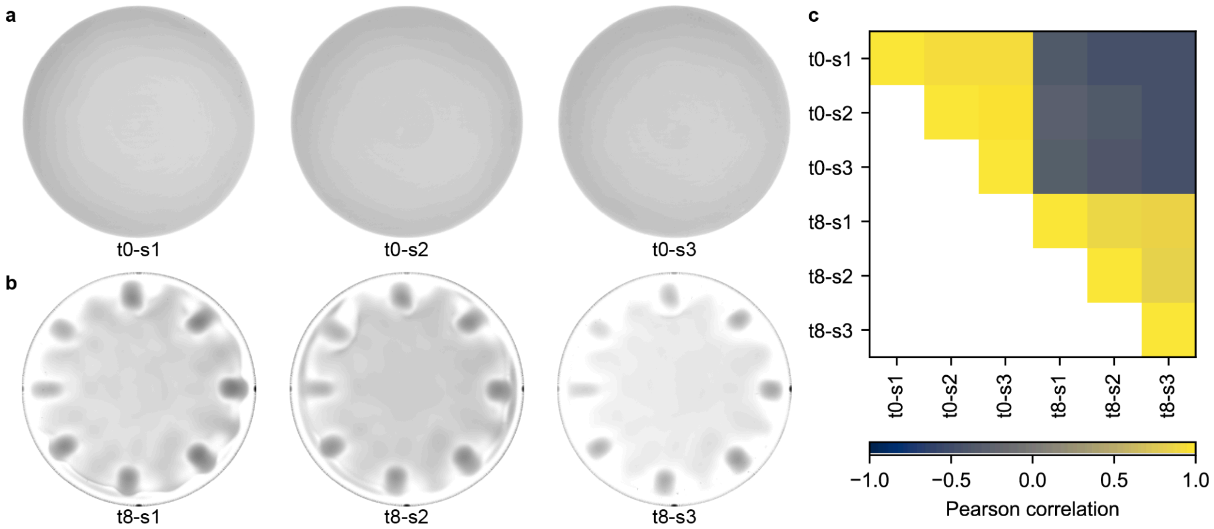

RFig. 15 [SI Fig. 3] **Shape comparison between shells based on photographic evidence.** a. Three near-perfect shells and b. three shells with 8 bump-like defects are compared. c. The resulting Pearson correlation is plotted showing close correlation between shells within each group, and minimal correlation across groups.

Appendix IV: Inference of shell thickness from FE analysis

Here, we measure the similarity between the photographs of the fabricated shells and the resulting shapes of the mold's normal mode. We elect to conduct linear frequency extraction analysis to demonstrate that the specimens can emulate the shape of vibrational modes of the elastic molds. Since all vibrations can be represented as a superposition of natural modes (exactly in the linear case, approximately otherwise), by showing correlation between the fabricated specimens and the FE modal shapes, we demonstrate the versatility of the fabrication method.

In Abaqus, the eigenvectors are normalized such that the maximum of each displacement component is 1. The magnitude of displacement is visualized using greyscale where white and black correspond to zero and maximum displacements respectively. As a result of the above analysis on the fluidic motion, this greyscale value is squared. The resulting images are scaled with the same number of pixels as the photographs of the fabricated shells.
Note that we aim to correlate two different physical quantities, namely the modal displacements of the mold and the thickness of the shells fabricated using the mold. Therefore, rather than comparing the images on a pixel-level, we measure the similarities in the features of the images. Specifically, we assess whether the defects are present at the antinodes of the modal shape.

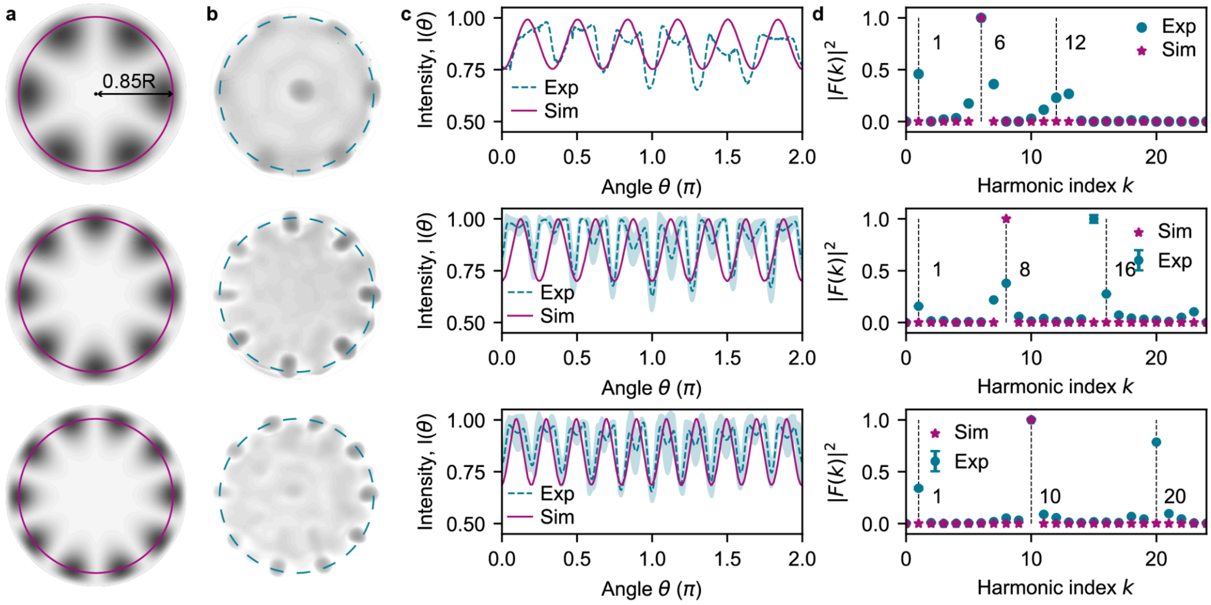

RFig 16. [SI Fig. 10] **Comparison between modal shapes from FE modal analysis and photographs of imperfect shells.** a. Three modal shapes showing 6, 8, and 10 antinodes in a ring near the perimeter where the greyscale represents normalized displacement magnitudes. b. Photographs of fabricated shells with 6, 8 and 10 bump-like imperfections. c. Intensity plots around a ring at a radius of 0.85R. d) Power spectra showing the magnitude of patterns with different periodicities.

We proceed to extract three modal shapes at the requested frequencies; these feature 6,8 and 10 bump-like defects respectively (RFig. 16a). The displacement fields resulting from FE modal analysis are plotted alongside the photographs of the imperfect shells (RFig. 16b). We plot the amplitudes of the intensity as a function of the azimuthal angle θ around a fixed radius (0.85R) to showcase the similarity between the two (RFig. 16c). The RMSE between experimental and simulation intensity measurements for the three specimens are 0.0798, 0.127, 0.0924 respectively. We further observe

slight thickening at the nodes of the modal shape as well. To quantify periodicity, we analyze intensity variations using a Fourier transform. Each image is first reduced to a one-dimensional signal by averaging pixel intensities over a circular ring near the defects ($0.85 \pm 0.5R$), yielding an intensity profile $I(\theta)$. The profile is then mean-subtracted to remove the zero-frequency component and transformed using discrete Fourier transform.

The resulting Fourier coefficients decompose the signal into harmonics corresponding to periodic variations around the circumference. The magnitude of the coefficient of the power spectrum $|F(k)|^2$ at harmonic index k quantifies the strength of a pattern repeating k times over one revolution. When this is applied to the FE modal displacement, one clear peak is present, representing the number of antinodes in that particular mode (RFig. 16d). For the fabricated specimens, the power spectrum similarly shows a local maximum at the requested number of defects. However, other local maxima are also present, specifically, at k=1 and at higher harmonics (e.g., k=12 for the specimens with 6 bump-like defects). The harmonic index of 1 reflects a global imperfection where one side of the hemisphere is thicker than the other. This can be attributed to the imprecision when mounting the mold onto the speaker and the acoustic quality of the speaker itself. The higher harmonics arise from the non-sinusoidal intensity profiles. The signatures around the peaks can be attributed to nonlinearities in the acoustic forcing and geometrically nonlinear structural response at high excitation amplitudes. Altogether, these show that the imprinted imperfections can faithfully retain the feature of the natural vibrational shapes from FE modal analysis.

The above analyses show that the features of the thickness profile resulting from vibration-assisted casting process can be indirectly inferred from the FE modal shape. To do so, one would map the displacement field to greyscale intensity, then apply R.Eq. 4 to calculate the resulting thickness.

Response to the reviewers' comments of the revised manuscript:

Vibration-Assisted Fabrication of Thin Shells with Spatially Distributed Imperfections
by
Ilyes Krida, Jacob Tang, Leo Mangalath, Daniel Floryan, Tian Chen

---

Reviewer #1 (Remarks to the Author):

The authors have addressed all major questions and responded to the points raised. I believe that these substantial revisions and updates have elevated the manuscript to a level suitable for publication in Nature Communications. I recommend the paper for publication.

We wish to thank the reviewer for their favorable feedback.

Reviewer #2 (Remarks to the Author):

I am satisfied with the thorough way in which the authors have addressed most if not all the reviewer comments. I think the elements of novelty have been more clearly specified and the remaining unclear aspects have been addressed, so that I feel it merits publication in NatComm

We wish to thank the reviewer for their favorable feedback.

Reviewer #3 (Remarks to the Author):

After carefully reviewing the revised manuscript, I must unfortunately reiterate my earlier recommendation for rejection. Although the authors have attempted to address my previous comments, the paper still does not, in my view, meet the standards required for publication in NC. In particular, I continue to have the following major concerns: not clear motivation, incremental contribution, and quality of presentation (the overall quality of the manuscript remains below the journal's standards. This includes, but is not limited to, the figures and drawings, many of which are unclear, lack sufficient resolution or labeling, and do not effectively support the main arguments).

Please also the authors shall clarify why corresponding author's affiliation and email adress have been changed?

We thank Reviewer #3 for their time and engagement with the manuscript. We briefly address the points raised. The revised Introduction expands the motivation by clarifying the gap in fabricating globally distributed, rather than localized, imperfections, which is representative of the defect landscapes encountered in real manufacturing. The revised manuscript also includes a new weakly nonlinear flow analysis that explains the pattern-formation mechanism, as well as an extension to mold-designed imperfection geometries that broadens the accessible design space. Figure quality has been improved through clearer labeling, scale bars, and higher-resolution artwork conforming to Nature Communications standards.
Regarding the corresponding author's affiliation and email: the corresponding author (T.C.) is transitioning to a new institution during the review period. Consistent with Nature Communications' editorial guidance, both affiliations are listed on the manuscript -- the original institution where the

work was conducted and the current institution -- and the corresponding author's email has been updated to their current institutional address to ensure reliable long-term correspondence. This change has no bearing on authorship, contributions, data integrity, or any scientific aspect of the work, and all authors have been informed of and approve this update.

Reviewer #4 (Remarks to the Author):

The authors have made an extremely high-level revision in response to my concerns. They clearly articulate the academic contribution of the study, profoundly elucidate the fluid mechanics formation mechanism of the physical phenomenon, and further creatively achieve the fabrication of other irregular shapes. Therefore, I strongly recommend its acceptance for publication in Nature Communications.

We wish to thank the reviewer for their favorable feedback.
